# Dynamic frailty exposure and cognitive aging trajectories: A multicohort study

**DOI:** 10.1002/alz.71821

**Published:** 2026-09-10

**Authors:** Xingjie Wang, Hongtao Hao, Tancheng Jiang, Xutong Yao, Yusheng Duan, Junmei Xia, Cenyi Wang, Jiling Liang

**Affiliations:** ^1^ Department of Physical Education Central South University Changsha China; ^2^ School of Physical Education and Sport Science Soochow University Suzhou China; ^3^ Center for Sports Rehabilitation and Sports Risk Mitigation Research Central South University Changsha China

**Keywords:** CHARLS, cognitive trajectory, dynamic changes, ELSA, frailty, HRS, multicohort study

## Abstract

**INTRODUCTION:**

The cognitive implications of longitudinal frailty exposure remain unclear. We examined associations of baseline frailty status, two‐wave frailty change, and mean frailty burden with annual cognitive change and cognitive aging trajectories.

**METHODS:**

We analyzed data from three nationally representative cohorts: the China Health and Retirement Longitudinal Study, English Longitudinal Study of Ageing, and Health and Retirement Study (*n* = 19,586). Primary linear mixed‐effects models used exposure‐by‐time interactions to estimate differences in annual global and domain‐specific cognitive change. Cohort‐specific estimates were pooled using random‐effects meta‐analysis. Secondary group‐based trajectory modeling (GBTM) identified cognitive trajectory groups, and logistic regression assessed associations with group membership.

**RESULTS:**

Baseline pre‐frailty and frailty were associated with more negative annual global cognitive change versus robust status (both pooled *β* = −0.023 SD/year, 95% CIs −0.030 to −0.016 and −0.031 to −0.014, respectively). Worsening from robust to pre‐frail or frail status was associated with more negative change (pooled *β* = −0.025 SD/year, 95% CI −0.035 to −0.014). Higher mean frailty burden was associated with more negative change. Changes from initial pre‐frailty or frailty showed no clear pooled associations. In secondary GBTM analyses, baseline frailty status, higher mean frailty burden, and frailty worsening were associated with less‐favorable trajectory groups. Frailty improvement was directionally associated with more‐favorable groups.

**DISCUSSION:**

Baseline frailty status, worsening from robust status, and higher mean frailty burden were associated with less‐favorable cognitive aging across complementary rate‐based and trajectory analyses. Repeated frailty assessments may provide information for characterizing cognitive aging.

## Highlights

- Multi‐cohort study of 19,586 adults from China Health and Retirement Longitudinal Study (CHARLS), English Longitudinal Study of Ageing (ELSA), and Health and Retirement Study (HRS).
- Baseline pre‐frailty and frailty were associated with faster cognitive decline.
- Worsening from robust status was associated with faster global cognitive decline.
- Higher mean frailty burden was linked to faster decline and unfavorable trajectories.

## 1 BACKGROUND

As populations age worldwide, frailty has become a major public health concern, particularly among middle‐aged and older adults.^1^, ^2^ Frailty is a multidimensional syndrome characterized by reduced physiological reserves and heightened vulnerability to stressors.^3^, ^4^ It is strongly associated with functional decline, reduced quality of life, and increased mortality risk.^5^, ^6^, ^7^ Accumulating evidence indicates that frailty is associated with cognitive impairment and dementia.^8^ However, despite growing recognition of this association, the extent to which longitudinal frailty exposure relates to subsequent cognitive change remains insufficiently understood. Characterizing these relationships may improve understanding of cognitive aging among middle‐aged and older adults.

To date, most evidence regarding the association between frailty and cognitive decline has relied predominantly on frailty measured at a single time point. Given the dynamic nature of frailty,^9^ baseline assessments alone may fail to capture changes in frailty status or the average burden of health deficits across repeated assessments. Although recent multicohort evidence has demonstrated that changes in frailty status are associated with incident dementia,^10^ dementia represents a relatively late clinical endpoint. Whether baseline frailty and two‐wave frailty measures are associated with distinct trajectories of multidomain cognitive aging remains unclear.

A recent multicohort study used linear mixed‐effects models (LMMs) to examine the association between frailty and longitudinal cognitive function, thereby estimating exposure‐related differences in the average rate of cognitive change.^11^ However, exposure‐related differences in average cognitive change alone do not fully characterize heterogeneity in longitudinal cognitive level‐and‐change patterns.^12^, ^13^ Group‐based trajectory modeling (GBTM) identifies distinct cognitive trajectory groups, capturing heterogeneity that may not be fully characterized by estimates of average cognitive change.^14^, ^15^, ^16^, ^17^ Applying both analytical approaches in large‐scale epidemiological cohorts with repeated frailty assessments and extended follow‐up therefore may provide a broader characterization of the associations between longitudinal frailty exposure and cognitive aging.

To address these gaps, we applied complementary LMMs and group‐based trajectory modeling approaches to harmonized data from three nationally representative longitudinal aging cohorts spanning diverse geographic and healthcare contexts: the China Health and Retirement Longitudinal Study (CHARLS), the English Longitudinal Study of Ageing (ELSA), and the Health and Retirement Study (HRS). Specifically, this study aimed to examine the associations of (1) baseline frailty status, (2) two‐wave frailty change, and (3) mean frailty burden with longitudinal cognitive aging among middle‐aged and older adults.

## 2 METHODS

### 2.1 Study design and population

Data were collected from three prospective, nationally representative cohort studies: CHARLS, ELSA, and HRS.^18^, ^19^, ^20^ To harmonize study periods across cohorts and avoid potential influences of the coronavirus disease 2019 (COVID‐19) pandemic, four pre‐pandemic waves were used from each cohort: waves 1–4 of CHARLS (2011–2018), waves 6–9 of ELSA (2012–2019), and waves 11–14 of HRS (2012–2018). Wave 2 of CHARLS (2013), wave 7 of ELSA (2014), and wave 12 of HRS (2014) were designated as the study baseline. To assess changes in frailty status over time, frailty measures were obtained from the immediately preceding survey: wave 1 of CHARLS (2011), wave 6 of ELSA (2012), and wave 11 of HRS (2012). Subsequent follow‐up surveys were used to track outcomes through the final assessment: wave 4 of CHARLS (2018), wave 9 of ELSA (2019), and wave 14 of HRS (2018).

**RESEARCH IN CONTEXT**

1. **Systematic review**: We searched PubMed and Web of Science for studies of frailty and cognitive decline. Most studies used frailty measured at a single time point, and few examined dynamic frailty in relation to later cognitive aging.
2. **Interpretation**: Baseline pre‐frailty and frailty, worsening from robust status, and higher mean frailty burden were associated with faster annual decline in global cognition. In secondary group‐based trajectory modeling (GBTM) analyses, baseline frailty status and higher mean frailty burden were associated with less‐favorable cognitive trajectory groups that reflected both cognitive level and change.
3. **Future directions**: Future studies should clarify pathways linking frailty progression to cognitive aging and determine in randomized trials whether modifying frailty alters cognitive outcomes. Studies with repeated frailty assessments over longer periods are also needed to examine effect modifiers and longer‐term changes in frailty.

**FIGURE 1:**
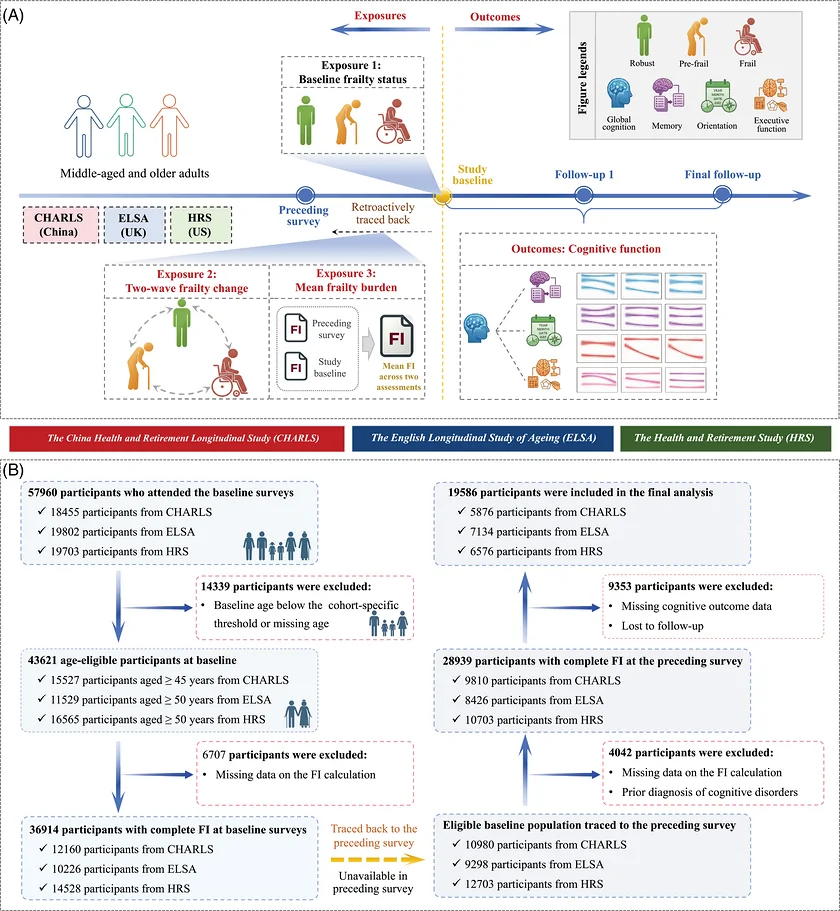
Study design and participant selection flowchart. (A) Study design and analytical framework. (B) Screening flowchart for the study cohorts. CHARLS, China Health and Retirement Longitudinal Study; ELSA, English Longitudinal Study of Ageing; FI, frailty index; HRS, Health and Retirement Study.

The inclusion criteria were as follows: (1) availability of complete data on frailty and cognitive function; (2) age equal to or above the cohort‐specific threshold (≥ 50 years for ELSA and HRS; ≥ 45 years for CHARLS); and (3) no history of physician‐diagnosed cognitive disorders (Table S1). Figure 1 illustrates the study design and population selection process. A total of 19,586 eligible participants were included in the analysis.

### 2.2 Assessment of frailty

Frailty was assessed using the frailty index (FI) derived from the accumulation of multiple age‐related health deficits. Following previously validated procedures,^11^, ^21^, ^22^ FI was constructed using harmonized data from CHARLS, ELSA, and HRS cohorts. A total of 28 items were selected, encompassing diseases, symptoms, disabilities, physical functioning, and depression, while excluding memory‐related conditions (Table S2). Each item was dichotomized using cohort‐specific cutoffs, with 1 indicating the presence of a deficit and 0 indicating its absence. FI was calculated as the number of deficits present divided by 28, yielding a continuous score ranging from 0 to 1, with higher values indicating greater frailty. Cronbach's alpha was calculated within each cohort to evaluate the internal consistency of the 28‐item FI (Table S3). Consistent with prior studies,^5^, ^11^ participants were classified as robust (FI ≤ 0.10), pre‐frail (0.10 < FI < 0.25), or frail (FI ≥ 0.25).

Data from the preceding and baseline surveys were used to evaluate two aspects of frailty exposure: two‐wave frailty change and mean frailty burden. Two‐wave frailty change was defined according to changes in frailty status between the two assessments (Figure S1). Mean frailty burden was operationalized as the mean FI across the two assessments, calculated as follows: (FI at the preceding survey + FI at the baseline survey) / 2, and categorized into cohort‐specific tertiles.

### 2.3 Assessment of cognitive function

Cognitive function was assessed using methods established in previous research.^11^, ^21^, ^22^ Global cognition was derived from three cognitive domains: memory, orientation, and executive function (Table S4). Global cognition was the primary outcome, whereas domain‐specific cognitive scores were secondary outcomes. To facilitate harmonization and comparability across the three cohorts, both global and domain‐specific raw cognitive scores were standardized as z‐scores. Each z‐score was calculated by subtracting the cohort‐specific baseline mean from the individual raw score and dividing by the corresponding cohort‐specific baseline standard deviation.^11^, ^23^, ^24^

### 2.4 Covariates

Covariates included age, sex, education, marital status, smoking status, drinking status, and body mass index (BMI). To ensure data harmonization across cohorts, categorical variables were standardized as follows. Educational attainment was categorized into three levels: below high school, high school, and college or higher. Marital status was categorized as married or partnered and other (separated, divorced, unmarried, or widowed). Smoking status was categorized as never smokers or ever smokers. Ever smokers included former and current smokers. Drinking status was categorized as never drinkers or ever drinkers.

### 2.5 Statistical analysis

Descriptive analyses were used to summarize continuous variables as means with standard deviations (SDs) and categorical variables as counts with percentages. The missingness rates and covariate distributions are detailed in Table S5 and Figure S2. Missing covariate data were handled using multiple imputation by chained equations.

LMMs were used as the primary analysis to estimate exposure‐related differences in annual cognitive change. Models included frailty exposure, follow‐up time, their interaction, the covariates described above, and a participant‐specific random intercept. Follow‐up time was expressed in years since cognitive baseline, and the exposure‐by‐time interaction estimated the between‐group difference in annual cognitive z‐score change. Models were fitted separately in each cohort, and cohort‐specific estimates were pooled using random‐effects meta‐analysis, with I^2^ used to summarize between‐cohort heterogeneity.

As a secondary analysis, GBTM was used to identify distinct longitudinal trajectories of global and domain‐specific cognitive function. Candidate solutions were compared using Akaike and Bayesian information criteria, average posterior probabilities, and group proportions.^25^, ^26^, ^27^ Solutions were considered acceptable when the average posterior probability for each group exceeded 0.70, and each group included at least 2% of participants. Model‐fit indices served as initial guidelines; the final solution was selected based on parsimony and whether the trajectories were distinct, stable, and relevant to the study objective.^17^, ^28^ These trajectories were interpreted as reflecting both cohort‐relative cognitive level and longitudinal change.

Multinomial and binary logistic regression models were used to estimate associations of baseline frailty status, two‐wave frailty change, and mean frailty burden with GBTM‐derived cognitive trajectory membership, expressed as odds ratios (ORs) and 95% confidence intervals (CIs). False discovery rate (FDR) correction was applied to *p*‐values from the fully adjusted GBTM models using the Benjamini–Hochberg method, separately for each frailty exposure.

Several sensitivity analyses were conducted to evaluate the robustness of findings. (i) Because FI cutoff values for frailty status remain controversial, we repeated the primary LMM analyses of global cognition for baseline frailty status and two‐wave frailty change, together with the corresponding secondary GBTM analyses, using an alternative scheme. This scheme defined frail, pre‐frail, and robust statuses as FI > 0.21, 0.10 < FI ≤ 0.21, and FI ≤ 0.10, respectively. (ii) To reduce the potential influence of baseline cognitive level, the secondary GBTM analyses were repeated with additional adjustment for baseline cognitive z‐scores. (iii) To address potential reverse causation, the secondary GBTM analyses were repeated using frailty measures assessed at the preceding survey wave as exposures, with additional adjustments for cognitive z‐scores measured at the same wave as the lagged frailty exposure. (iv) To assess the potential influence of attrition or selection bias, the secondary GBTM analyses were repeated using inverse probability weighting. (v) To assess potential overlap between frailty components and early cognitive pathology, modified FIs were constructed by separately excluding depression/mental health‐related deficits, disability‐related deficits, and physical functioning/mobility‐related deficits, and the secondary GBTM analyses were repeated for each modified FI.

All statistical analyses were performed using R software (version 4.5.2). Two‐sided *p *< 0.05 was considered statistically significant.

## 3 RESULTS

### 3.1 Baseline characteristics of participants in all cohorts

A total of 19,586 individuals were included in the final analysis: 5876 (30.0%) from CHARLS, 7134 (36.4%) from ELSA, and 6576 (33.6%) from HRS. The baseline characteristics are presented in Table 1. Across the three cohorts, 7846 (40.1%) participants were classified as robust, 7281 (37.2%) as pre‐frail, and 4459 (22.8%) as frail at baseline. Compared to robust participants, frail participants were generally older, more likely to be female, and had higher BMI values. Baseline characteristics based on unimputed data are presented in Table S6.

**TABLE 1:** Baseline characteristics of participants for baseline frailty status analyses.

Characteristic	CHARLS (*n* = 5876)			ELSA (*n* = 7134)			HRS (*n* = 6576)		
	Robust	Pre‐frail	Frail	Robust	Pre‐frail	Frail	Robust	Pre‐frail	Frail
n (%)	2757 (46.9%)	2328 (39.6%)	791 (13.5%)	3496 (49.0%)	2234 (31.3%)	1404 (19.7%)	1593 (24.2%)	2719 (41.3%)	2264 (34.4%)
Age, mean (SD), years	57.9 (7.8)	59.8 (8.2)	62.2 (8.4)	65.0 (7.9)	69.2 (8.5)	71.4 (9.6)	71.1 (6.3)	73.2 (6.6)	74.2 (7.5)
Sex, *n* (%)									
Female	963 (34.9%)	1082 (46.5%)	457 (57.8%)	1815 (51.9%)	1290 (57.7%)	911 (64.9%)	903 (56.7%)	1549 (57.0%)	1521 (67.2%)
Male	1794 (65.1%)	1246 (53.5%)	334 (42.2%)	1681 (48.1%)	944 (42.3%)	493 (35.1%)	690 (43.3%)	1170 (43.0%)	743 (32.8%)
Marital status, *n* (%)									
Married or partnered	2541 (92.2%)	2110 (90.6%)	660 (83.4%)	2561 (73.3%)	1472 (65.9%)	755 (53.8%)	1037 (65.1%)	1628 (59.9%)	1076 (47.5%)
Other marital status	216 (7.8%)	218 (9.4%)	131 (16.6%)	935 (26.7%)	762 (34.1%)	649 (46.2%)	556 (34.9%)	1091 (40.1%)	1188 (52.5%)
Education, *n* (%)									
Below high school	2264 (82.1%)	2048 (88.0%)	739 (93.4%)	575 (16.4%)	628 (28.1%)	646 (46.0%)	132 (8.3%)	343 (12.6%)	556 (24.6%)
College or above	69 (2.5%)	33 (1.4%)	8 (1.0%)	932 (26.7%)	396 (17.7%)	133 (9.5%)	586 (36.8%)	746 (27.4%)	316 (14.0%)
High school	424 (15.4%)	247 (10.6%)	44 (5.6%)	1989 (56.9%)	1210 (54.2%)	625 (44.5%)	875 (54.9%)	1630 (59.9%)	1392 (61.5%)
Smoking status, *n* (%)									
Ever smokers	1510 (54.8%)	1138 (48.9%)	310 (39.2%)	1982 (56.7%)	1446 (64.7%)	991 (70.6%)	790 (49.6%)	1509 (55.5%)	1306 (57.7%)
Never smokers	1247 (45.2%)	1190 (51.1%)	481 (60.8%)	1514 (43.3%)	788 (35.3%)	413 (29.4%)	803 (50.4%)	1210 (44.5%)	958 (42.3%)
Drinking status, *n* (%)									
Ever drinkers	1685 (61.1%)	1276 (54.8%)	392 (49.6%)	3254 (93.1%)	1988 (89.0%)	1044 (74.4%)	1036 (65.0%)	1542 (56.7%)	945 (41.7%)
Never drinkers	1072 (38.9%)	1052 (45.2%)	399 (50.4%)	242 (6.9%)	246 (11.0%)	360 (25.6%)	557 (35.0%)	1177 (43.3%)	1319 (58.3%)
BMI, mean (SD), kg/m^2^	23.5 (3.2)	24.1 (3.5)	24.2 (3.5)	27.1 (3.9)	28.7 (4.9)	31.1 (5.7)	27.3 (4.3)	29.8 (5.4)	31.1 (5.8)
Cognitive scores, mean (SD)									
Memory	8.0 (3.4)	7.4 (3.3)	6.5 (3.2)	11.7 (3.3)	10.6 (3.5)	9.4 (3.5)	10.8 (3.3)	9.9 (3.2)	8.9 (3.2)
Orientation	3.3 (1.0)	3.2 (1.1)	2.9 (1.2)	3.9 (0.4)	3.8 (0.4)	3.7 (0.6)	3.8 (0.4)	3.8 (0.5)	3.7 (0.6)
Executive function	5.2 (2.0)	4.8 (2.2)	4.2 (2.4)	6.3 (1.3)	6.1 (1.4)	5.6 (1.9)	5.9 (1.6)	5.6 (1.7)	5.0 (2.0)
Global cognition	16.4 (5.1)	15.2 (5.2)	13.2 (5.3)	21.8 (3.9)	20.5 (4.3)	18.7 (4.7)	19.9 (4.3)	18.9 (4.3)	17.1 (4.6)

Abbreviations: BMI, body mass index; CHARLS, China Health and Retirement Longitudinal Study; ELSA, English Longitudinal Study of Ageing; HRS, Health and Retirement Study; SD, standard deviation.

### 3.2 Associations of frailty exposures with annual cognitive change

**FIGURE 2:**
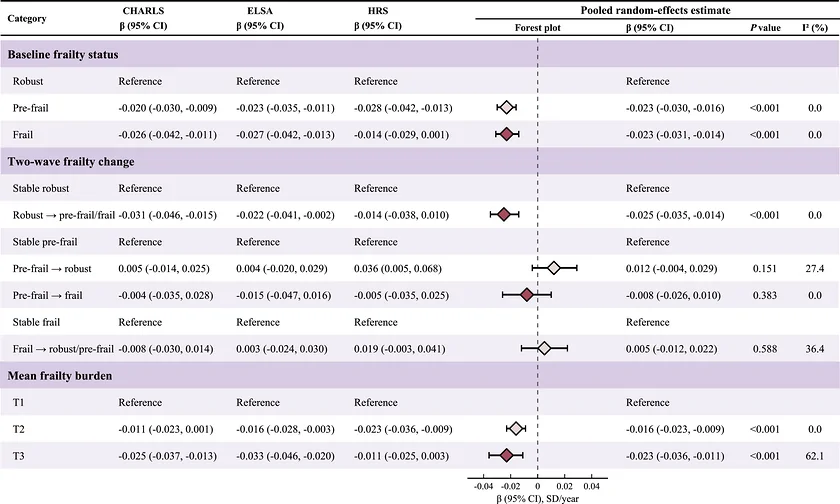
Forest plot for the associations between frailty exposures and annual change in global cognitive z‐score (SD/year). Models were adjusted for age, sex, education, marital status, smoking status, drinking status, and body mass index. The estimates were then meta‐analyzed using random‐effects models. Diamonds represent pooled β coefficients, horizontal lines represent 95% confidence intervals, and the dashed vertical line indicates *β* = 0. I^2^ quantifies between‐cohort heterogeneity. CHARLS, China Health and Retirement Longitudinal Study; CI, confidence interval; ELSA, English Longitudinal Study of Ageing; HRS, Health and Retirement Study; SD, standard deviation.

Figure 2 presents the cohort‐specific and pooled linear mixed‐effects estimates for global cognition. Compared with robust status, baseline pre‐frailty and frailty were associated with more negative annual change in global cognition (pre‐frailty: pooled *β* = −0.023, 95% CI: −0.030 to −0.016, *I^2^* = 0%; frailty: pooled *β* = −0.023, 95% CI: −0.031 to −0.014, *I^2^* = 0%).

Table 2 presents the distribution of two‐wave frailty change between the preceding and baseline assessments. Among participants who were robust at the preceding assessment, 848 (27.8%) in CHARLS, 647 (17.5%) in ELSA, and 501 (26.9%) in HRS worsened to pre‐frail or frail status by baseline. Among participants who were frail at the preceding assessment, 291 (47.6%) in CHARLS, 262 (20.3%) in ELSA, and 285 (14.8%) in HRS improved to pre‐frail or robust status by baseline. Among participants who were initially robust, worsening to pre‐frail or frail status was associated with more negative annual global cognitive change (pooled *β* = −0.025, 95% CI: −0.035 to −0.014, *I^2^* = 0%). Among participants who were initially pre‐frail or frail, patterns of two‐wave frailty change showed no clear pooled associations with annual cognitive change.

**TABLE 2:** Number and percentage of participants by two‐wave frailty change.

Preceding survey		Baseline survey	CHARLS *n* (%)	ELSA *n* (%)	HRS *n* (%)
	↗	Robust	2206 (72.2%)	3049 (82.5%)	1360 (73.1%)
Robust	→	Pre‐frail	767 (25.1%)	624 (16.9%)	469 (25.2%)
	↘	Frail	81 (2.7%)	23 (0.6%)	32 (1.7%)
	↗	Robust	516 (23.3%)	440 (20.5%)	227 (8.2%)
Pre‐frail	→	Pre‐frail	1305 (59.0%)	1355 (63.2%)	1971 (70.8%)
	↘	Frail	390 (17.6%)	350 (16.3%)	587 (21.1%)
	↗	Robust	35 (5.7%)	7 (0.5%)	6 (0.3%)
Frail	→	Pre‐frail	256 (41.9%)	255 (19.7%)	279 (14.5%)
	↘	Frail	320 (52.4%)	1031 (79.7%)	1645 (85.2%)

*Note*. The interval between the preceding and baseline surveys was two years in CHARLS, ELSA, and HRS.

Abbreviations: CHARLS, China Health and Retirement Longitudinal Study; ELSA, English Longitudinal Study of Ageing; HRS, Health and Retirement Study.

Compared with the lowest tertile of mean frailty burden, the middle and highest tertiles were associated with more negative annual change in global cognition (T2: pooled *β* = −0.016, 95% CI: −0.023 to −0.009, *I^2^* = 0%; T3: pooled *β* = −0.023, 95% CI: −0.036 to −0.011, *I^2^* = 62.1%). In secondary analyses of domain‐specific cognition, associations were more consistently observed for memory and executive function than for orientation across cohorts (Tables S7–S9).

### 3.3 Estimated cognitive aging trajectories

**FIGURE 3:**
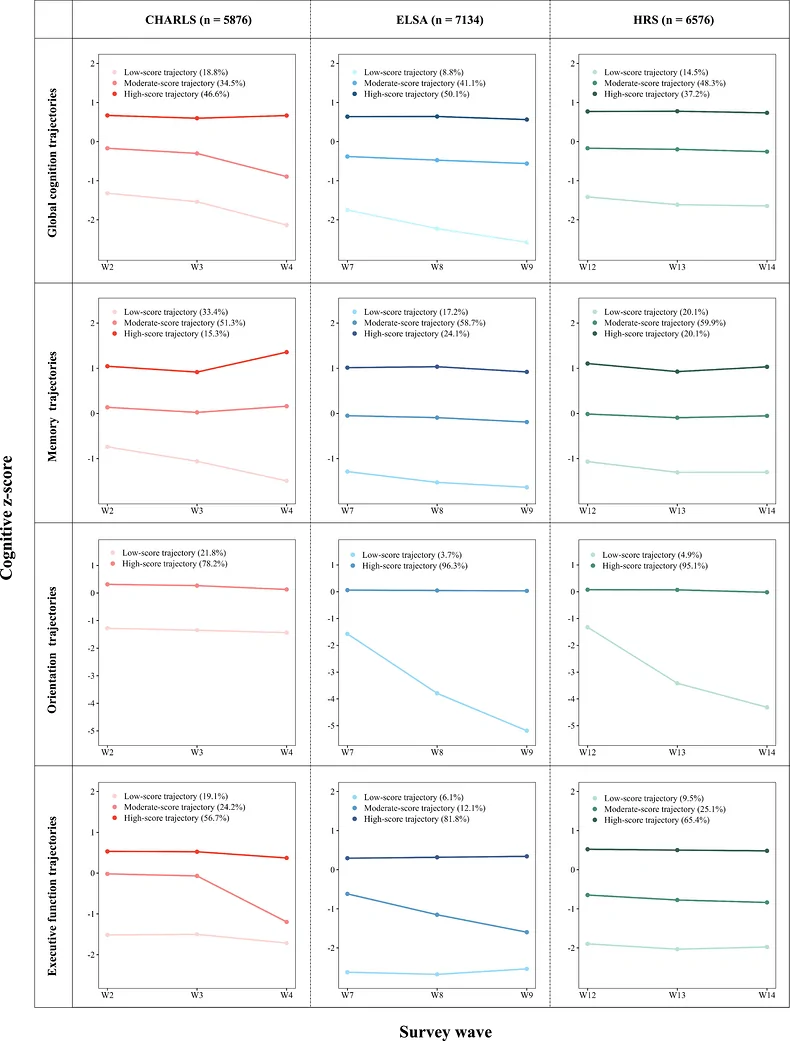
Estimated trajectories of global cognitive function and domain‐specific cognitive function in the CHARLS, ELSA, and HRS cohorts. Cognitive scores were standardized as z‐scores. Within each cognitive domain, the y‐axis scale was standardized across the three cohorts to facilitate visual comparison. Different cognitive domains used different y‐axis ranges because their standardized score distributions differed. Points represent estimated trajectory means at each observed survey wave, and solid line segments connect adjacent survey waves. CHARLS, China Health and Retirement Longitudinal Study; ELSA, English Longitudinal Study of Ageing; HRS, Health and Retirement Study.

To determine the optimal number of trajectories for each cognitive domain, we considered GBTM model iterations, model‐fit indices (Tables S10–S13), and parsimony. Based on these criteria, different numbers of trajectories were identified for each domain (Figure 3). Across the three cohorts, we identified three global cognitive aging trajectories: low‐, moderate‐, and high‐score trajectories.

### 3.4 Associations between baseline frailty status and cognitive aging trajectories

**FIGURE 4:**
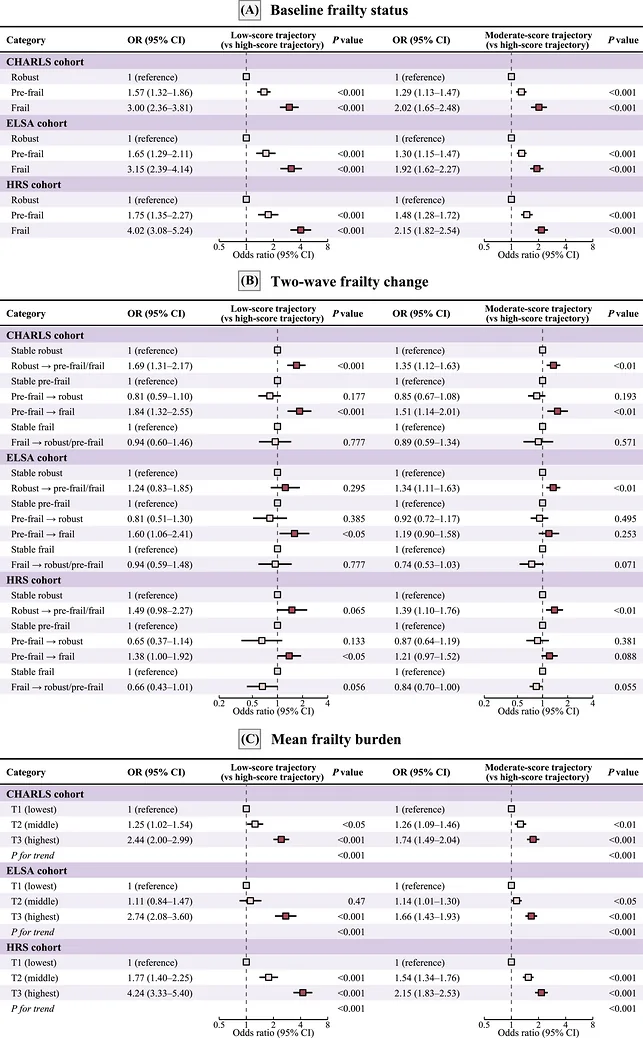
Forest plot for the association between frailty exposures and global cognitive trajectory membership. (A) Baseline frailty status. (B) Two‐wave frailty change. (C) Mean frailty burden. The high‐score trajectory served as the reference outcome category. In panel A, robust participants served as the reference exposure group. In panel B, stable robust, stable pre‐frail, and stable frail participants served as the corresponding reference exposure groups within each preceding frailty‐status stratum. In panel C, the lowest tertile (T1) served as the reference exposure group. Models were adjusted for age, sex, education, marital status, smoking status, drinking status, and body mass index. Square markers represent odds ratios, horizontal lines represent 95% confidence intervals, and the vertical reference line indicates OR = 1. CHARLS, China Health and Retirement Longitudinal Study; CI, confidence interval; ELSA, English Longitudinal Study of Ageing; HRS, Health and Retirement Study; OR, odds ratio.

Figure 4A and Table S14 summarize the associations between baseline frailty status and global cognitive trajectory membership. In the fully adjusted model, frail participants had higher odds of belonging to less‐favorable global cognitive trajectories than robust participants. For the low‐score global cognitive trajectory, the multivariable‐adjusted ORs and 95% CIs were 3.00 (95% CI: 2.36–3.81) in CHARLS, 3.15 (95% CI: 2.39–4.14) in ELSA, and 4.02 (95% CI: 3.08–5.24) in HRS (all *p* < 0.001). For the moderate‐score global cognitive trajectory, the corresponding ORs were 2.02 (95% CI: 1.65–2.48) in CHARLS, 1.92 (95% CI: 1.62–2.27) in ELSA, and 2.15 (95% CI: 1.82–2.54) in HRS (all *p* < 0.001). Pre‐frail participants also had higher odds of belonging to less‐favorable global cognitive trajectory groups than robust participants. Complete domain‐specific results are presented in Table S14 and Figure S3.

### 3.5 Associations between two‐wave frailty change and cognitive aging trajectories

Figure 4B and Table S15 summarize the associations between two‐wave frailty change and global cognitive trajectory membership. In the fully adjusted model, compared with participants who remained robust, those who progressed from robust to pre‐frail or frail status had higher odds of belonging to less‐favorable global cognitive trajectories. For the low‐score global cognitive trajectory, the multivariable‐adjusted ORs were 1.69 (95% CI: 1.31–2.17) in CHARLS, 1.24 (95% CI: 0.83–1.85) in ELSA, and 1.49 (95% CI: 0.98–2.27) in HRS. For the moderate‐score global cognitive trajectory, the corresponding ORs were 1.35 (95% CI: 1.12–1.63) in CHARLS, 1.34 (95% CI: 1.11–1.63) in ELSA, and 1.39 (95% CI: 1.10–1.76) in HRS.

Compared with participants who remained frail, those who recovered from frail to pre‐frail or robust status had lower odds of belonging to less‐favorable global cognitive trajectory groups. For the low‐score global cognitive trajectory, ORs were 0.94 (95% CI: 0.60–1.46) in CHARLS, 0.94 (95% CI: 0.59–1.48) in ELSA, and 0.66 (95% CI: 0.43–1.01) in HRS. For the moderate‐score global cognitive trajectory, the corresponding ORs were 0.89 (95% CI: 0.59–1.34) in CHARLS, 0.74 (95% CI: 0.53–1.03) in ELSA, and 0.84 (95% CI: 0.70–1.00) in HRS. Among participants who were pre‐frail in the preceding survey, recovery to robust status was generally associated with more‐favorable cognitive trajectories, whereas progression to frailty was generally associated with less‐favorable trajectories. Complete domain‐specific results are presented in Table S15 and Figure S4.

### 3.6 Associations between mean frailty burden and cognitive aging trajectories

Figure 4C and Table S16 summarize the associations between mean frailty burden and global cognitive trajectory membership. In the fully adjusted model, participants in the highest tertile (T3) of mean frailty burden had higher odds of belonging to less‐favorable global cognitive trajectory groups than those in the lowest tertile (T1). For the low‐score global cognitive trajectory, the multivariable‐adjusted ORs were 2.44 (95% CI: 2.00–2.99) in CHARLS, 2.74 (95% CI: 2.08–3.60) in ELSA, and 4.24 (95% CI: 3.33–5.40) in HRS (all *p *< 0.001). For the moderate‐score global cognitive trajectory, the corresponding ORs were 1.74 (95% CI: 1.49–2.04) in CHARLS, 1.66 (95% CI: 1.43–1.93) in ELSA, and 2.15 (95% CI: 1.83–2.53) in HRS (all *p* < 0.001). Participants in the middle tertile (T2) also exhibited elevated odds, although with smaller effect sizes. Trend tests revealed increasing odds of less‐favorable global cognitive trajectories across tertiles of mean frailty burden in all three cohorts (all *p‐*values for trend < 0.001). Complete domain‐specific results are presented in Table S16 and Figure S5.

## 4 SENSITIVITY ANALYSES

For the secondary GBTM analyses, results were also generally consistent when frailty status was defined using the alternative FI cutoff scheme (Tables S17–S19). Although some effect estimates were attenuated, the overall associations of frailty measures with cognitive trajectory membership remained after additional adjustment for baseline cognitive z‐scores (Tables S20–S22) and in lagged‐exposure analyses (Tables S23–S24). The findings were also similar after applying inverse probability weighting to account for potential attrition or selection bias (Tables S25–S28), and using modified FIs that separately excluded depression or mental health‐related deficits, disability‐related deficits, and physical functioning or mobility‐related deficits (Tables S29–S37). After FDR correction using the Benjamini–Hochberg method, the overall GBTM findings remained materially unchanged (Tables S38–S40). For the primary LMM analyses, the overall patterns of association for baseline frailty status and two‐wave frailty change were generally consistent when the alternative FI cutoff scheme was applied (Table S41).

## 5 DISCUSSION

In this multicohort study of middle‐aged and older adults from CHARLS, ELSA, and HRS, we examined how baseline frailty status, two‐wave frailty change, and mean frailty burden were associated with annual changes in global and domain‐specific cognitive function and, secondarily, with cognitive trajectory membership. Three main findings emerged from the primary linear mixed‐effects analyses. First, compared with robust participants, those who were pre‐frail or frail at baseline had more negative annual changes in global cognition. Second, worsening from robust to pre‐frail or frail status was associated with more negative annual global cognitive change, whereas changes among participants who were initially pre‐frail or frail showed no clear associations. Third, higher mean frailty burden was associated with more negative annual global cognitive change. In the secondary GBTM analyses, baseline pre‐frailty and frailty, worsening from robust status, and greater mean frailty burden were generally associated with less‐favorable cohort‐relative patterns of global and domain‐specific cognitive aging. These findings suggest that dynamic exposure to frailty may help characterize heterogeneity in cognitive aging.

Cognitive aging is characterized by heterogeneous longitudinal changes in cognitive function. Individuals with similar baseline cognitive scores may experience different subsequent patterns of change, and population‐averaged estimates may not fully capture this variability.^12^, ^13^ Prior trajectory‐based studies have demonstrated that cognitive aging can be described by distinct longitudinal patterns, including relatively stable high‐functioning trajectories and lower‐score or declining trajectories.^14^, ^15^, ^16^ GBTM provides a useful framework for summarizing this heterogeneity through empirical trajectory groups rather than relying solely on mean differences over time.^17^, ^29^ By applying GBTM to repeated cognitive assessments across three aging cohorts, our study extends previous work by linking longitudinal frailty exposure to heterogeneous patterns of global and domain‐specific cognitive aging. Because the GBTM groups in the present study reflected combinations of cohort‐relative cognitive level and longitudinal change, they should not be interpreted as pure decline‐rate classes. The secondary GBTM analyses therefore complemented, rather than replicated, the primary linear mixed‐effects analyses, which directly estimated exposure‐related differences in average rates of cognitive change.^11^

These findings are consistent with a growing body of evidence linking frailty to poorer cognitive outcomes. Cross‐sectional and longitudinal studies have reported that frailty and pre‐frailty are associated with cognitive impairment, lower cognitive performance, and increased risk of dementia.^11^, ^30^, ^31^, ^32^ The observed associations may reflect shared biological and behavioral pathways, including chronic inflammation, oxidative stress, vascular dysfunction, insulin resistance, reduced physical activity, and multisystem physiological dysregulation.^33^, ^34^, ^35^, ^36^ Frailty is increasingly recognized as a dynamic condition rather than a fixed state measured at a single time point.

Prior studies have linked changes in frailty status to cardiovascular disease, disability, mortality, and dementia.^10^, ^22^, ^37^, ^38^ Building on this evidence, our findings suggest that changes in frailty status may offer additional information beyond baseline status alone. Ding et al. reported that frailty progression was associated with an increased risk of incident all‐cause dementia, whereas recovery from pre‐frailty was associated with a reduced dementia risk.^10^ This study complements and extends this evidence using a deficit‐accumulation FI and examining both annual cognitive change and GBTM‐derived multidomain cognitive aging trajectories, rather than a modified Fried frailty phenotype and a binary dementia endpoint. Because dementia is a relatively late clinical endpoint, these cognitive outcomes may capture earlier and more heterogeneous patterns of change.

Several interpretations of these findings warrant consideration. Worsening frailty may reflect increasing multisystem vulnerability, reduced physiological reserve, and a greater accumulation of health deficits over time, which could contribute to more negative cognitive change. Conversely, improvement in frailty status may reflect recovery of physical function or a reduced burden of health deficits. However, because this study was observational, we cannot determine whether changes in frailty status causally affect cognitive decline. Early cognitive changes, neurodegenerative processes, or shared underlying causes may contribute to both frailty and cognitive aging. In the secondary GBTM analyses, additional adjustment for baseline cognitive z‐scores and lagged‐exposure analyses did not materially alter the overall pattern of associations, although residual reverse causality cannot be excluded.

Unlike frailty status at a single time point, mean frailty burden reflects the average burden of health deficits across the preceding and baseline assessments, although this two‐wave measure should not be interpreted as long‐term cumulative exposure. In the primary linear mixed‐effects analyses, the middle and highest tertiles were associated with more negative annual global cognitive change than the lowest tertile, although the pooled association for the highest tertile showed substantial between‐cohort heterogeneity (*I^2^* = 62.1%). In the secondary GBTM analyses, greater mean frailty burden was associated with higher odds of membership in less‐favorable global and domain‐specific cognitive trajectories. These findings suggest that repeated frailty assessments may provide additional information when characterizing cognitive aging, although they do not establish that reducing frailty burden would improve cognitive outcomes.

Several implications for future research warrant consideration. First, repeated frailty assessments may help characterize heterogeneity in cognitive aging by capturing two‐wave changes and mean frailty burden. Second, two‐wave frailty change may help characterize changing patterns of health vulnerability in aging populations. Third, because frailty is potentially modifiable, future longitudinal studies and randomized trials are needed to determine whether modifying frailty alters cognitive outcomes. However, our findings do not establish that frailty improvement preserves cognitive function or justify specific clinical interventions.

This study has several strengths. First, we used harmonized data from three large, nationally representative aging cohorts spanning China, England, and the United States, allowing us to examine the associations between longitudinal frailty exposure and cognitive aging across diverse geographic, cultural, and healthcare contexts. In addition, we reported cohort‐specific estimates and random‐effects pooled estimates and assessed between‐cohort heterogeneity. Second, frailty exposure was characterized using baseline frailty status, two‐wave frailty change, and mean frailty burden, thereby capturing complementary aspects of longitudinal frailty exposure. Third, we examined both global and domain‐specific cognition, including memory, orientation, and executive function, enabling a more detailed characterization of cognitive aging than analyses based on a single cognitive outcome. Fourth, the primary linear mixed‐effects analyses directly estimated exposure‐related differences in annual cognitive change, while the secondary GBTM analyses provided complementary information on cohort‐relative cognitive patterns reflecting both cognitive level and longitudinal change. Finally, we conducted multiple sensitivity analyses, including alternative FI cutoffs, additional adjustment for baseline cognitive z‐scores, lagged‐exposure analyses, inverse probability weighting, and modified FIs excluding potentially overlapping deficit domains.

Several limitations should also be considered. First, although cognitive outcomes were harmonized into comparable domains and standardized within each cohort and domain, assessment instruments differed across CHARLS, ELSA, and HRS. Differences in trajectory‐group proportions therefore may reflect both population differences and cohort‐specific variation in tests, score distributions, language, culture, education, and ceiling or floor effects. Accordingly, low‐, moderate‐, and high‐score trajectories should be interpreted as cohort‐specific relative patterns rather than directly comparable prevalence estimates across cohorts. Second, two‐wave frailty change and mean frailty burden were derived from only two assessments approximately two years apart and therefore could not capture more complex or longer‐term changes in frailty. Third, some two‐wave frailty‐change subgroups had relatively small sample sizes, which may have limited statistical power, particularly for detecting modest associations, and contributed to wider CIs. Fourth, although participants with physician‐diagnosed cognitive disorders were excluded and sensitivity analyses included additional adjustment for baseline cognitive z‐scores in the GBTM models and lagged‐exposure analyses, preclinical neurodegenerative changes may still have contributed to both frailty and subsequent cognitive aging. Fifth, the FI was constructed using harmonized deficits available across cohorts and demonstrated acceptable internal consistency; however, using fewer than 30 deficits may have limited measurement granularity. Additionally, the FI included deficits related to depression, disability, and physical functioning or mobility limitations, which may overlap with early cognitive pathology or prodromal neurodegenerative processes. We addressed this concern through sensitivity analyses using modified FIs that excluded these domains separately, but residual overlap cannot be fully ruled out. Sixth, although we compared baseline characteristics between included and excluded participants and used inverse probability weighting to address potential selection bias, residual bias due to mortality, informative dropout, or unmeasured factors associated with missing repeated cognitive assessments cannot be fully excluded. Seventh, although we adjusted for multiple demographic and lifestyle factors, residual confounding from unmeasured factors, such as genetic susceptibility, diet, medication use, socioeconomic conditions, and other health behaviors, may remain. Finally, because this study was observational, the findings should not be interpreted as evidence that improving frailty status would causally alter cognitive outcomes.

In this multicohort study, baseline pre‐frailty and frailty, worsening from robust status, and higher mean frailty burden were associated with more negative annual global cognitive change. Secondary GBTM analyses complemented the primary findings by showing that these frailty exposures were generally associated with less‐favorable cohort‐relative patterns of global and domain‐specific cognitive aging, reflecting both cognitive level and longitudinal change. Together, these findings highlight the relevance of repeated frailty assessments for characterizing heterogeneity in cognitive aging. Further longitudinal studies are needed to examine longer‐term frailty changes and underlying pathways, and randomized trials are needed to determine whether modifying frailty alters cognitive outcomes.

## AUTHOR CONTRIBUTIONS

**Xingjie Wang**: Methodology; study design; data collection; formal analysis; visualization; writing—original draft. **Hongtao Hao**: Data collection; formal analysis; writing—original draft. **Tancheng Jiang**: Methodology; Writing—original draft. **Xutong Yao**: Writing—review and editing. **Yusheng Duan**: Writing—review and editing. **Junmei Xia**: Writing—review and editing. **Cenyi Wang**: Methodology; formal analysis; validation; writing—review and editing; supervision. **Jiling Liang** Methodology; writing—review and editing; supervision; project administration.

## CONFLICT OF INTEREST STATEMENT

The authors declare no competing interests. Author disclosures are available in the Supporting Information.

## CONSENT STATEMENT

The CHARLS received approval from the Ethical Review Committee of Peking University (IRB00001052‐11015). The ELSA was approved by the London Multicentre Research Ethics Committee (MREC/01/2/91). The HRS was approved by the Institutional Review Board at the University of Michigan and the National Institute on Aging (HUM00061128). All participants gave signed informed consent at the time of participation.

## Supporting information



Supporting Information

Supporting Information
