# Supplementary material for: Dynamic frailty exposure and cognitive aging trajectories: A multicohort study
Source: Alzheimers Dement. 2026 Sep 10;22(9):e71821. doi: 10.1002/alz.71821 (PMC13562614; doi:10.1002/alz.71821)
Supplement: Supplementary file 2 [file ALZ-22-e71821-s002.docx]

**Additional file**

# Supplementary Tables

**Table S1.** Definitions and exclusion criteria for cognitive disorders

| **Cohort** | **Items** | **Exclusion criterion** |
| --- | --- | --- |
| CHARLS | Doctor diagnosed:  Ever had memory problems | Participants who answered “yes” to any question will be excluded. |
| ELSA | Doctor diagnosed:  Ever had Alzheimer's  Ever had dementia  Ever had memory problems | Participants who answered “yes” to any question will be excluded. |
| HRS | Doctor diagnosed:  Ever had memory problems  Ever reported Alzheimer's  Ever reported dementia | Participants who answered “yes” to any question will be excluded. |

**Abbreviations:** CHARLS, China Health and Retirement Longitudinal Study; ELSA, English Longitudinal Study of Ageing; HRS, Health and Retirement Study.

**Table S2.** Frailty-index items, cut-off values, deficit domains, and exclusion rules for modified frailty indices

| **No.** | **Description of the item** | | | **Cut-off value** | **Deficit domain** | **Excluded in modified FI** |
| --- | --- | --- | --- | --- | --- | --- |
|  | **CHARLS** | **ELSA** | **HRS** |  |  |  |
| 1 | Self-reported physician-diagnosed hypertension | | | Yes = 1, No = 0 | Chronic disease | Not excluded |
| 2 | Self-reported physician-diagnosed diabetes | | | Yes = 1, No = 0 | Chronic disease | Not excluded |
| 3 | Self-reported physician-diagnosed cancer | | | Yes = 1, No = 0 | Chronic disease | Not excluded |
| 4 | Self-reported physician-diagnosed arthritis | | | Yes = 1, No = 0 | Chronic disease | Not excluded |
| 5 | Self-reported physician-diagnosed chronic lung disease | | | Yes = 1, No = 0 | Chronic disease | Not excluded |
| 6 | Self-reported physician-diagnosed any emotional, nervous, or psychiatric problems | | | Yes = 1, No = 0 | Chronic disease | Excluded from modified FI excluding depression/mental health-related deficits |
| 7 | Self-reported physician-diagnosed heart disease | | | Yes = 1, No = 0 | Chronic disease | Not excluded |
| 8 | Self-reported physician-diagnosed stroke | | | Yes = 1, No = 0 | Chronic disease | Not excluded |
| 9 | Self-reported vision problems | Self-reported eyesight (while using lenses if appropriate) | | Yes = 1, No = 0 in the CHARLS; Poor or fair = 1, excellent, very good, or good = 0 in the ELSA and HRS | General health-related symptom | Not excluded |
| 10 | Self-reported hearing problems | Self-reported hearing (while using hearing aid if appropriate) | | Yes = 1, No = 0 in the CHARLS; Poor or fair = 1, excellent, very good, or good = 0 in the ELSA and HRS | General health-related symptom | Not excluded |
| 11 | Self-reported general health status | | | Very poor or poor = 1, very good, good, or fair = 0 | General health-related symptom | Not excluded |
| 12 | Difficulty with dressing | | | Yes = 1, No = 0 | Disability-related deficit: ADL limitation | Excluded from modified FI excluding disability-related deficits |
| 13 | Difficulty with bathing or showering | | | Yes = 1, No = 0 | Disability-related deficit: ADL limitation | Excluded from modified FI excluding disability-related deficits |
| 14 | Difficulty with eating | | | Yes = 1, No = 0 | Disability-related deficit: ADL limitation | Excluded from modified FI excluding disability-related deficits |
| 15 | Difficulty with getting in and out of bed | | | Yes = 1, No = 0 | Disability-related deficit: ADL limitation | Excluded from modified FI excluding disability-related deficits |
| 16 | Difficulty with using the toilet | | | Yes = 1, No = 0 | Disability-related deficit: ADL limitation | Excluded from modified FI excluding disability-related deficits |
| 17 | Difficulty with managing money | | | Yes = 1, No = 0 | Disability-related deficit: IADL limitation | Excluded from modified FI excluding disability-related deficits |
| 18 | Difficulty with taking medication | | | Yes = 1, No = 0 | Disability-related deficit: IADL limitation | Excluded from modified FI excluding disability-related deficits |
| 19 | Difficulty with shopping for groceries | | | Yes = 1, No = 0 | Disability-related deficit: IADL limitation | Excluded from modified FI excluding disability-related deficits |
| 20 | Difficulty with preparing meals | | | Yes = 1, No = 0 | Disability-related deficit: IADL limitation | Excluded from modified FI excluding disability-related deficits |
| 21 | Mobility: difficulty with walking 100 yards or one block | | | Yes = 1, No = 0 | Physical functioning/mobility-related deficit | Excluded from modified FI excluding physical functioning/mobility-related deficits |
| 22 | Mobility: difficulty with getting up from a chair after sitting for long periods | | | Yes = 1, No = 0 | Physical functioning/mobility-related deficit | Excluded from modified FI excluding physical functioning/mobility-related deficits |
| 23 | Mobility: difficulty with climbing several flights of stairs without resting | | | Yes = 1, No = 0 | Physical functioning/mobility-related deficit | Excluded from modified FI excluding physical functioning/mobility-related deficits |
| 24 | Mobility: difficulty with lifting or carrying weights over 10 pounds/jins | | | Yes = 1, No = 0 | Physical functioning/mobility-related deficit | Excluded from modified FI excluding physical functioning/mobility-related deficits |
| 25 | Mobility: difficulty with picking up a coin from the table | | | Yes = 1, No = 0 | Physical functioning/mobility-related deficit | Excluded from modified FI excluding physical functioning/mobility-related deficits |
| 26 | Mobility: difficulty with stooping, kneeling, or crouching | | | Yes = 1, No = 0 | Physical functioning/mobility-related deficit | Excluded from modified FI excluding physical functioning/mobility-related deficits |
| 27 | Mobility: difficulty with reaching arms above shoulder level | | | Yes = 1, No = 0 | Physical functioning/mobility-related deficit | Excluded from modified FI excluding physical functioning/mobility-related deficits |
| 28 | Depression: CESD-10 questionnaire | Depression: CESD-8 questionnaire | | CESD-10 > 10 = 1, ≤ 10 = 0 in the CHARLS; CESD-8 ≥ 4 = 1, < 4 = 0 in the ELSA and HRS | Depression/mental health-related deficit | Excluded from modified FI excluding depression/mental health-related deficits |

**Notes.** Depression was assessed by the Center for Epidemiologic Studies Depression Scale (CESD). In CHARLS, CESD-10 was used, and the total score ranged from 0 to 30. In ELSA and HRS, CESD-8 was used, and the total score ranged from 0 to 8. A higher score indicated more severe depressive symptoms. The original FI included 28 non-cognitive deficits across chronic diseases, sensory and general health-related symptoms, ADL/IADL disability, physical functioning/mobility limitations, and depression/mental health-related deficits. Memory-related disease diagnoses and cognitive test items were not included in the FI. For sensitivity analyses, modified FIs were recalculated by separately excluding depression/mental health-related deficits, disability-related deficits, and physical functioning/mobility-related deficits. Depression/mental health-related deficits included items 6 and 28; disability-related deficits included ADL/IADL limitations, items 12–20; and physical functioning/mobility-related deficits included items 21–27. The same FI cut-off points were applied to define frailty status for the original and modified FIs: robust, FI ≤ 0.10; pre-frail, 0.10 < FI < 0.25; and frail, FI ≥ 0.25.

**Abbreviations:** CHARLS, China Health and Retirement Longitudinal Study; ELSA, English Longitudinal Study of Ageing; HRS, Health and Retirement Study; FI, frailty index; ADL, activities of daily living; IADL, instrumental activities of daily living; CESD, Center for Epidemiologic Studies Depression Scale.

****Table** S3**.** Internal consistency of the 28-item frailty index in each cohort**

| **Cohort** | **Analytic sample, n** | **Number of FI items** | **Raw Cronbach’s alpha** | **Standardized Cronbach’s alpha** |
| --- | --- | --- | --- | --- |
| CHARLS | 5876 | 28 | 0.762 | 0.779 |
| ELSA | 7134 | 28 | 0.826 | 0.842 |
| HRS | 6576 | 28 | 0.794 | 0.821 |

**Note.** Cronbach’s alpha was calculated using the individual deficit variables included in the 28-item frailty index (FI) within each cohort. A Cronbach’s alpha value ≥ 0.70 was considered to indicate acceptable internal consistency. Because the FI is a multidimensional deficit-accumulation index rather than a unidimensional psychometric scale, Cronbach’s alpha was interpreted as a descriptive indicator of item coherence rather than as the sole criterion for FI validity.

**Abbreviations:** CHARLS, China Health and Retirement Longitudinal Study; ELSA, English Longitudinal Study of Ageing; FI, frailty index; HRS, Health and Retirement Study.

**Table S4.** Measurement of cognitive function in CHARLS, ELSA, and HRS

|  | **Measurement** | | |
| --- | --- | --- | --- |
|  | **CHARLS** | **ELSA** | **HRS** |
| Memory (scores: 0–20) | **Immediate and delayed word recall (scores: 0**–**20):** The participants were read a set of 10 words and were asked to recall the words as many as they could, in any order (Immediate recall). After conducting some other tests, the participants were again asked to recall the words they remembered (Delayed recall). | | |
| Orientation (scores: 0–4) | **Orientation (scores: 0**–**4):** The participants were asked to tell today’s date (year, month, day, and day of the week). | | |
| Executive function (scores: 0–7) | **Serial 7s subtraction test (scores: 0**–**5):** Participants were asked to subtract 7 serially from 100 five times. | | |
|  | **Visuospatial ability (scores: 0**–**2):** Participants were asked to copy a figure of two overlapping pentagons. | **Backward counting (scores: 0**–**2):** The participants were asked to count backwards for 10 continuous numbers as quickly as possible starting with the number 20. Two points were given for success in the first try, one point for success in the second try, and 0 for unsuccessful counting in both tries. | |

**Abbreviations:** CHARLS, China Health and Retirement Longitudinal Study; ELSA, English Longitudinal Study of Ageing; HRS, Health and Retirement Study.

**Table S5.** Number and percentage of missing covariate values for baseline frailty status analyses

| **Characteristic** | **CHARLS** | | | |  | **ELSA** | | | |  | **HRS** | | | |
| --- | --- | --- | --- | --- | --- | --- | --- | --- | --- | --- | --- | --- | --- | --- |
|  | **Robust**  **(n = 2757)** | **Pre-frail**  **(n = 2328)** | **Frail**  **(n = 791)** | **All**  **(n = 5876)** |  | **Robust**  **(n = 3496)** | **Pre-frail**  **(n = 2234)** | **Frail**  **(n = 1404)** | **All**  **(n = 7134)** |  | **Robust**  **(n = 1593)** | **Pre-frail**  **(n = 2719)** | **Frail**  **(n = 2264)** | **All**  **(n = 6576)** |
| **Age, n (%)** | 0 (0.00%) | 0 (0.00%) | 0 (0.00%) | 0 (0.00%) |  | 0 (0.00%) | 0 (0.00%) | 0 (0.00%) | 0 (0.00%) |  | 0 (0.00%) | 0 (0.00%) | 0 (0.00%) | 0 (0.00%) |
| **Sex, n (%)** | 0 (0.00%) | 0 (0.00%) | 0 (0.00%) | 0 (0.00%) |  | 0 (0.00%) | 0 (0.00%) | 0 (0.00%) | 0 (0.00%) |  | 0 (0.00%) | 0 (0.00%) | 0 (0.00%) | 0 (0.00%) |
| **Marital status, n (%)** | 2 (0.07%) | 0 (0.00%) | 2 (0.25%) | 4 (0.07%) |  | 1 (0.03%) | 0 (0.00%) | 0 (0.00%) | 1 (0.01%) |  | 0 (0.00%) | 0 (0.00%) | 0 (0.00%) | 0 (0.00%) |
| **Education, n (%)** | 0 (0.00%) | 0 (0.00%) | 0 (0.00%) | 0 (0.00%) |  | 220 (6.29%) | 185 (8.28%) | 126 (8.97%) | 531 (7.44%) |  | 1 (0.06%) | 1 (0.04%) | 1 (0.04%) | 3 (0.05%) |
| **Smoking status, n (%)** | 0 (0.00%) | 0 (0.00%) | 0 (0.00%) | 0 (0.00%) |  | 0 (0.00%) | 0 (0.00%) | 1 (0.07%) | 1 (0.01%) |  | 11 (0.69%) | 18 (0.66%) | 18 (0.80%) | 47 (0.71%) |
| **Drinking status, n (%)** | 2 (0.07%) | 2 (0.09%) | 2 (0.25%) | 6 (0.10%) |  | 254 (7.27%) | 171 (7.65%) | 198 (14.10%) | 623 (8.73%) |  | 0 (0.00%) | 0 (0.00%) | 0 (0.00%) | 0 (0.00%) |
| **BMI, n (%)** | 571  (20.71%) | 471 (20.23%) | 152 (19.22%) | 1194 (20.32%) |  | 624 (17.85%) | 306 (13.70%) | 270 (19.23%) | 1200 (16.82%) |  | 218 (13.68%) | 358 (13.17%) | 458 (20.23%) | 1034 (15.72%) |

**Abbreviations:** CHARLS, China Health and Retirement Longitudinal Study; ELSA, English Longitudinal Study of Ageing; HRS, Health and Retirement Study; BMI, body mass index.

**Table S6.** Baseline characteristics of participants for baseline frailty status analyses using unimputed data

| **Characteristic** | **CHARLS (n = 5876)** | | |  | **ELSA (n = 7134)** | | |  | **HRS (n = 6576)** | | |
| --- | --- | --- | --- | --- | --- | --- | --- | --- | --- | --- | --- |
|  | **Robust** | **Pre-frail** | **Frail** |  | **Robust** | **Pre-frail** | **Frail** |  | **Robust** | **Pre-frail** | **Frail** |
| **Number (%)** | 2757 (46.9%) | 2328 (39.6%) | 791 (13.5%) |  | 3496 (49.0%) | 2234 (31.3%) | 1404 (19.7%) |  | 1593 (24.2%) | 2719 (41.3%) | 2264 (34.4%) |
| **Age, mean (SD), years** | 57.9 (7.8) | 59.8 (8.2) | 62.2 (8.4) |  | 65.0 (7.9) | 69.2 (8.5) | 71.4 (9.6) |  | 71.1 (6.3) | 73.2 (6.6) | 74.2 (7.5) |
| **Sex, n (%)** |  |  |  |  |  |  |  |  |  |  |  |
| Female | 963 (34.9%) | 1082 (46.5%) | 457 (57.8%) |  | 1815 (51.9%) | 1290 (57.7%) | 911 (64.9%) |  | 903 (56.7%) | 1549 (57.0%) | 1521 (67.2%) |
| Male | 1794 (65.1%) | 1246 (53.5%) | 334 (42.2%) |  | 1681 (48.1%) | 944 (42.3%) | 493 (35.1%) |  | 690 (43.3%) | 1170 (43.0%) | 743 (32.8%) |
| **Marital status, n (%)** |  |  |  |  |  |  |  |  |  |  |  |
| Married or partnered | 2539 (92.2%) | 2110 (90.6%) | 658 (83.4%) |  | 2560 (73.2%) | 1472 (65.9%) | 755 (53.8%) |  | 1037 (65.1%) | 1628 (59.9%) | 1076 (47.5%) |
| Other marital status | 216 (7.8%) | 218 (9.4%) | 131 (16.6%) |  | 935 (26.8%) | 762 (34.1%) | 649 (46.2%) |  | 556 (34.9%) | 1091 (40.1%) | 1188 (52.5%) |
| **Education, n (%)** |  |  |  |  |  |  |  |  |  |  |  |
| Below high school | 2264 (82.1%) | 2048 (88.0%) | 739 (93.4%) |  | 544 (16.6%) | 572 (27.9%) | 573 (44.8%) |  | 132 (8.3%) | 343 (12.6%) | 556 (24.6%) |
| College or above | 69 (2.5%) | 33 (1.4%) | 8 (1.0%) |  | 912 (27.8%) | 389 (19.0%) | 131 (10.3%) |  | 586 (36.8%) | 745 (27.4%) | 316 (14.0%) |
| High school | 424 (15.4%) | 247 (10.6%) | 44 (5.6%) |  | 1820 (55.6%) | 1088 (53.1%) | 574 (44.9%) |  | 874 (54.9%) | 1630 (60.0%) | 1391 (61.5%) |
| **Smoking status, n (%)** |  |  |  |  |  |  |  |  |  |  |  |
| Ever smokers | 1510 (54.8%) | 1138 (48.9%) | 310 (39.2%) |  | 1982 (56.7%) | 1446 (64.7%) | 990 (70.6%) |  | 784 (49.6%) | 1499 (55.5%) | 1294 (57.6%) |
| Never smokers | 1247 (45.2%) | 1190 (51.1%) | 481 (60.8%) |  | 1514 (43.3%) | 788 (35.3%) | 413 (29.4%) |  | 798 (50.4%) | 1202 (44.5%) | 952 (42.4%) |
| **Drinking status, n (%)** |  |  |  |  |  |  |  |  |  |  |  |
| Ever drinkers | 1683 (61.1%) | 1275 (54.8%) | 391 (49.6%) |  | 3000 (92.5%) | 1819 (88.2%) | 875 (72.6%) |  | 1036 (65.0%) | 1542 (56.7%) | 945 (41.7%) |
| Never drinkers | 1072 (38.9%) | 1051 (45.2%) | 398 (50.4%) |  | 242 (7.5%) | 244 (11.8%) | 331 (27.4%) |  | 557 (35.0%) | 1177 (43.3%) | 1319 (58.3%) |
| **BMI, mean (SD), kg/m^2^** | 23.5 (3.5) | 24.1 (3.8) | 24.3 (3.9) |  | 27.1 (4.3) | 28.7 (5.2) | 31.1 (6.2) |  | 27.4 (4.6) | 29.8 (5.7) | 31.2 (6.4) |
| **Cognitive scores, mean (SD)** |  |  |  |  |  |  |  |  |  |  |  |
| Memory | 8.0 (3.4) | 7.4 (3.3) | 6.5 (3.2) |  | 11.7 (3.3) | 10.6 (3.5) | 9.4 (3.5) |  | 10.8 (3.3) | 9.9 (3.2) | 8.9 (3.2) |
| Orientation | 3.3 (1.0) | 3.2 (1.1) | 2.9 (1.2) |  | 3.9 (0.4) | 3.8 (0.4) | 3.7 (0.6) |  | 3.8 (0.4) | 3.8 (0.5) | 3.7 (0.6) |
| Executive function | 5.2 (2.0) | 4.8 (2.2) | 4.2 (2.4) |  | 6.3 (1.3) | 6.1 (1.4) | 5.6 (1.9) |  | 5.9 (1.6) | 5.6 (1.7) | 5.0 (2.0) |
| Global | 16.4 (5.1) | 15.2 (5.2) | 13.2 (5.3) |  | 21.8 (3.9) | 20.5 (4.3) | 18.7 (4.7) |  | 19.9 (4.3) | 18.9 (4.3) | 17.1 (4.6) |

**Abbreviations:** CHARLS, China Health and Retirement Longitudinal Study; ELSA, English Longitudinal Study of Ageing; HRS, Health and Retirement Study; BMI, body mass index.

**Table S7.** Association between baseline frailty status and annual change in cognitive z-scores across three longitudinal cohorts

| **Baseline frailty status** | | **CHARLS** |  | **ELSA** |  | **HRS** |  | **Pooled analysis** | | |
| --- | --- | --- | --- | --- | --- | --- | --- | --- | --- | --- |
|  |  | **β (95% CI)** |  | **β (95% CI)** |  | **β (95% CI)** |  | **β (95% CI)** | **I² (%)** | ***P* value** |
| **Global cognition** | |  |  |  |  |  |  |  |  |  |
|  | Robust | reference |  | reference |  | reference |  | reference |  |  |
|  | Pre-frail | -0.020 (-0.030, -0.009) |  | -0.023 (-0.035, -0.011) |  | -0.028 (-0.042, -0.013) |  | -0.023 (-0.030, -0.016) | 0.0 | <0.001 |
|  | Frail | -0.026 (-0.042, -0.011) |  | -0.027 (-0.042, -0.013) |  | -0.014 (-0.029, 0.001) |  | -0.023 (-0.031, -0.014) | 0.0 | <0.001 |
| **Memory** | |  |  |  |  |  |  |  |  |  |
|  | Robust | reference |  | reference |  | reference |  | reference |  |  |
|  | Pre-frail | -0.020 (-0.034, -0.007) |  | -0.015 (-0.027, -0.002) |  | -0.013 (-0.028, 0.003) |  | -0.016 (-0.024, -0.008) | 0.0 | <0.001 |
|  | Frail | -0.033 (-0.052, -0.013) |  | -0.015 (-0.029, 0.000) |  | -0.005 (-0.021, 0.012) |  | -0.017 (-0.032, -0.002) | 58.9 | 0.031 |
| **Orientation** | |  |  |  |  |  |  |  |  |  |
|  | Robust | reference |  | reference |  | reference |  | reference |  |  |
|  | Pre-frail | 0.003 (-0.009, 0.016) |  | -0.019 (-0.039, 0.000) |  | -0.036 (-0.061, -0.010) |  | -0.015 (-0.037, 0.007) | 76.3 | 0.183 |
|  | Frail | 0.011 (-0.007, 0.029) |  | -0.041 (-0.064, -0.018) |  | -0.044 (-0.071, -0.017) |  | -0.024 (-0.060, 0.012) | 87.0 | 0.196 |
| **Executive function** | |  |  |  |  |  |  |  |  |  |
|  | Robust | reference |  | reference |  | reference |  | reference |  |  |
|  | Pre-frail | -0.006 (-0.017, 0.005) |  | -0.026 (-0.039, -0.012) |  | -0.013 (-0.026, 0.001) |  | -0.014 (-0.026, -0.003) | 59.9 | 0.016 |
|  | Frail | -0.005 (-0.021, 0.011) |  | -0.023 (-0.039, -0.007) |  | -0.022 (-0.036, -0.008) |  | -0.017 (-0.028, -0.005) | 39.4 | 0.004 |

**Notes.** Linear mixed-effects models were fitted using repeated cognitive z-scores as continuous outcomes. Baseline frailty status was defined using the frailty index (FI) as follows: robust, FI ≤ 0.10; pre-frail, 0.10 < FI < 0.25; and frail, FI ≥ 0.25. Robust participants served as the reference group. β coefficients represent the difference in annual change in cognitive z-score compared with the robust group. Models included time, baseline frailty status, and the interaction between time and baseline frailty status, and were adjusted for age, sex, education, marital status, smoking status, drinking status, and body mass index.

**Abbreviations:** BMI, body mass index; CI, confidence interval; CHARLS, China Health and Retirement Longitudinal Study; ELSA, English Longitudinal Study of Ageing; FI, frailty index; HRS, Health and Retirement Study.

**Table S8.** Association between two-wave frailty change and annual change in cognitive z-scores across three longitudinal cohorts

| **Two-wave frailty change** | | **CHARLS** |  | **ELSA** |  | **HRS** |  | **Pooled analysis** | | |
| --- | --- | --- | --- | --- | --- | --- | --- | --- | --- | --- |
|  |  | **β (95% CI)** |  | **β (95% CI)** |  | **β (95% CI)** |  | **β (95% CI)** | **I² (%)** | ***P* value** |
| **Global cognition** | |  |  |  |  |  |  |  |  |  |
|  | Stable robust | reference |  | reference |  | reference |  | reference |  |  |
|  | Robust to pre-frail/frail | -0.031 (-0.046, -0.015) |  | -0.022 (-0.041, -0.002) |  | -0.014 (-0.038, 0.010) |  | -0.025 (-0.035, -0.014) | 0.0 | <0.001 |
|  | Stable pre-frail | reference |  | reference |  | reference |  | reference |  |  |
|  | Pre-frail to robust | 0.005 (-0.014, 0.025) |  | 0.004 (-0.020, 0.029) |  | 0.036 (0.005, 0.068) |  | 0.012 (-0.004, 0.029) | 27.4 | 0.151 |
|  | Pre-frail to frail | -0.004 (-0.035, 0.028) |  | -0.015 (-0.047, 0.016) |  | -0.005 (-0.035, 0.025) |  | -0.008 (-0.026, 0.010) | 0.0 | 0.383 |
|  | Stable frail | reference |  | reference |  | reference |  | reference |  |  |
|  | Frail to robust/pre-frail | -0.008 (-0.030, 0.014) |  | 0.003 (-0.024, 0.030) |  | 0.019 (-0.003, 0.041) |  | 0.005 (-0.012, 0.022) | 36.4 | 0.588 |
| **Memory** | |  |  |  |  |  |  |  |  |  |
|  | Stable robust | reference |  | reference |  | reference |  | reference |  |  |
|  | Robust to pre-frail/frail | -0.031 (-0.050, -0.012) |  | -0.022 (-0.041, -0.002) |  | 0.000 (-0.026, 0.026) |  | -0.020 (-0.036, -0.004) | 42.5 | 0.017 |
|  | Stable pre-frail | reference |  | reference |  | reference |  | reference |  |  |
|  | Pre-frail to robust | 0.002 (-0.022, 0.027) |  | -0.004 (-0.029, 0.020) |  | 0.022 (-0.012, 0.056) |  | 0.004 (-0.012, 0.019) | 0.0 | 0.641 |
|  | Pre-frail to frail | -0.002 (-0.029, 0.026) |  | -0.004 (-0.031, 0.024) |  | 0.020 (-0.004, 0.044) |  | 0.006 (-0.010, 0.022) | 10.9 | 0.451 |
|  | Stable frail | reference |  | reference |  | reference |  | reference |  |  |
|  | Frail to robust/pre-frail | 0.014 (-0.026, 0.053) |  | -0.008 (-0.039, 0.024) |  | 0.003 (-0.030, 0.035) |  | 0.007 (-0.012, 0.027) | 0.0 | 0.468 |
| **Orientation** | |  |  |  |  |  |  |  |  |  |
|  | Stable robust | reference |  | reference |  | reference |  | reference |  |  |
|  | Robust to pre-frail/frail | -0.004 (-0.022, 0.014) |  | 0.012 (-0.019, 0.043) |  | -0.016 (-0.059, 0.027) |  | -0.002 (-0.016, 0.013) | 0.0 | 0.807 |
|  | Stable pre-frail | reference |  | reference |  | reference |  | reference |  |  |
|  | Pre-frail to robust | 0.010 (-0.013, 0.033) |  | 0.014 (-0.025, 0.053) |  | 0.025 (-0.032, 0.081) |  | 0.013 (-0.006, 0.031) | 0.0 | 0.188 |
|  | Pre-frail to frail | 0.000 (-0.026, 0.026) |  | -0.035 (-0.079, 0.008) |  | -0.009 (-0.046, 0.029) |  | -0.009 (-0.028, 0.010) | 0.0 | 0.349 |
|  | Stable frail | reference |  | reference |  | reference |  | reference |  |  |
|  | Frail to robust/pre-frail | -0.023 (-0.059, 0.015) |  | -0.039 (-0.089, 0.011) |  | -0.015 (-0.068, 0.038) |  | -0.025 (-0.051, 0.001) | 0.0 | 0.062 |
| **Executive** | |  |  |  |  |  |  |  |  |  |
|  | Stable robust | reference |  | reference |  | reference |  | reference |  |  |
|  | Robust to pre-frail/frail | -0.019 (-0.035, -0.004) |  | -0.015 (-0.037, 0.006) |  | -0.005 (-0.028, 0.017) |  | -0.015 (-0.026, -0.004) | 0.0 | 0.009 |
|  | Stable pre-frail | reference |  | reference |  | reference |  | reference |  |  |
|  | Pre-frail to robust | 0.000 (-0.021, 0.020) |  | 0.016 (-0.012, 0.043) |  | 0.040 (0.010, 0.069) |  | 0.017 (-0.006, 0.040) | 58.1 | 0.151 |
|  | Pre-frail to frail | -0.006 (-0.029, 0.017) |  | 0.033 (0.002, 0.063) |  | -0.005 (-0.025, 0.016) |  | 0.005 (-0.017, 0.028) | 60.8 | 0.651 |
|  | Stable frail | reference |  | reference |  | reference |  | reference |  |  |
|  | Frail to robust/pre-frail | 0.003 (-0.030, 0.037) |  | -0.022 (-0.057, 0.013) |  | 0.004 (-0.024, 0.032) |  | -0.003 (-0.022, 0.015) | 0.0 | 0.714 |

**Notes.** Linear mixed-effects models were fitted using repeated cognitive z-scores as continuous outcomes. Two-wave frailty change was defined from the preceding survey to the baseline survey. Stable robust, stable pre-frail, and stable frail participants served as the corresponding reference groups within each preceding frailty-status stratum. β coefficients represent the difference in annual change in cognitive z-score compared with the corresponding stable reference group. Models included time, two-wave frailty change, and the interaction between time and two-wave frailty change, and were adjusted for age, sex, education, marital status, smoking status, drinking status, and body mass index.

**Abbreviations:** BMI, body mass index; CI, confidence interval; CHARLS, China Health and Retirement Longitudinal Study; ELSA, English Longitudinal Study of Ageing; FI, frailty index; HRS, Health and Retirement Study.

**Table S9.** Association between the mean frailty burden and annual change in cognitive z-scores across three longitudinal cohorts

| **Mean frailty burden** | | **CHARLS** |  | **ELSA** |  | **HRS** |  | **Pooled analysis** | | |
| --- | --- | --- | --- | --- | --- | --- | --- | --- | --- | --- |
|  |  | **β (95% CI)** |  | **β (95% CI)** |  | **β (95% CI)** |  | **β (95% CI)** | **I² (%)** | ***P* value** |
| **Global cognition** | |  |  |  |  |  |  |  |  |  |
|  | T1 | reference |  | reference |  | reference |  | reference |  |  |
|  | T2 | -0.011 (-0.023, 0.001) |  | -0.016 (-0.028, -0.003) |  | -0.023 (-0.036, -0.009) |  | -0.016 (-0.023, -0.009) | 0.0 | <0.001 |
|  | T3 | -0.025 (-0.037, -0.013) |  | -0.033 (-0.046, -0.020) |  | -0.011 (-0.025, 0.003) |  | -0.023 (-0.036, -0.011) | 62.1 | <0.001 |
| **Memory** | |  |  |  |  |  |  |  |  |  |
|  | T1 | reference |  | reference |  | reference |  | reference |  |  |
|  | T2 | -0.008 (-0.023, 0.007) |  | -0.012 (-0.025, 0.001) |  | -0.011 (-0.025, 0.004) |  | -0.011 (-0.019, -0.002) | 0.0 | 0.012 |
|  | T3 | -0.029 (-0.044, -0.014) |  | -0.021 (-0.034, -0.008) |  | -0.005 (-0.021, 0.010) |  | -0.019 (-0.032, -0.005) | 59.8 | 0.006 |
| **Orientation** | |  |  |  |  |  |  |  |  |  |
|  | T1 | reference |  | reference |  | reference |  | reference |  |  |
|  | T2 | -0.012 (-0.026, 0.002) |  | -0.017 (-0.037, 0.004) |  | -0.041 (-0.066, -0.017) |  | -0.021 (-0.037, -0.005) | 54.4 | 0.011 |
|  | T3 | 0.007 (-0.007, 0.021) |  | -0.044 (-0.065, -0.024) |  | -0.052 (-0.077, -0.026) |  | -0.029 (-0.065, 0.008) | 90.5 | 0.131 |
| **Executive function** | |  |  |  |  |  |  |  |  |  |
|  | T1 | reference |  | reference |  | reference |  | reference |  |  |
|  | T2 | -0.010 (-0.023, 0.002) |  | -0.013 (-0.027, 0.001) |  | -0.014 (-0.027, -0.001) |  | -0.012 (-0.020, -0.005) | 0.0 | 0.001 |
|  | T3 | -0.003 (-0.016, 0.009) |  | -0.030 (-0.044, -0.015) |  | -0.022 (-0.036, -0.009) |  | -0.018 (-0.034, -0.002) | 75.1 | 0.023 |

**Notes.** Linear mixed-effects models were fitted using repeated cognitive z-scores as continuous outcomes. The mean frailty burden was calculated as the mean of the FI measured at the preceding survey and the baseline survey. Participants were categorized into cohort-specific tertiles of the mean frailty burden, with T1 representing the lowest tertile and serving as the reference group. T2 and T3 represented the middle and highest tertiles, respectively. β coefficients represent the difference in annual change in cognitive z-score compared with T1. Models included time, mean frailty burden tertile, and the interaction between time and mean frailty burden tertile, and were adjusted for age, sex, education, marital status, smoking status, drinking status, and body mass index.

**Abbreviations:** BMI, body mass index; CI, confidence interval; CHARLS, China Health and Retirement Longitudinal Study; ELSA, English Longitudinal Study of Ageing; FI, frailty index; HRS, Health and Retirement Study.

**Table S10.** Parameters for global cognitive function trajectories

| **Fit statistic** | **Number of groups in CHARLS** | | | | |  | **Number of groups in ELSA** | | | | |  | **Number of groups in HRS** | | | | |
| --- | --- | --- | --- | --- | --- | --- | --- | --- | --- | --- | --- | --- | --- | --- | --- | --- | --- |
|  | **1** | **2** | **3** | **4** | **5** |  | **1** | **2** | **3** | **4** | **5** |  | **1** | **2** | **3** | **4** | **5** |
| BIC ^a^ | 51224.72 | 44889.79 | 43622.98 | 43160.66 | 43018.04 |  | 60307.82 | 54275.57 | 51975.72 | 51019.66 | 50598.58 |  | 53595.32 | 48746.94 | 47358.08 | 46809.81 | 46708.58 |
| AIC ^a^ | 51198.01 | 44836.36 | 43542.84 | 43053.81 | 42884.47 |  | 60280.33 | 54220.59 | 51893.25 | 50909.7 | 50461.12 |  | 53568.15 | 48692.61 | 47276.58 | 46701.15 | 46572.75 |
| Group proportion ^b^ | Group 1, 100% | Group 1, 35.86% | Group 1, 46.65% | Group 1, 24.63% | Group 1, 18.94% |  | Group 1, 100% | Group 1, 19.83% | Group 1, 50.11% | Group 1, 56.29% | Group 1, 8.13% |  | Group 1, 100% | Group 1, 27.19% | Group 1, 37.18% | Group 1, 20.89% | Group 1, 41.59% |
|  |  | Group 2, 64.14% | Group 2, 18.82% | Group 2, 40.66% | Group 2, 7.44% |  |  | Group 2, 80.17% | Group 2, 8.82% | Group 2, 5.66% | Group 2, 2.38% |  |  | Group 2, 72.81% | Group 2, 14.51% | Group 2, 21.78% | Group 2, 31.69% |
|  |  |  | Group 3, 34.53% | Group 3, 12.68% | Group 3, 14.60% |  |  |  | Group 3, 41.07% | Group 3, 19.32% | Group 3, 13.93% |  |  |  | Group 3, 48.31% | Group 3, 4.27% | Group 3, 2.97% |
|  |  |  |  | Group 4, 22.04% | Group 4, 41.93% |  |  |  |  | Group 4, 18.73% | Group 4, 51.60% |  |  |  |  | Group 4, 53.06% | Group 4, 15.02% |
|  |  |  |  |  | Group 5, 17.09% |  |  |  |  |  | Group 5, 23.96% |  |  |  |  |  | Group 5, 8.73% |
| APP ^c^ |  | Group 1, 0.93 | Group 1, 0.91 | Group 1, 0.81 | Group 1,  0.74 |  |  | Group 1, 0.91 | Group 1, 0.90 | Group 1, 0.85 | Group 1, 0.85 |  |  | Group 1, 0.9 | Group 1, 0.86 | Group 1, 0.84 | Group 1, 0.76 |
|  |  | Group 2, 0.96 | Group 2, 0.90 | Group 2, 0.79 | Group 2,  0.66 |  |  | Group 2, 0.97 | Group 2, 0.93 | Group 2, 0.91 | Group 2, 0.91 |  |  | Group 2, 0.95 | Group 2, 0.89 | Group 2, 0.85 | Group 2, 0.74 |
|  |  |  | Group 3, 0.83 | Group 3, 0.88 | Group 3,  0.88 |  |  |  | Group 3, 0.86 | Group 3, 0.86 | Group 3, 0.81 |  |  |  | Group 3, 0.85 | Group 3, 0.87 | Group 3, 0.87 |
|  |  |  |  | Group 4, 0.83 | Group 4,  0.79 |  |  |  |  | Group 4, 0.85 | Group 4, 0.84 |  |  |  |  | Group 4, 0.83 | Group 4, 0.81 |
|  |  |  |  |  | Group 5,  0.81 |  |  |  |  |  | Group 5,  0.81 |  |  |  |  |  | Group 5,  0.78 |

CHARLS, China Health and Retirement Longitudinal Study; ELSA, English Longitudinal Study of Ageing; HRS, Health and Retirement Study.

AIC: Akaike’s information criterion; BIC: Bayesian information criterion; APP: average posterior probability.

a: Lower AIC and BIC values indicate better relative fit among candidate models fitted to the same data.

b: No less than 2% of total count in a group.

c: The higher value is better (> 0.7 in a group).

**Table S11.** Parameters for memory trajectories

| Fit statistic | **Number of groups in CHARLS** | | | | |  | **Number of groups in ELSA** | | | | |  | **Number of groups in HRS** | | | | |
| --- | --- | --- | --- | --- | --- | --- | --- | --- | --- | --- | --- | --- | --- | --- | --- | --- | --- |
|  | 1 | **2** | **3** | **4** | **5** |  | 1 | **2** | **3** | **4** | **5** |  | 1 | **2** | **3** | **4** | **5** |
| BIC ^a^ | 49624.24 | 46316.94 | 45788.14 | 45655.81 | 45586.32 |  | 59072.44 | 54023.78 | 51855.04 | 51194.37 | 51067.29 |  | 53446.43 | 49961.25 | 48769.54 | 48501.87 | 48486.64 |
| AIC ^a^ | 49597.52 | 46263.51 | 45707.99 | 45548.96 | 45452.75 |  | 59044.95 | 53968.8 | 51772.57 | 51084.4 | 50929.84 |  | 53419.27 | 49906.92 | 48688.05 | 48393.22 | 48350.81 |
| Group proportion ^b^ | Group 1, 100% | Group 1, 46.34% | Group 1, 15.28% | Group 1, 7.57% | Group 1, 39.86% |  | Group 1, 100% | Group 1, 33.67% | Group 1, 58.66% | Group 1, 8.61% | Group 1, 23.54% |  | Group 1, 100% | Group 1, 44.83% | Group 1, 20.06% | Group 1, 41.35% | Group 1, 2.13% |
|  |  | Group 2, 53.66% | Group 2, 51.29% | Group 2, 49.15% | Group 2, 5.80% |  |  | Group 2, 66.33% | Group 2, 24.14% | Group 2, 27.92% | Group 2, 7.05% |  |  | Group 2, 55.17% | Group 2, 20.07% | Group 2, 10.42% | Group 2, 16.83% |
|  |  |  | Group 3, 33.42% | Group 3, 16.27% | Group 3, 21.99% |  |  |  | Group 3, 17.20% | Group 3, 13.46% | Group 3, 20.16% |  |  |  | Group 3, 59.87% | Group 3, 8.55% | Group 3, 7.77% |
|  |  |  |  | Group 4, 27.01% | Group 4, 11.64% |  |  |  |  | Group 4, 50.01% | Group 4, 44.88% |  |  |  |  | Group 4, 39.69% | Group 4, 27.97% |
|  |  |  |  |  | Group 5, 20.71% |  |  |  |  |  | Group 5, 4.37% |  |  |  |  |  | Group 5, 45.30% |
| APP ^c^ |  | Group 1, 0.90 | Group 1, 0.81 | Group 1, 0.66 | Group 1, 0.73 |  |  | Group 1, 0.90 | Group 1, 0.89 | Group 1, 0.88 | Group 1, 0.75 |  |  | Group 1, 0.89 | Group 1, 0.86 | Group 1, 0.79 | Group 1, 0.77 |
|  |  | Group 2, 0.91 | Group 2, 0.81 | Group 2, 0.77 | Group 2, 0.79 |  |  | Group 2, 0.93 | Group 2, 0.87 | Group 2, 0.81 | Group 2, 0.86 |  |  | Group 2, 0.89 | Group 2, 0.84 | Group 2, 0.83 | Group 2, 0.73 |
|  |  |  | Group 3, 0.88 | Group 3, 0.80 | Group 3, 0.79 |  |  |  | Group 3, 0.90 | Group 3, 0.86 | Group 3, 0.79 |  |  |  | Group 3, 0.86 | Group 3, 0.81 | Group 3, 0.80 |
|  |  |  |  | Group 4, 0.81 | Group 4, 0.66 |  |  |  |  | Group 4, 0.85 | Group 4, 0.80 |  |  |  |  | Group 4, 0.78 | Group 4, 0.73 |
|  |  |  |  |  | Group 5, 0.58 |  |  |  |  |  | Group 5, 0.81 |  |  |  |  |  | Group 5, 0.74 |

CHARLS, China Health and Retirement Longitudinal Study; ELSA, English Longitudinal Study of Ageing; HRS, Health and Retirement Study.

AIC: Akaike’s information criterion; BIC: Bayesian information criterion; APP: average posterior probability.

a: Lower AIC and BIC values indicate better relative fit among candidate models fitted to the same data.

b: No less than 2% of total count in a group.

c: The higher value is better (> 0.7 in a group).

**Table S12.** Parameters for orientation trajectories

| Fit statistic | **Number of groups in CHARLS** | | | | |  |  | **Number of groups in ELSA** | | | |  |  | **Number of groups in HRS** | | | |
| --- | --- | --- | --- | --- | --- | --- | --- | --- | --- | --- | --- | --- | --- | --- | --- | --- | --- |
|  | 1 | **2** | **3** | **4** | **5** |  | 1 | **2** | **3** | **4** | **5** |  | 1 | **2** | **3** | **4** | **5** |
| BIC ^a^ | 46876.71 | 43219.38 | 42640.75 | 41892.19 | 41926.91 |  | 63416.36 | 57850.4 | 56030.59 | 54391.24 | 56284.18 |  | 54970.6 | 50522.36 | 50557.52 | 47677.67 | 49041.48 |
| AIC ^a^ | 46850 | 43165.95 | 42560.61 | 41785.34 | 41793.34 |  | 63388.87 | 57795.42 | 55948.12 | 54281.28 | 56146.73 |  | 54943.44 | 50468.03 | 50476.03 | 47569.01 | 48905.66 |
| Group proportion ^b^ | Group 1, 100% | Group 1, 78.23% | Group 1, 75.71% | Group 1, 8.12% | Group 1, 8.12% |  | Group 1, 100% | Group 1, 3.71% | Group 1, 2.4% | Group 1, 6.11% | Group 1, 1.42% |  | Group 1, 100% | Group 1, 95.07% | Group 1, 94.46% | Group 1, 1.29% | Group 1, 3.76% |
|  |  | Group 2, 21.77% | Group 2, 8.56% | Group 2, 64.87% | Group 2, 18.4% |  |  | Group 2, 96.29% | Group 2, 95.07% | Group 2, 1.32% | Group 2, 5.19% |  |  | Group 2, 4.93% | Group 2, 5.54% | Group 2, 91.89% | Group 2, 93.49% |
|  |  |  | Group 3, 15.72% | Group 3, 15.64% | Group 3, 61.42% |  |  |  | Group 3, 2.54% | Group 3, 90.38% | Group 3, 93.4% |  |  |  | Group 3, 0.00% | Group 3, 3.18% | Group 3, 2.75% |
|  |  |  |  | Group 4, 11.37% | Group 4, 12.07% |  |  |  |  | Group 4, 2.19% | Group 4, 0.00% |  |  |  |  | Group 4, 3.63% | Group 4, 0.00% |
|  |  |  |  |  | Group 5, 0.00% |  |  |  |  |  | Group 5, 0.00% |  |  |  |  |  | Group 5, 0.00% |
| APP ^c^ |  | Group 1, 0.97 | Group 1, 0.96 | Group 1, 0.86 | Group 1, 0.86 |  |  | Group 1, 0.98 | Group 1, 0.97 | Group 1, 0.93 | Group 1, 0.99 |  |  | Group 1, 1 | Group 1, 0.6 | Group 1, 0.98 | Group 1, 0.94 |
|  |  | Group 2, 0.89 | Group 2, 0.78 | Group 2, 0.94 | Group 2, 0.71 |  |  | Group 2, 1 | Group 2, 0.99 | Group 2, 0.99 | Group 2, 0.90 |  |  | Group 2, 0.94 | Group 2, 0.89 | Group 2, 0.99 | Group 2, 0.74 |
|  |  |  | Group 3, 0.86 | Group 3, 0.75 | Group 3, 0.62 |  |  |  | Group 3, 0.97 | Group 3, 0.99 | Group 3, 0.85 |  |  |  | Group 3, NA | Group 3, 0.96 | Group 3, 0.96 |
|  |  |  |  | Group 4, 0.78 | Group 4, 0.76 |  |  |  |  | Group 4, 0.99 | Group 4, NA |  |  |  |  | Group 4, 0.97 | Group 4, NA |
|  |  |  |  |  | Group 5, NA |  |  |  |  |  | Group 5, NA |  |  |  |  |  | Group 5, NA |

CHARLS, China Health and Retirement Longitudinal Study; ELSA, English Longitudinal Study of Ageing; HRS, Health and Retirement Study.

AIC: Akaike’s information criterion; BIC: Bayesian information criterion; APP: average posterior probability; NA, not estimable because no participants were assigned to the corresponding group.

a: Lower AIC and BIC values indicate better relative fit among candidate models fitted to the same data.

b: No less than 2% of total count in a group.

c: The higher value is better (> 0.7 in a group).

**Table S13.** Parameters for executive function trajectories

| Fit statistic | **Number of groups in CHARLS** | | | | |  | **Number of groups in ELSA** | | | | |  | **Number of groups in HRS** | | | | |
| --- | --- | --- | --- | --- | --- | --- | --- | --- | --- | --- | --- | --- | --- | --- | --- | --- | --- |
|  | 1 | **2** | **3** | **4** | **5** |  | 1 | **2** | **3** | **4** | **5** |  | 1 | **2** | **3** | **4** | **5** |
| BIC ^a^ | 47325.19 | 41455.23 | 40088.15 | 39542.57 | 39577.28 |  | 58887.35 | 49742.67 | 47900.02 | 47935.51 | 47971 |  | 53483.1 | 44779.37 | 43123.84 | 43159 | 42655.74 |
| AIC ^a^ | 47298.47 | 41401.8 | 40008.01 | 39435.71 | 39443.71 |  | 58859.85 | 49687.69 | 47817.55 | 47825.55 | 47833.55 |  | 53455.94 | 44725.04 | 43042.35 | 43050.35 | 42519.92 |
| Group proportion | Group 1, 100% | Group 1, 26.31% | Group 1, 24.18% | Group 1, 51.84% | Group 1, 49.8% |  | Group 1, 100% | Group 1, 12.84% | Group 1, 81.82% | Group 1, 13.02% | Group 1, 6.13% |  | Group 1, 100% | Group 1, 25.81% | Group 1, 65.36% | Group 1, 9.5% | Group 1, 12.04% |
|  |  | Group 2, 73.69% | Group 2, 19.11% | Group 2, 18.09% | Group 2, 9.43% |  |  | Group 2, 87.16% | Group 2, 6.13% | Group 2, 80.85% | Group 2, 80.39% |  |  | Group 2, 74.19% | Group 2, 9.5% | Group 2, 26.34% | Group 2, 15.78% |
|  |  |  | Group 3, 56.71% | Group 3, 21.39% | Group 3, 22.69% |  |  |  | Group 3, 12.05% | Group 3, 6.13% | Group 3, 13.48% |  |  |  | Group 3, 25.14% | Group 3, 64.16% | Group 3, 62.65% |
|  |  |  |  | Group 4, 8.68% | Group 4, 18.09% |  |  |  |  | Group 4, 0.00% | Group 4, 0.00% |  |  |  |  | Group 4, 0.00% | Group 4, 9.52% |
|  |  |  |  |  | Group 5, 0.00% |  |  |  |  |  | Group 5, 0.00% |  |  |  |  |  | Group 5, 0.00% |
| APP ^c^ |  | Group 1, 0.95 | Group 1, 0.82 | Group 1, 0.92 | Group 1, 0.59 |  |  | Group 1, 0.96 | Group 1, 0.98 | Group 1, 0.84 | Group 1, 0.96 |  |  | Group 1, 0.93 | Group 1, 0.97 | Group 1, 0.93 | Group 1, 0.72 |
|  |  | Group 2, 0.97 | Group 2, 0.92 | Group 2, 0.93 | Group 2, 0.72 |  |  | Group 2, 0.99 | Group 2, 0.96 | Group 2, 0.59 | Group 2, 0.38 |  |  | Group 2, 0.99 | Group 2, 0.93 | Group 2, 0.84 | Group 2, 0.78 |
|  |  |  | Group 3, 0.92 | Group 3, 0.81 | Group 3, 0.78 |  |  |  | Group 3, 0.87 | Group 3, 0.96 | Group 3, 0.82 |  |  |  | Group 3, 0.85 | Group 3, 0.68 | Group 3, 0.51 |
|  |  |  |  | Group 4, 0.74 | Group 4, 0.93 |  |  |  |  | Group 4,  NA | Group 4,  NA |  |  |  |  | Group 4,  NA | Group 4, 0.92 |
|  |  |  |  |  | Group 5,  NA |  |  |  |  |  | Group 5,  NA |  |  |  |  |  | Group 5,  NA |

CHARLS, China Health and Retirement Longitudinal Study; ELSA, English Longitudinal Study of Ageing; HRS, Health and Retirement Study. AIC: Akaike’s information criterion; BIC: Bayesian information criterion; APP: average posterior probability; NA, not estimable because no participants were assigned to the corresponding group.

a: Lower AIC and BIC values indicate better relative fit among candidate models fitted to the same data.

b: No less than 2% of total count in a group.

c: The higher value is better (> 0.7 in a group).

**Table S14.** Association of baseline frailty status with trajectories in different cognitive domains

| **Frailty status** | | **CHARLS (n = 5876)** | | | | |  | **ELSA (n = 7134)** | | | | |  | **HRS (n = 6576)** | | | | |
| --- | --- | --- | --- | --- | --- | --- | --- | --- | --- | --- | --- | --- | --- | --- | --- | --- | --- | --- |
|  |  | **Low-score trajectory (vs high-score trajectory)** | |  | **Moderate-score trajectory (vs high-score trajectory)** | |  | **Low-score trajectory (vs high-score trajectory)** | |  | **Moderate-score trajectory (vs high-score trajectory)** | |  | **Low-score trajectory (vs high-score trajectory)** | |  | **Moderate-score trajectory (vs high-score trajectory)** | |
|  |  | **OR (95% CI)^*^** | ***p*** |  | **OR (95% CI)^*^** | ***p*** |  | **OR (95% CI)^*^** | ***p*** |  | **OR (95% CI)^*^** | ***p*** |  | **OR (95% CI)^*^** | ***p*** |  | **OR (95% CI)^*^** | ***p*** |
| Global cognitive trajectories | |  |  |  |  |  |  |  |  |  |  |  |  |  |  |  |  |  |
|  | Robust | 1 (reference) |  |  | 1 (reference) |  |  | 1 (reference) |  |  | 1 (reference) |  |  | 1 (reference) |  |  | 1 (reference) |  |
|  | Pre-frail | 1.57 (1.32, 1.86) | < 0.001 |  | 1.29 (1.13, 1.47) | < 0.001 |  | 1.65 (1.29, 2.11) | < 0.001 |  | 1.30 (1.15, 1.47) | < 0.001 |  | 1.75 (1.35, 2.27) | < 0.001 |  | 1.48 (1.28, 1.72) | < 0.001 |
|  | Frail | 3.00 (2.36, 3.81) | < 0.001 |  | 2.02 (1.65, 2.48) | < 0.001 |  | 3.15 (2.39, 4.14) | < 0.001 |  | 1.92 (1.62, 2.27) | < 0.001 |  | 4.02 (3.08, 5.24) | < 0.001 |  | 2.15 (1.82, 2.54) | < 0.001 |
| Memory trajectories | |  |  |  |  |  |  |  |  |  |  |  |  |  |  |  |  |  |
|  | Robust | 1 (reference) |  |  | 1 (reference) |  |  | 1 (reference) |  |  | 1 (reference) |  |  | 1 (reference) |  |  | 1 (reference) |  |
|  | Pre-frail | 1.77 (1.46, 2.16) | < 0.001 |  | 1.48 (1.25, 1.76) | < 0.001 |  | 1.70 (1.38, 2.09) | < 0.001 |  | 1.26 (1.10, 1.45) | < 0.001 |  | 2.00 (1.57, 2.55) | < 0.001 |  | 1.65 (1.41, 1.94) | < 0.001 |
|  | Frail | 3.79 (2.70, 5.32) | < 0.001 |  | 2.35 (1.70, 3.25) | < 0.001 |  | 3.38 (2.57, 4.44) | < 0.001 |  | 1.87 (1.51, 2.32) | < 0.001 |  | 3.71 (2.85, 4.82) | < 0.001 |  | 2.04 (1.68, 2.48) | < 0.001 |
| Orientation trajectories | |  |  |  |  |  |  |  |  |  |  |  |  |  |  |  |  |  |
|  | Robust | 1 (reference) |  |  |  |  |  | 1 (reference) |  |  |  |  |  | 1 (reference) |  |  |  |  |
|  | Pre-frail | 1.21 (1.04, 1.40) | < 0.05 |  |  |  |  | 1.27 (0.91, 1.78) | 0.16 |  |  |  |  | 1.38 (0.95, 2.01) | 0.087 |  |  |  |
|  | Frail | 1.98 (1.64, 2.39) | < 0.001 |  |  |  |  | 2.06 (1.44, 2.95) | < 0.001 |  |  |  |  | 2.09 (1.44, 3.04) | < 0.001 |  |  |  |
| Executive function trajectories | |  |  |  |  |  |  |  |  |  |  |  |  |  |  |  |  |  |
|  | Robust | 1 (reference) |  |  | 1 (reference) |  |  | 1 (reference) |  |  | 1 (reference) |  |  | 1 (reference) |  |  | 1 (reference) |  |
|  | Pre-frail | 1.40 (1.19, 1.65) | < 0.001 |  | 1.14 (1.00, 1.32) | < 0.05 |  | 1.45 (1.11, 1.89) | < 0.01 |  | 1.28 (1.07, 1.53) | < 0.01 |  | 1.47 (1.09, 2.00) | < 0.05 |  | 1.30 (1.10, 1.53) | < 0.01 |
|  | Frail | 2.16 (1.74, 2.69) | < 0.001 |  | 1.60 (1.31, 1.96) | < 0.001 |  | 2.33 (1.76, 3.13) | < 0.001 |  | 1.40 (1.13, 1.73) | < 0.01 |  | 2.58 (1.92, 3.48) | < 0.001 |  | 1.72 (1.44, 2.05) | < 0.001 |
| CHARLS, China Health and Retirement Longitudinal Study; ELSA, English Longitudinal Study of Ageing; HRS, Health and Retirement Study. OR, odds ratio; CI, confidence interval. *Adjusted for age, sex, education, marital status, smoking status, drinking status, and body mass index. | | | | | | | | | | | | | | | | | | |

**Table S15.** Association of two-wave frailty change with trajectories in different cognitive domains

| **Two-wave frailty change** | | **CHARLS (n = 5876)** | | | | |  | **ELSA (n = 7134)** | | | | |  | **HRS (n = 6576)** | | | | |
| --- | --- | --- | --- | --- | --- | --- | --- | --- | --- | --- | --- | --- | --- | --- | --- | --- | --- | --- |
|  |  | **Low-score trajectory (vs high-score trajectory)** | |  | **Moderate-score trajectory (vs high-score trajectory)** | |  | **Low-score trajectory (vs high-score trajectory)** | |  | **Moderate-score trajectory (vs high-score trajectory)** | |  | **Low-score trajectory (vs high-score trajectory)** | |  | **Moderate-score trajectory (vs high-score trajectory)** | |
|  |  | **OR (95% CI)**^*^ | ***p*** |  | **OR (95% CI)**^*^ | ***p*** |  | **OR (95% CI)**^*^ | ***p*** |  | **OR (95% CI)**^*^ | ***p*** |  | **OR (95% CI)**^*^ | ***p*** |  | **OR (95% CI)**^*^ | ***p*** |
| **Global cognitive trajectories** | |  |  |  |  |  |  |  |  |  |  |  |  |  |  |  |  |  |
|  | Stable robust | 1 (reference) |  |  | 1 (reference) |  |  | 1 (reference) |  |  | 1 (reference) |  |  | 1 (reference) |  |  | 1 (reference) |  |
|  | Robust to pre-frail/frail | 1.69 (1.31, 2.17) | < 0.001 |  | 1.35 (1.12, 1.63) | <0.01 |  | 1.24 (0.83, 1.85) | 0.295 |  | 1.34 (1.11, 1.63) | <0.01 |  | 1.49 (0.98, 2.27) | 0.065 |  | 1.39 (1.10, 1.76) | <0.01 |
|  | Stable pre-frail | 1 (reference) |  |  | 1 (reference) |  |  | 1 (reference) |  |  | 1 (reference) |  |  | 1 (reference) |  |  | 1 (reference) |  |
|  | Pre-frail to robust | 0.81 (0.59, 1.10) | 0.177 |  | 0.85 (0.67, 1.08) | 0.193 |  | 0.81 (0.51, 1.30) | 0.385 |  | 0.92 (0.72, 1.17) | 0.495 |  | 0.65 (0.37, 1.14) | 0.133 |  | 0.87 (0.64, 1.19) | 0.381 |
|  | Pre-frail to frail | 1.84 (1.32, 2.55) | <0.001 |  | 1.51 (1.14, 2.01) | <0.01 |  | 1.60 (1.06, 2.41) | <0.05 |  | 1.19 (0.90, 1.58) | 0.253 |  | 1.38 (1.00, 1.92) | <0.05 |  | 1.21 (0.97, 1.52) | 0.088 |
|  | Stable frail | 1 (reference) |  |  | 1 (reference) |  |  | 1 (reference) |  |  | 1 (reference) |  |  | 1 (reference) |  |  | 1 (reference) |  |
|  | Frail to robust/pre-frail | 0.94 (0.60, 1.46) | 0.777 |  | 0.89 (0.59, 1.34) | 0.571 |  | 0.94 (0.59, 1.48) | 0.777 |  | 0.74 (0.53, 1.03) | 0.071 |  | 0.66 (0.43, 1.01) | 0.056 |  | 0.84 (0.70, 1.00) | 0.055 |
| **Memory trajectories** | |  |  |  |  |  |  |  |  |  |  |  |  |  |  |  |  |  |
|  | Stable robust | 1 (reference) |  |  | 1 (reference) |  |  | 1 (reference) |  |  | 1 (reference) |  |  | 1 (reference) |  |  | 1 (reference) |  |
|  | Robust to pre-frail/frail | 1.66 (1.27, 2.18) | <0.001 |  | 1.32 (1.04, 1.68) | <0.05 |  | 1.50 (1.08, 2.08) | <0.05 |  | 1.26 (1.01, 1.57) | <0.05 |  | 1.27 (0.85, 1.90) | 0.239 |  | 1.43 (1.10, 1.86) | <0.01 |
|  | Stable pre-frail | 1 (reference) |  |  | 1 (reference) |  |  | 1 (reference) |  |  | 1 (reference) |  |  | 1 (reference) |  |  | 1 (reference) |  |
|  | Pre-frail to robust | 0.60 (0.42, 0.84) | <0.01 |  | 0.62 (0.46, 0.85) | <0.01 |  | 0.96 (0.65, 1.41) | 0.819 |  | 0.91 (0.69, 1.21) | 0.528 |  | 0.54 (0.33, 0.89) | <0.05 |  | 0.61 (0.43, 0.86) | <0.01 |
|  | Pre-frail to frail | 2.00 (1.24, 3.23) | <0.01 |  | 1.50 (0.95, 2.36) | 0.084 |  | 1.38 (0.90, 2.11) | 0.145 |  | 1.01 (0.68, 1.41) | 0.912 |  | 1.11 (0.79, 1.56) | 0.556 |  | 1.02 (0.71, 1.44) | 0.864 |
|  | Stable frail | 1 (reference) |  |  | 1 (reference) |  |  | 1 (reference) |  |  | 1 (reference) |  |  | 1 (reference) |  |  | 1 (reference) |  |
|  | Frail to robust/pre-frail | 0.73 (0.39, 1.39) | 0.341 |  | 0.68 (0.36, 1.27) | 0.225 |  | 0.81 (0.49, 1.36) | 0.431 |  | 0.66 (0.43, 1.03) | 0.069 |  | 0.84 (0.52, 1.35) | 0.472 |  | 0.85 (0.56, 1.28) | 0.434 |
| **Orientation trajectories** | |  |  |  |  |  |  |  |  |  |  |  |  |  |  |  |  |  |
|  | Stable robust | 1 (reference) |  |  |  |  |  | 1 (reference) |  |  |  |  |  | 1 (reference) |  |  |  |  |
|  | Robust to pre-frail/frail | 1.24 (1.01, 1.53) | <0.05 |  |  |  |  | 1.28 (0.86, 1.92) | 0.245 |  |  |  |  | 1.17 (0.64, 2.14) | 0.6 |  |  |  |
|  | Stable pre-frail | 1 (reference) |  |  |  |  |  | 1 (reference) |  |  |  |  |  | 1 (reference) |  |  |  |  |
|  | Pre-frail to robust | 0.90 (0.69, 1.17) | 0.411 |  |  |  |  | 0.66 (0.33, 1.32) | 0.241 |  |  |  |  | 0.56 (0.22, 1.42) | 0.228 |  |  |  |
|  | Pre-frail to frail | 1.67 (1.29, 2.17) | <0.001 |  |  |  |  | 1.13 (0.66, 1.92) | 0.666 |  |  |  |  | 1.49 (0.99, 2.25) | 0.054 |  |  |  |
|  | Stable frail | 1 (reference) |  |  |  |  |  | 1 (reference) |  |  |  |  |  | 1 (reference) |  |  |  |  |
|  | Frail to robust/pre-frail | 0.91 (0.64, 1.29) | 0.597 |  |  |  |  | 0.99 (0.58, 1.71) | 0.991 |  |  |  |  | 0.53 (0.31, 0.90) | <0.05 |  |  |  |
| **Executive function trajectories** | |  |  |  |  |  |  |  |  |  |  |  |  |  |  |  |  |  |
|  | Stable robust | 1 (reference) |  |  | 1 (reference) |  |  | 1 (reference) |  |  | 1 (reference) |  |  | 1 (reference) |  |  | 1 (reference) |  |
|  | Robust to pre-frail/frail | 1.62 (1.28, 2.04) | <0.001 |  | 1.20 (0.99, 1.46) | 0.067 |  | 1.22 (0.79, 1.88) | 0.36 |  | 1.49 (1.15, 1.94) | <0.01 |  | 1.59 (1.01, 2.51) | <0.05 |  | 1.31 (1.01, 1.71) | <0.05 |
|  | Stable pre-frail | 1 (reference) |  |  | 1 (reference) |  |  | 1 (reference) |  |  | 1 (reference) |  |  | 1 (reference) |  |  | 1 (reference) |  |
|  | Pre-frail to robust | 0.88 (0.66, 1.18) | 0.387 |  | 0.94 (0.73, 1.21) | 0.624 |  | 0.89 (0.54, 1.47) | 0.646 |  | 0.92 (0.70, 1.23) | 0.526 |  | 0.48 (0.23, 0.99) | <0.05 |  | 0.85 (0.60, 1.19) | 0.339 |
|  | Pre-frail to frail | 1.33 (0.98, 1.79) | 0.066 |  | 1.33 (1.01, 1.75) | <0.05 |  | 1.51 (1.01, 2.27) | <0.05 |  | 1.04 (0.73, 1.47) | 0.834 |  | 1.25 (0.87, 1.80) | 0.217 |  | 1.17 (0.94, 1.46) | 0.151 |
|  | Stable frail | 1 (reference) |  |  | 1 (reference) |  |  | 1 (reference) |  |  | 1 (reference) |  |  | 1 (reference) |  |  | 1 (reference) |  |
|  | Frail to robust/pre-frail | 0.74 (0.50, 1.11) | 0.147 |  | 0.76 (0.51, 1.13) | 0.179 |  | 0.77 (0.47, 1.27) | 0.305 |  | 1.00 (0.68, 1.46) | 0.988 |  | 0.67 (0.43, 1.03) | 0.067 |  | 0.66 (0.48, 0.91) | <0.05 |
| CHARLS, China Health and Retirement Longitudinal Study; ELSA, English Longitudinal Study of Ageing; HRS, Health and Retirement Study. OR, odds ratio; CI, confidence interval. *Adjusted for age, sex, education, marital status, smoking status, drinking status, and body mass index. | | | | | | | | | | | | | | | | | | |

**Table S16.** Association of mean frailty burden with trajectories in different cognitive domains

| **Mean frailty burden** | | **CHARLS (n = 5876)** | | | | |  | **ELSA (n = 7134)** | | | | |  | **HRS (n = 6576)** | | | | |
| --- | --- | --- | --- | --- | --- | --- | --- | --- | --- | --- | --- | --- | --- | --- | --- | --- | --- | --- |
|  |  | **Low-score trajectory (vs high-score trajectory)** | |  | **Moderate-score trajectory (vs high-score trajectory)** | |  | **Low-score trajectory (vs high-score trajectory)** | |  | **Moderate-score trajectory (vs high-score trajectory)** | |  | **Low-score trajectory (vs high-score trajectory)** | |  | **Moderate-score trajectory (vs high-score trajectory)** | |
|  |  | **OR (95% CI)**^*^ | ***p*** |  | **OR (95% CI)**^*^ | ***p*** |  | **OR (95% CI)**^*^ | ***p*** |  | **OR (95% CI)**^*^ | ***p*** |  | **OR (95% CI)**^*^ | ***p*** |  | **OR (95% CI)**^*^ | ***p*** |
| **Global cognitive trajectories** | |  |  |  |  |  |  |  |  |  |  |  |  |  |  |  |  |  |
|  | T1 | 1 (reference) |  |  | 1 (reference) |  |  | 1 (reference) |  |  | 1 (reference) |  |  | 1 (reference) |  |  | 1 (reference) |  |
|  | T2 | 1.25 (1.02, 1.54) | < 0.05 |  | 1.26 (1.09, 1.46) | < 0.01 |  | 1.11 (0.84, 1.47) | 0.47 |  | 1.14 (1.01, 1.30) | < 0.05 |  | 1.77 (1.40, 2.25) | < 0.001 |  | 1.54 (1.34, 1.76) | < 0.001 |
|  | T3 | 2.44 (2.00, 2.99) | < 0.001 |  | 1.74 (1.49, 2.04) | < 0.001 |  | 2.74 (2.08, 3.60) | < 0.001 |  | 1.66 (1.43, 1.93) | < 0.001 |  | 4.24 (3.33, 5.40) | < 0.001 |  | 2.15 (1.83, 2.53) | < 0.001 |
|  | *P for trend* | < 0.001 |  |  | < 0.001 |  |  | < 0.001 |  |  | < 0.001 |  |  | < 0.001 |  |  | < 0.001 |  |
| **Memory trajectories** | |  |  |  |  |  |  |  |  |  |  |  |  |  |  |  |  |  |
|  | T1 | 1 (reference) |  |  | 1 (reference) |  |  | 1 (reference) |  |  | 1 (reference) |  |  | 1 (reference) |  |  | 1 (reference) |  |
|  | T2 | 1.32 (1.07, 1.63) | < 0.01 |  | 1.19 (0.99, 1.42) | 0.06 |  | 1.36 (1.08, 1.71) | < 0.01 |  | 1.24 (1.08, 1.43) | < 0.01 |  | 2.08 (1.66, 2.61) | < 0.001 |  | 1.61 (1.37, 1.89) | < 0.001 |
|  | T3 | 2.70 (2.13, 3.41) | < 0.001 |  | 1.74 (1.40, 2.15) | < 0.001 |  | 3.00 (2.35, 3.84) | < 0.001 |  | 1.60 (1.34, 1.91) | < 0.001 |  | 4.11 (3.20, 5.28) | < 0.001 |  | 2.09 (1.72, 2.54) | < 0.001 |
|  | *P for trend* | <0.001 |  |  | <0.001 |  |  | <0.001 |  |  | <0.001 |  |  | <0.001 |  |  | <0.001 |  |
| **Orientation trajectories** | |  |  |  |  |  |  |  |  |  |  |  |  |  |  |  |  |  |
|  | T1 | 1 (reference) |  |  |  |  |  | 1 (reference) |  |  |  |  |  | 1 (reference) |  |  |  |  |
|  | T2 | 1.07 (0.90, 1.27) | 0.458 |  |  |  |  | 1.13 (0.76, 1.67) | 0.54 |  |  |  |  | 1.30 (0.93, 1.82) | 0.13 |  |  |  |
|  | T3 | 1.66 (1.41, 1.96) | < 0.001 |  |  |  |  | 2.03 (1.40, 2.96) | < 0.001 |  |  |  |  | 2.34 (1.68, 3.26) | < 0.001 |  |  |  |
|  | *P for trend* | < 0.001 |  |  |  |  |  | < 0.001 |  |  |  |  |  | < 0.001 |  |  |  |  |
| **Executive function trajectories** | |  |  |  |  |  |  |  |  |  |  |  |  |  |  |  |  |  |
|  | T1 | 1 (reference) |  |  | 1 (reference) |  |  | 1 (reference) |  |  | 1 (reference) |  |  | 1 (reference) |  |  | 1 (reference) |  |
|  | T2 | 1.26 (1.04, 1.53) | < 0.05 |  | 1.10 (0.94, 1.29) | 0.232 |  | 1.15 (0.84, 1.56) | 0.38 |  | 1.26 (1.03, 1.53) | < 0.05 |  | 1.44 (1.10, 1.89) | < 0.01 |  | 1.35 (1.16, 1.57) | < 0.001 |
|  | T3 | 1.87 (1.55, 2.26) | < 0.001 |  | 1.42 (1.21, 1.67) | < 0.001 |  | 2.23 (1.67, 2.99) | < 0.001 |  | 1.42 (1.15, 1.74) | < 0.001 |  | 2.62 (2.01, 3.41) | < 0.001 |  | 1.68 (1.43, 1.98) | < 0.001 |
|  | *P for trend* | < 0.001 |  |  | < 0.001 |  |  | < 0.001 |  |  | < 0.001 |  |  | < 0.001 |  |  | < 0.001 |  |
| Mean frailty burden was calculated as the mean of the FI at baseline and the FI at the preceding survey. T1 was the lower tertile, T2 was the middle tertile, and T3 was the upper tertile.  CHARLS, China Health and Retirement Longitudinal Study; ELSA, English Longitudinal Study of Ageing; HRS, Health and Retirement Study; FI, frailty index.  OR, odds ratio; CI, confidence interval. *Adjusted for age, sex, education, marital status, smoking status, drinking status, and body mass index. | | | | | | | | | | | | | | | | | | |

****Table S17.** Number and percentage of two-wave frailty change using alternative FI cut-off scheme**

| Preceding survey |  | Baseline  survey | CHARLS  n (%) | ELSA  n (%) | HRS  n (%) |
| --- | --- | --- | --- | --- | --- |
| Robust |  | Robust | 2206 (72.2%) | 3049 (82.5%) | 1360 (73.1%) |
|  |  | Pre-frail | 703 (23.0%) | 587 (15.9%) | 442 (23.8%) |
|  |  | Frail | 145 (4.7%) | 60 (1.6%) | 59 (3.2%) |
| Pre-frail |  | Robust | 485 (25.6%) | 426 (23.4%) | 222 (9.9%) |
|  |  | Pre-frail | 970 (51.1%) | 1018 (55.9%) | 1400 (62.4%) |
|  |  | Frail | 443 (23.3%) | 378 (20.7%) | 622 (27.7%) |
| Frail |  | Robust | 66 (7.1%) | 21 (1.3%) | 11 (0.4%) |
|  |  | Pre-frail | 301 (32.6%) | 255 (15.8%) | 315 (12.7%) |
|  |  | Frail | 557 (60.3%) | 1340 (82.9%) | 2145 (86.8%) |

**Notes.** Values are presented as n (%). Percentages were calculated within each frailty-status category at the preceding survey in each cohort. Two-wave frailty change was defined from the preceding survey to the baseline survey. The alternative FI cut-off scheme defined robust as FI ≤ 0.10, pre-frail as 0.10 < FI ≤ 0.21, and frail as FI > 0.21.

**Abbreviations:** FI, frailty index; CHARLS, China Health and Retirement Longitudinal Study; ELSA, English Longitudinal Study of Ageing; HRS, Health and Retirement Study.

****Table S18.** Association of baseline frailty status with cognitive trajectories using alternative FI cut-off scheme**

| **Frailty status** | | **CHARLS (n = 5876)** | | | | |  | **ELSA (n = 7134)** | | | | |  | **HRS (n = 6576)** | | | | |
| --- | --- | --- | --- | --- | --- | --- | --- | --- | --- | --- | --- | --- | --- | --- | --- | --- | --- | --- |
|  |  | **Low-score trajectory (vs high-score trajectory)** | |  | **Moderate-score trajectory (vs high-score trajectory)** | |  | **Low-score trajectory (vs high-score trajectory)** | |  | **Moderate-score trajectory (vs high-score trajectory)** | |  | **Low-score trajectory (vs high-score trajectory)** | |  | **Moderate-score trajectory (vs high-score trajectory)** | |
|  |  | **OR (95% CI)^*^** | ***p*** |  | **OR (95% CI)^*^** | ***p*** |  | **OR (95% CI)^*^** | ***p*** |  | **OR (95% CI)^*^** | ***p*** |  | **OR (95% CI)^*^** | ***p*** |  | **OR (95% CI)^*^** | ***p*** |
| Global cognitive trajectories | |  |  |  |  |  |  |  |  |  |  |  |  |  |  |  |  |  |
|  | Robust | 1 (reference) |  |  | 1 (reference) |  |  | 1 (reference) |  |  | 1 (reference) |  |  | 1 (reference) |  |  | 1 (reference) |  |
|  | Pre-frail | 1.50 (1.25, 1.80) | <0.001 |  | 1.31 (1.14, 1.50) | <0.001 |  | 1.50 (1.16, 1.94) | <0.05 |  | 1.24 (1.08, 1.41) | <0.05 |  | 1.83 (1.39, 2.40) | <0.001 |  | 1.47 (1.26, 1.71) | <0.001 |
|  | Frail | 2.77 (2.24, 3.42) | <0.001 |  | 1.75 (1.47, 2.09) | <0.001 |  | 3.00 (2.32, 3.89) | <0.001 |  | 1.82 (1.56, 2.12) | <0.001 |  | 3.40 (2.62, 4.41) | <0.001 |  | 2.00 (1.71, 2.34) | <0.001 |
| Memory trajectories | |  |  |  |  |  |  |  |  |  |  |  |  |  |  |  |  |  |
|  | Robust | 1 (reference) |  |  | 1 (reference) |  |  | 1 (reference) |  |  | 1 (reference) |  |  | 1 (reference) |  |  | 1 (reference) |  |
|  | Pre-frail | 1.77 (1.45, 2.17) | <0.001 |  | 1.50 (1.25, 1.79) | <0.001 |  | 1.58 (1.27, 1.97) | <0.001 |  | 1.26 (1.08, 1.46) | <0.05 |  | 1.93 (1.50, 2.48) | <0.001 |  | 1.60 (1.36, 1.90) | <0.001 |
|  | Frail | 3.07 (2.34, 4.03) | <0.001 |  | 1.97 (1.52, 2.54) | <0.001 |  | 2.97 (2.32, 3.79) | <0.001 |  | 1.64 (1.36, 1.99) | <0.001 |  | 3.45 (2.68, 4.43) | <0.001 |  | 2.02 (1.68, 2.42) | <0.001 |
| Orientation trajectories | |  |  |  |  |  |  |  |  |  |  |  |  |  |  |  |  |  |
|  | Robust | 1 (reference) |  |  |  |  |  | 1 (reference) |  |  |  |  |  | 1 (reference) |  |  |  |  |
|  | Pre-frail | 1.19 (1.02, 1.38) | <0.05 |  |  |  |  | 1.20 (0.84, 1.71) | 0.315 |  |  |  |  | 1.39 (0.95, 2.06) | 0.096 |  |  |  |
|  | Frail | 1.81 (1.53, 2.15) | <0.001 |  |  |  |  | 2.04 (1.46, 2.88) | <0.001 |  |  |  |  | 1.97 (1.38, 2.88) | <0.001 |  |  |  |
| Executive function trajectories | |  |  |  |  |  |  |  |  |  |  |  |  |  |  |  |  |  |
|  | Robust | 1 (reference) |  |  | 1 (reference) |  |  | 1 (reference) |  |  | 1 (reference) |  |  | 1 (reference) |  |  | 1 (reference) |  |
|  | Pre-frail | 1.38 (1.16, 1.64) | <0.001 |  | 1.15 (1.00, 1.33) | <0.05 |  | 1.36 (1.02, 1.80) | <0.05 |  | 1.21 (1.00, 1.45) | 0.051 |  | 1.48 (1.08, 2.02) | <0.05 |  | 1.29 (1.09, 1.53) | <0.05 |
|  | Frail | 2.01 (1.66, 2.45) | <0.001 |  | 1.45 (1.22, 1.73) | <0.001 |  | 2.27 (1.73, 2.97) | <0.001 |  | 1.47 (1.21, 1.79) | <0.001 |  | 2.37 (1.77, 3.18) | <0.001 |  | 1.63 (1.37, 1.93) | <0.001 |

**Notes.** The alternative FI cut-off scheme defined robust as FI ≤ 0.10, pre-frail as 0.10 < FI ≤ 0.21, and frail as FI > 0.21. Robust participants served as the **reference group. For each cognitive outcome, the high-score trajectory served as the reference category. Models were adjusted for age, sex, education, marital status, smoking status, drinking status, and body mass index.**

****Abbreviations:** OR, odds ratio; CI, confidence interval; FI, frailty index; CHARLS, China Health and Retirement Longitudinal Study; ELSA, English Longitudinal Study of Ageing; HRS, Health and Retirement Study.**

****Table S19.** Association of two-wave frailty change with cognitive trajectories using alternative FI cut-off scheme**

| **Two-wave frailty change** | | **CHARLS (n = 5876)** | | | | |  | **ELSA (n = 7134)** | | | | | | | | |  | **HRS (n = 6576)** | | | | | |  |
| --- | --- | --- | --- | --- | --- | --- | --- | --- | --- | --- | --- | --- | --- | --- | --- | --- | --- | --- | --- | --- | --- | --- | --- | --- |
|  |  | **Low-score trajectory (vs high-score trajectory)** | |  | **Moderate-score trajectory (vs high-score trajectory)** | |  | **Low-score trajectory (vs high-score trajectory)** | | | |  | | **Moderate-score trajectory (vs high-score trajectory)** | | |  | **Low-score trajectory (vs high-score trajectory)** | |  | **Moderate-score trajectory (vs high-score trajectory)** | | |  |
|  |  | **OR (95% CI)**^*^ | ***p*** |  | **OR (95% CI)**^*^ | ***p*** |  | | **OR (95% CI)**^*^ | | ***p*** |  | **OR (95% CI)**^*^ | | | ***p*** |  | **OR (95% CI)**^*^ | ***p*** |  | **OR (95% CI)**^*^ | | ***p*** | |
| **Global cognitive trajectories** | |  |  |  |  |  |  |  | |  | |  |  | |  | |  |  |  |  |  |  | |  |
|  | Stable robust | 1 (reference) |  |  | 1 (reference) |  |  | 1 (reference) | |  | |  | 1 (reference) | |  | |  | 1 (reference) |  |  | 1 (reference) |  | |  |
|  | Robust to pre-frail/frail | 1.73 (1.35, 2.21) | <0.001 |  | 1.36 (1.13, 1.64) | 0.001 |  | 1.23 (0.82, 1.84) | | 0.310 | |  | 1.33 (1.10, 1.62) | | 0.003 | |  | 1.49 (0.98, 2.27) | 0.064 |  | 1.40 (1.11, 1.77) | 0.005 | |  |
|  | Stable pre-frail | 1 (reference) |  |  | 1 (reference) |  |  | 1 (reference) | |  | |  | 1 (reference) | |  | |  | 1 (reference) |  |  | 1 (reference) |  | |  |
|  | Pre-frail to robust | 0.81 (0.58, 1.14) | 0.230 |  | 0.81 (0.63, 1.05) | 0.112 |  | 0.90 (0.55, 1.48) | | 0.690 | |  | 0.97 (0.75, 1.25) | | 0.818 | |  | 0.62 (0.34, 1.11) | 0.107 |  | 0.94 (0.68, 1.29) | 0.707 | |  |
|  | Pre-frail to frail | 1.70 (1.22, 2.36) | 0.002 |  | 1.28 (0.98, 1.68) | 0.075 |  | 1.74 (1.14, 2.66) | | 0.011 | |  | 1.30 (0.98, 1.71) | | 0.065 | |  | 1.02 (0.72, 1.44) | 0.928 |  | 1.12 (0.90, 1.40) | 0.302 | |  |
|  | Stable frail | 1 (reference) |  |  | 1 (reference) |  |  | 1 (reference) | |  | |  | 1 (reference) | |  | |  | 1 (reference) |  |  | 1 (reference) |  | |  |
|  | Frail to robust/pre-frail | 0.92 (0.64, 1.33) | 0.653 |  | 0.96 (0.69, 1.35) | 0.814 |  | 0.87 (0.55, 1.37) | | 0.542 | |  | 0.82 (0.60, 1.12) | | 0.214 | |  | 0.84 (0.57, 1.26) | 0.405 |  | 0.98 (0.73, 1.31) | 0.877 | |  |
| **Memory trajectories** | |  |  |  |  |  |  |  | |  | |  |  | |  | |  |  |  |  |  |  | |  |
|  | Stable robust | 1 (reference) |  |  | 1 (reference) |  |  | 1 (reference) | |  | |  | 1 (reference) | |  | |  | 1 (reference) |  |  | 1 (reference) |  | |  |
|  | Robust to pre-frail/frail | 1.69 (1.29, 2.21) | <0.001 |  | 1.33 (1.05, 1.69) | 0.019 |  | 1.48 (1.07, 2.06) | | 0.018 | |  | 1.25 (1.01, 1.56) | | 0.044 | |  | 1.28 (0.85, 1.91) | 0.234 |  | 1.43 (1.10, 1.86) | 0.007 | |  |
|  | Stable pre-frail | 1 (reference) |  |  | 1 (reference) |  |  | 1 (reference) | |  | |  | 1 (reference) | |  | |  | 1 (reference) |  |  | 1 (reference) |  | |  |
|  | Pre-frail to robust | 0.55 (0.38, 0.80) | 0.002 |  | 0.59 (0.43, 0.83) | 0.002 |  | 0.96 (0.62, 1.49) | | 0.851 | |  | 0.88 (0.66, 1.18) | | 0.388 | |  | 0.58 (0.34, 0.97) | 0.039 |  | 0.69 (0.49, 0.99) | 0.044 | |  |
|  | Pre-frail to frail | 1.24 (0.81, 1.89) | 0.325 |  | 1.01 (0.68, 1.49) | 0.980 |  | 1.49 (0.97, 2.28) | | 0.066 | |  | 1.03 (0.73, 1.46) | | 0.848 | |  | 1.15 (0.81, 1.64) | 0.428 |  | 1.13 (0.86, 1.48) | 0.396 | |  |
|  | Stable frail | 1 (reference) |  |  | 1 (reference) |  |  | 1 (reference) | |  | |  | 1 (reference) | | |  |  | 1 (reference) |  |  | 1 (reference) | |  | |
|  | Frail to robust/pre-frail | 0.68 (0.40, 1.14) | 0.145 |  | 0.66 (0.40, 1.11) | 0.116 |  | 0.89 (0.55, 1.45) | | 0.641 | |  | 0.88 (0.59, 1.31) | | | 0.523 |  | 0.99 (0.62, 1.57) | 0.964 |  | 0.97 (0.72, 1.34) | | 0.848 | |
| **Orientation trajectories** | |  |  |  |  |  |  |  | |  | |  |  | | |  |  |  |  |  |  | |  | |
|  | Stable robust | 1 (reference) |  |  |  |  |  | 1 (reference) | |  | |  |  | | |  |  | 1 (reference) |  |  |  | |  | |
|  | Robust to pre-frail/frail | 1.26 (1.02, 1.55) | 0.030 |  |  |  |  | 1.21 (0.72, 1.73) | | 0.394 | |  |  | | |  |  | 1.18 (0.63, 2.12) | 0.591 |  |  | |  | |
|  | Stable pre-frail | 1 (reference) |  |  |  |  |  | 1 (reference) | |  | |  |  | | |  |  | 1 (reference) |  |  |  | |  | |
|  | Pre-frail to robust | 0.89 (0.67, 1.19) | 0.439 |  |  |  |  | 0.64 (0.30, 1.25) | | 0.220 | |  |  | | |  |  | 0.55 (0.19, 1.28) | 0.212 |  |  | |  | |
|  | Pre-frail to frail | 1.48 (1.14, 1.94) | 0.004 |  |  |  |  | 1.03 (0.71, 1.48) | | 0.875 | |  |  | | |  |  | 1.12 (0.64, 1.71) | 0.649 |  |  | |  | |
|  | Stable frail | 1 (reference) |  |  |  |  |  | 1 (reference) | |  | |  |  | | |  |  | 1 (reference) |  |  |  | |  | |
|  | Frail to robust/pre-frail | 0.91 (0.68, 1.22) | 0.546 |  |  |  |  | 0.61 (0.31, 1.09) | | 0.118 | |  |  | | |  |  | 0.91 (0.55, 1.45) | 0.709 |  |  | |  | |
| **Executive function trajectories** | |  |  |  |  |  |  |  | |  | |  |  | | |  |  |  |  |  |  | |  | |
|  | Stable robust | 1 (reference) |  |  | 1 (reference) |  |  | 1 (reference) | |  | |  | 1 (reference) | | |  |  | 1 (reference) |  |  | 1 (reference) | |  | |
|  | Robust to pre-frail/frail | 1.64 (1.30, 2.07) | <0.001 |  | 1.21 (0.99, 1.47) | 0.060 |  | 1.21 (0.79, 1.87) | | 0.382 | |  | 1.48 (1.14, 1.93) | | | 0.003 |  | 1.59 (1.01, 2.51) | 0.046 |  | 1.31 (1.00, 1.71) | | 0.047 | |
|  | Stable pre-frail | 1 (reference) |  |  | 1 (reference) |  |  | 1 (reference) | |  | |  | 1 (reference) | | |  |  | 1 (reference) |  |  | 1 (reference) | |  | |
|  | Pre-frail to robust | 0.94 (0.69, 1.28) | 0.692 |  | 0.92 (0.70, 1.21) | 0.551 |  | 1.00 (0.59, 1.68) | | 0.992 | |  | 0.93 (0.69, 1.23) | | | 0.623 |  | 0.51 (0.24, 1.07) | 0.074 |  | 0.86 (0.60, 1.22) | | 0.399 | |
|  | Pre-frail to frail | 1.24 (0.91, 1.69) | 0.173 |  | 1.22 (0.93, 1.60) | 0.158 |  | 1.75 (1.14, 2.69) | | 0.010 | |  | 1.30 (0.92, 1.85) | | | 0.139 |  | 1.23 (0.84, 1.81) | 0.294 |  | 1.04 (0.82, 1.31) | | 0.764 | |
|  | Stable frail | 1 (reference) |  |  | 1 (reference) |  |  | 1 (reference) | |  | |  | 1 (reference) | | |  |  | 1 (reference) |  |  | 1 (reference) | |  | |
|  | Frail to robust/pre-frail | 0.86 (0.61, 1.20) | 0.370 |  | 0.99 (0.71, 1.39) | 0.973 |  | 0.66 (0.40, 1.09) | | 0.106 | |  | 0.86 (0.59, 1.26) | | | 0.450 |  | 0.87 (0.58, 1.31) | 0.507 |  | 0.84 (0.63, 1.11) | | 0.212 | |

****Notes.** Two-wave frailty change was defined from the preceding survey to the baseline survey. The alternative FI cut-off scheme defined robust as FI ≤ 0.10, pre-frail as 0.10 < FI ≤ 0.21, and frail as FI > 0.21. Stable robust, stable pre-frail, and stable frail participants served as the corresponding reference groups within each initial frailty-status stratum. For each cognitive outcome, the high-score trajectory served as the reference category. Models were adjusted for age, sex, education, marital status, smoking status, drinking status, and body mass index.**

****Abbreviations:** OR, odds ratio; CI, confidence interval; FI, frailty index; CHARLS, China Health and Retirement Longitudinal Study; ELSA, English Longitudinal Study of Ageing; HRS, Health and Retirement Study.**

**Table S20.** Association between baseline frailty status and cognitive trajectories after additional adjustment for baseline cognitive z-score

| **Frailty status** | | **CHARLS (n = 5876)** | | | | |  | **ELSA (n = 7134)** | | | | |  | **HRS (n = 6576)** | | | | |
| --- | --- | --- | --- | --- | --- | --- | --- | --- | --- | --- | --- | --- | --- | --- | --- | --- | --- | --- |
|  |  | **Low-score trajectory (vs high-score trajectory)** | |  | **Moderate-score trajectory (vs high-score trajectory)** | |  | **Low-score trajectory (vs high-score trajectory)** | |  | **Moderate-score trajectory (vs high-score trajectory)** | |  | **Low-score trajectory (vs high-score trajectory)** | |  | **Moderate-score trajectory (vs high-score trajectory)** | |
|  |  | **OR (95% CI)^*^** | ***p*** |  | **OR (95% CI)^*^** | ***p*** |  | **OR (95% CI)^*^** | ***p*** |  | **OR (95% CI)^*^** | ***p*** |  | **OR (95% CI)^*^** | ***p*** |  | **OR (95% CI)^*^** | ***p*** |
| Global cognitive trajectories | |  |  |  |  |  |  |  |  |  |  |  |  |  |  |  |  |  |
|  | Robust | 1 (reference) |  |  | 1 (reference) |  |  | 1 (reference) |  |  | 1 (reference) |  |  | 1 (reference) |  |  | 1 (reference) |  |
|  | Pre-frail | 1.58 (1.21, 2.05) | < 0.001 |  | 1.17 (1.00, 1.36) | < 0.05 |  | 1.11 (0.83, 1.47) | 0.482 |  | 1.13 (1.00, 1.29) | < 0.05 |  | 1.80 (1.42, 2.28) | < 0.001 |  | 1.55 (1.35, 1.78) | < 0.001 |
|  | Frail | 2.06 (1.44, 2.96) | < 0.001 |  | 1.54 (1.21, 1.96) | < 0.001 |  | 2.74 (2.09, 3.59) | < 0.001 |  | 1.64 (1.41, 1.90) | < 0.001 |  | 4.35 (3.41, 5.55) | < 0.001 |  | 2.17 (1.85, 2.55) | < 0.001 |
| Memory trajectories | |  |  |  |  |  |  |  |  |  |  |  |  |  |  |  |  |  |
|  | Robust | 1 (reference) |  |  | 1 (reference) |  |  | 1 (reference) |  |  | 1 (reference) |  |  | 1 (reference) |  |  | 1 (reference) |  |
|  | Pre-frail | 1.65 (1.29, 2.10) | < 0.001 |  | 1.37 (1.12, 1.68) | < 0.05 |  | 1.35 (1.08, 1.70) | < 0.05 |  | 1.23 (1.07, 1.42) | < 0.05 |  | 2.10 (1.68, 2.63) | < 0.001 |  | 1.61 (1.37, 1.89) | < 0.001 |
|  | Frail | 3.05 (2.01, 4.64) | < 0.001 |  | 2.13 (1.46, 3.11) | < 0.001 |  | 2.98 (2.34, 3.80) | < 0.001 |  | 1.59 (1.33, 1.89) | < 0.001 |  | 4.18 (3.26, 5.37) | < 0.001 |  | 2.10 (1.73, 2.55) | < 0.001 |
| Orientation trajectories | |  |  |  |  |  |  |  |  |  |  |  |  |  |  |  |  |  |
|  | Robust | 1 (reference) |  |  |  |  |  | 1 (reference) |  |  |  |  |  | 1 (reference) |  |  |  |  |
|  | Pre-frail | 1.12 (0.93, 1.35) | 0.221 |  |  |  |  | 1.14 (0.77, 1.69) | 0.517 |  |  |  |  | 1.31 (0.94, 1.85) | 0.118 |  |  |  |
|  | Frail | 1.49 (1.17, 1.90) | < 0.05 |  |  |  |  | 2.09 (1.45, 3.05) | < 0.001 |  |  |  |  | 2.38 (1.72, 3.34) | < 0.001 |  |  |  |
| Executive function trajectories | |  |  |  |  |  |  |  |  |  |  |  |  |  |  |  |  |  |
|  | Robust | 1 (reference) |  |  | 1 (reference) |  |  | 1 (reference) |  |  | 1 (reference) |  |  | 1 (reference) |  |  | 1 (reference) |  |
|  | Pre-frail | 1.49 (1.14, 1.95) | < 0.05 |  | 1.11 (0.95, 1.29) | 0.179 |  | 1.15 (0.84, 1.56) | 0.385 |  | 1.26 (1.03, 1.52) | < 0.05 |  | 1.46 (1.11, 1.92) | < 0.05 |  | 1.36 (1.17, 1.58) | < 0.001 |
|  | Frail | 1.89 (1.32, 2.72) | < 0.001 |  | 1.52 (1.22, 1.89) | < 0.001 |  | 2.25 (1.68, 3.00) | < 0.001 |  | 1.42 (1.15, 1.74) | < 0.001 |  | 2.68 (2.06, 3.49) | < 0.001 |  | 1.69 (1.44, 1.99) | < 0.001 |

**Notes.** Frailty status was measured at baseline and defined as robust, FI ≤ 0.10; pre-frail, 0.10 < FI < 0.25; and frail, FI ≥ 0.25. Robust participants served as the reference group. For each cognitive outcome, the high-score trajectory served as the reference category. Models were adjusted for age, sex, education, marital status, smoking status, drinking status, and body mass index, and baseline cognitive z-score. For global cognitive trajectories, models were adjusted for baseline global cognitive z-score; for domain-specific trajectories, models were adjusted for the corresponding baseline domain-specific cognitive z-score.

**Abbreviations:** BMI, body mass index; CI, confidence interval; CHARLS, China Health and Retirement Longitudinal Study; ELSA, English Longitudinal Study of Ageing; FI, frailty index; HRS, Health and Retirement Study; OR, odds ratio.

**Table S21.** Association between two-wave frailty change and cognitive trajectories after additional adjustment for baseline cognitive z-score

| **Two-wave frailty change** | | **CHARLS (n = 5876)** | | | | |  | **ELSA (n = 7134)** | | | | |  | **HRS (n = 6576)** | | | | |
| --- | --- | --- | --- | --- | --- | --- | --- | --- | --- | --- | --- | --- | --- | --- | --- | --- | --- | --- |
|  |  | **Low-score trajectory (vs high-score trajectory)** | |  | **Moderate-score trajectory (vs high-score trajectory)** | |  | **Low-score trajectory (vs high-score trajectory)** | |  | **Moderate-score trajectory (vs high-score trajectory)** | |  | **Low-score trajectory (vs high-score trajectory)** | |  | **Moderate-score trajectory (vs high-score trajectory)** | |
|  |  | **OR (95% CI)**^*^ | ***p*** |  | **OR (95% CI)**^*^ | ***p*** |  | **OR (95% CI)**^*^ | ***p*** |  | **OR (95% CI)**^*^ | ***p*** |  | **OR (95% CI)**^*^ | ***p*** |  | **OR (95% CI)**^*^ | ***p*** |
| **Global cognitive trajectories** | |  |  |  |  |  |  |  |  |  |  |  |  |  |  |  |  |  |
|  | Stable robust | 1 (reference) |  |  | 1 (reference) |  |  | 1 (reference) |  |  | 1 (reference) |  |  | 1 (reference) |  |  | 1 (reference) |  |
|  | Robust to pre-frail/frail | 2.17 (1.49, 3.18) | < 0.001 |  | 1.39 (1.11, 1.73) | < 0.01 |  | 1.04 (0.61, 1.77) | 0.885 |  | 1.19 (1.01, 1.43) | < 0.05 |  | 1.35 (0.75, 2.43) | 0.324 |  | 1.34 (1.00, 1.80) | < 0.05 |
|  | Stable pre-frail | 1 (reference) |  |  | 1 (reference) |  |  | 1 (reference) |  |  | 1 (reference) |  |  | 1 (reference) |  |  | 1 (reference) |  |
|  | Pre-frail to robust | 0.93 (0.59, 1.49) | 0.770 |  | 0.96 (0.72, 1.28) | 0.792 |  | 0.73 (0.38, 1.41) | 0.352 |  | 0.86 (0.63, 1.19) | 0.375 |  | 0.50 (0.22, 1.14) | 0.101 |  | 0.84 (0.57, 1.26) | 0.402 |
|  | Pre-frail to frail | 1.68 (1.04, 2.74) | < 0.05 |  | 1.49 (1.07, 2.08) | < 0.05 |  | 1.60 (1.06, 2.41) | <0.05 |  | 1.08 (0.78, 1.47) | 0.634 |  | 1.05 (0.63, 1.79) | 0.855 |  | 1.01 (0.76, 1.34) | 0.947 |
|  | Stable frail | 1 (reference) |  |  | 1 (reference) |  |  | 1 (reference) |  |  | 1 (reference) |  |  | 1 (reference) |  |  | 1 (reference) |  |
|  | Frail to robust/pre-frail | 0.92 (0.59, 1.44) | 0.719 |  | 0.97 (0.60, 1.57) | 0.901 |  | 0.90 (0.57, 1.41) | 0.640 |  | 0.82 (0.53, 1.25) | 0.359 |  | 0.97 (0.52, 1.81) | 0.922 |  | 0.94 (0.64, 1.40) | 0.776 |
| **Memory trajectories** | |  |  |  |  |  |  |  |  |  |  |  |  |  |  |  |  |  |
|  | Stable robust | 1 (reference) |  |  | 1 (reference) |  |  | 1 (reference) |  |  | 1 (reference) |  |  | 1 (reference) |  |  | 1 (reference) |  |
|  | Robust to pre-frail/frail | 1.79 (1.27, 2.51) | < 0.001 |  | 1.32 (1.00, 1.75) | < 0.05 |  | 1.59 (1.00, 2.52) | < 0.05 |  | 1.31 (0.96, 1.79) | 0.083 |  | 1.21 (0.78, 1.81) | 0.319 |  | 1.28 (0.91, 1.78) | 0.154 |
|  | Stable pre-frail | 1 (reference) |  |  | 1 (reference) |  |  | 1 (reference) |  |  | 1 (reference) |  |  | 1 (reference) |  |  | 1 (reference) |  |
|  | Pre-frail to robust | 0.66 (0.43, 1.01) | 0.051 |  | 0.66 (0.46, 0.94) | < 0.05 |  | 0.95 (0.55, 1.65) | 0.858 |  | 0.80 (0.55, 1.16) | 0.239 |  | 0.43 (0.22, 0.84) | <0.05 |  | 0.49 (0.31, 0.76) | <0.01 |
|  | Pre-frail to frail | 2.26 (1.26, 4.06) | < 0.01 |  | 1.80 (1.06, 3.06) | < 0.05 |  | 1.28 (0.80, 1.99) | 0.145 |  | 1.00 (0.69, 1.40) | 0.731 |  | 1.08 (0.56, 2.08) | 0.818 |  | 1.01 (0.69, 1.46) | 0.958 |
|  | Stable frail | 1 (reference) |  |  | 1 (reference) |  |  | 1 (reference) |  |  | 1 (reference) |  |  | 1 (reference) |  |  | 1 (reference) |  |
|  | Frail to robust/pre-frail | 0.75 (0.33, 1.69) | 0.483 |  | 0.69 (0.33, 1.46) | 0.335 |  | 0.93 (0.44, 1.95) | 0.746 |  | 0.74 (0.41, 1.33) | 0.310 |  | 0.99 (0.58, 1.67) | 0.672 |  | 0.79 (0.50, 1.27) | 0.331 |
| **Orientation trajectories** | |  |  |  |  |  |  |  |  |  |  |  |  |  |  |  |  |  |
|  | Stable robust | 1 (reference) |  |  |  |  |  | 1 (reference) |  |  |  |  |  | 1 (reference) |  |  |  |  |
|  | Robust to pre-frail/frail | 1.15 (0.88, 1.49) | 0.304 |  |  |  |  | 1.02 (0.60, 1.76) | 0.942 |  |  |  |  | 1.23 (0.65, 2.23) | 0.507 |  |  |  |
|  | Stable pre-frail | 1 (reference) |  |  |  |  |  | 1 (reference) |  |  |  |  |  | 1 (reference) |  |  |  |  |
|  | Pre-frail to robust | 0.92 (0.66, 1.27) | 0.604 |  |  |  |  | 0.56 (0.25, 1.13) | 0.129 |  |  |  |  | 0.45 (0.15, 1.08) | 0.108 |  |  |  |
|  | Pre-frail to frail | 1.51 (1.09, 2.10) | < 0.05 |  |  |  |  | 1.08 (0.60, 1.90) | 0.701 |  |  |  |  | 1.41 (0.92, 2.12) | 0.107 |  |  |  |
|  | Stable frail | 1 (reference) |  |  |  |  |  | 1 (reference) |  |  |  |  |  | 1 (reference) |  |  |  |  |
|  | Frail to robust/pre-frail | 0.90 (0.64, 1.28) | 0.561 |  |  |  |  | 0.96 (0.51, 1.76) | 0.897 |  |  |  |  | 0.74 (0.51, 1.08) | 0.214 |  |  |  |
| **Executive function trajectories** | |  |  |  |  |  |  |  |  |  |  |  |  |  |  |  |  |  |
|  | Stable robust | 1 (reference) |  |  | 1 (reference) |  |  | 1 (reference) |  |  | 1 (reference) |  |  | 1 (reference) |  |  | 1 (reference) |  |
|  | Robust to pre-frail/frail | 2.27 (1.56, 3.30) | <0.001 |  | 1.20 (0.97, 1.49) | 0.088 |  | 1.08 (0.56, 2.08) | 0.822 |  | 1.39 (1.04, 1.86) | < 0.05 |  | 1.39 (0.69, 2.78) | 0.352 |  | 1.15 (0.82, 1.62) | 0.342 |
|  | Stable pre-frail | 1 (reference) |  |  | 1 (reference) |  |  | 1 (reference) |  |  | 1 (reference) |  |  | 1 (reference) |  |  | 1 (reference) |  |
|  | Pre-frail to robust | 0.86 (0.54, 1.39) | 0.540 |  | 0.95 (0.72, 1.25) | 0.717 |  | 0.58 (0.25, 1.32) | 0.194 |  | 0.94 (0.68, 1.26) | 0.726 |  | 0.42 (0.16, 1.07) | 0.058 |  | 0.61 (0.37, 1.03) | 0.064 |
|  | Pre-frail to frail | 1.34 (0.82, 2.20) | 0.240 |  | 1.34 (1.00, 1.81) | <0.05 |  | 1.27 (0.78, 1.97) | 0.368 |  | 1.00 (0.70, 1.42) | 0.660 |  | 1.11 (0.64, 1.92) | 0.706 |  | 1.02 (0.76, 1.36) | 0.790 |
|  | Stable frail | 1 (reference) |  |  | 1 (reference) |  |  | 1 (reference) |  |  | 1 (reference) |  |  | 1 (reference) |  |  | 1 (reference) |  |
|  | Frail to robust/pre-frail | 0.68 (0.35, 1.35) | 0.271 |  | 0.71 (0.46, 1.10) | 0.128 |  | 0.75 (0.45, 1.22) | 0.284 |  | 1.00 (0.67, 1.44) | 0.938 |  | 0.83 (0.40, 1.72) | 0.619 |  | 0.71 (0.48, 1.06) | 0.096 |

**Notes.** Two-wave frailty change was defined from the preceding survey to the baseline survey. Stable robust, stable pre-frail, and stable frail participants served as the corresponding reference groups within each preceding frailty-status stratum. For each cognitive outcome, the high-score trajectory served as the reference category. Models were adjusted for age, sex, education, marital status, smoking status, drinking status, and body mass index, and baseline cognitive z-score. For global cognitive trajectories, models were adjusted for baseline global cognitive z-score; for domain-specific trajectories, models were adjusted for the corresponding baseline domain-specific cognitive z-score.

**Abbreviations:** BMI, body mass index; CI, confidence interval; CHARLS, China Health and Retirement Longitudinal Study; ELSA, English Longitudinal Study of Ageing; FI, frailty index; HRS, Health and Retirement Study; OR, odds ratio.

**Table S22.** Association between the mean frailty burden and cognitive trajectories after additional adjustment for baseline cognitive z-score

| **Mean frailty burden** | | **CHARLS (n = 5876)** | | | | |  | **ELSA (n = 7134)** | | | | |  | **HRS (n = 6576)** | | | | |
| --- | --- | --- | --- | --- | --- | --- | --- | --- | --- | --- | --- | --- | --- | --- | --- | --- | --- | --- |
|  |  | **Low-score trajectory (vs high-score trajectory)** | |  | **Moderate-score trajectory (vs high-score trajectory)** | |  | **Low-score trajectory (vs high-score trajectory)** | |  | **Moderate-score trajectory (vs high-score trajectory)** | |  | **Low-score trajectory (vs high-score trajectory)** | |  | **Moderate-score trajectory (vs high-score trajectory)** | |
|  |  | **OR (95% CI)**^*^ | ***p*** |  | **OR (95% CI)**^*^ | ***p*** |  | **OR (95% CI)**^*^ | ***p*** |  | **OR (95% CI)**^*^ | ***p*** |  | **OR (95% CI)**^*^ | ***p*** |  | **OR (95% CI)**^*^ | ***p*** |
| **Global cognitive trajectories** | |  |  |  |  |  |  |  |  |  |  |  |  |  |  |  |  |  |
|  | T1 | 1 (reference) |  |  | 1 (reference) |  |  | 1 (reference) |  |  | 1 (reference) |  |  | 1 (reference) |  |  | 1 (reference) |  |
|  | T2 | 1.49 (1.09, 2.04) | < 0.05 |  | 1.21 (1.02, 1.44) | < 0.05 |  | 1.07 (0.82, 1.33) | 0.576 |  | 1.02 (0.86, 1.21) | 0.826 |  | 1.53 (1.09, 2.14) | < 0.05 |  | 1.39 (1.17, 1.65) | < 0.001 |
|  | T3 | 1.92 (1.41, 2.61) | < 0.001 |  | 1.32 (1.10, 1.60) | < 0.05 |  | 1.62 (1.11, 2.35) | < 0.05 |  | 1.26 (1.03, 1.53) | < 0.05 |  | 2.36 (1.66, 3.35) | < 0.001 |  | 1.52 (1.24, 1.86) | < 0.001 |
|  | *P for trend* | < 0.001 |  |  | < 0.001 |  |  | < 0.001 |  |  | < 0.001 |  |  | < 0.001 |  |  | < 0.001 |  |
| **Memory trajectories** | |  |  |  |  |  |  |  |  |  |  |  |  |  |  |  |  |  |
|  | T1 | 1 (reference) |  |  | 1 (reference) |  |  | 1 (reference) |  |  | 1 (reference) |  |  | 1 (reference) |  |  | 1 (reference) |  |
|  | T2 | 1.21 (0.93, 1.58) | 0.151 |  | 1.09 (0.89, 1.35) | 0.410 |  | 1.25 (0.91, 1.71) | 0.170 |  | 1.27 (1.05, 1.54) | < 0.05 |  | 1.93 (1.42, 2.61) | < 0.001 |  | 1.47 (1.20, 1.80) | < 0.001 |
|  | T3 | 1.91 (1.43, 2.57) | < 0.001 |  | 1.37 (1.06, 1.75) | < 0.05 |  | 2.26 (1.50, 3.18) | < 0.001 |  | 1.49 (1.17, 1.89) | < 0.05 |  | 2.70 (1.92, 3.80) | < 0.001 |  | 1.64 (1.28, 2.11) | < 0.001 |
|  | *P for trend* | <0.001 |  |  | <0.001 |  |  | <0.001 |  |  | <0.001 |  |  | <0.001 |  |  | <0.001 |  |
| **Orientation trajectories** | |  |  |  |  |  |  |  |  |  |  |  |  |  |  |  |  |  |
|  | T1 | 1 (reference) |  |  |  |  |  | 1 (reference) |  |  |  |  |  | 1 (reference) |  |  |  |  |
|  | T2 | 1.06 (0.85, 1.31) | 0.624 |  |  |  |  | 1.13 (0.75, 1.72) | 0.558 |  |  |  |  | 1.30 (0.92, 1.85) | 0.134 |  |  |  |
|  | T3 | 1.33 (1.07, 1.64) | < 0.05 |  |  |  |  | 1.98 (1.34, 2.96) | < 0.001 |  |  |  |  | 2.15 (1.54, 3.04) | < 0.001 |  |  |  |
|  | *P for trend* | < 0.001 |  |  |  |  |  | < 0.001 |  |  |  |  |  | < 0.001 |  |  |  |  |
| **Executive function trajectories** | |  |  |  |  |  |  |  |  |  |  |  |  |  |  |  |  |  |
|  | T1 | 1 (reference) |  |  | 1 (reference) |  |  | 1 (reference) |  |  | 1 (reference) |  |  | 1 (reference) |  |  | 1 (reference) |  |
|  | T2 | 1.73 (1.26, 2.36) | < 0.001 |  | 1.12 (0.95, 1.33) | 0.172 |  | 1.24 (0.77, 1.98) | 0.376 |  | 1.28 (1.03, 1.59) | < 0.05 |  | 1.58 (1.05, 2.37) | < 0.05 |  | 1.39 (1.14, 1.70) | < 0.05 |
|  | T3 | 1.63 (1.19, 2.22) | < 0.05 |  | 1.33 (1.12, 1.59) | < 0.05 |  | 2.26 (1.43, 3.57) | < 0.001 |  | 1.39 (1.11, 1.75) | < 0.05 |  | 2.46 (1.65, 3.67) | < 0.001 |  | 1.61 (1.30, 1.99) | < 0.001 |
|  | *P for trend* | < 0.001 |  |  | < 0.001 |  |  | < 0.001 |  |  | < 0.05 |  |  | < 0.001 |  |  | < 0.001 |  |

**Notes.** The mean frailty burden was calculated as the mean of the FI measured at the preceding survey and the baseline survey. Participants were categorized into cohort-specific tertiles of the mean frailty burden, with T1 representing the lowest tertile and serving as the reference group. T2 and T3 represented the middle and highest tertiles, respectively. For each cognitive outcome, the high-score trajectory served as the reference category. Models were adjusted for age, sex, education, marital status, smoking status, drinking status, and body mass index, and baseline cognitive z-score. For global cognitive trajectories, models were adjusted for baseline global cognitive z-score; for domain-specific trajectories, models were adjusted for the corresponding baseline domain-specific cognitive z-score. P for trend was calculated by treating FI tertiles as an ordinal variable.

**Abbreviations:** BMI, body mass index; CI, confidence interval; CHARLS, China Health and Retirement Longitudinal Study; ELSA, English Longitudinal Study of Ageing; FI, frailty index; HRS, Health and Retirement Study; OR, odds ratio.

**Table S23.** Lagged-exposure sensitivity analysis of preceding-wave frailty status and cognitive trajectories with adjustment for cognitive z-scores at the preceding survey

| **Frailty status** | | **CHARLS (n = 5876)** | | | | |  | **ELSA (n = 7134)** | | | | |  | **HRS (n = 6576)** | | | | |
| --- | --- | --- | --- | --- | --- | --- | --- | --- | --- | --- | --- | --- | --- | --- | --- | --- | --- | --- |
|  |  | **Low-score trajectory (vs high-score trajectory)** | |  | **Moderate-score trajectory (vs high-score trajectory)** | |  | **Low-score trajectory (vs high-score trajectory)** | |  | **Moderate-score trajectory (vs high-score trajectory)** | |  | **Low-score trajectory (vs high-score trajectory)** | |  | **Moderate-score trajectory (vs high-score trajectory)** | |
|  |  | **OR (95% CI)^*^** | ***p*** |  | **OR (95% CI)^*^** | ***p*** |  | **OR (95% CI)^*^** | ***p*** |  | **OR (95% CI)^*^** | ***p*** |  | **OR (95% CI)^*^** | ***p*** |  | **OR (95% CI)^*^** | ***p*** |
| Global cognitive trajectories | |  |  |  |  |  |  |  |  |  |  |  |  |  |  |  |  |  |
|  | Robust | 1 (reference) |  |  | 1 (reference) |  |  | 1 (reference) |  |  | 1 (reference) |  |  | 1 (reference) |  |  | 1 (reference) |  |
|  | Pre-frail | 1.46 (1.23, 1.73) | < 0.001 |  | 1.31 (1.14, 1.49) | < 0.001 |  | 1.59 (1.22, 2.07) | < 0.001 |  | 1.15 (1.00, 1.32) | < 0.05 |  | 1.53 (1.15, 2.04) | < 0.01 |  | 1.38 (1.18, 1.60) | < 0.001 |
|  | Frail | 2.46 (1.91, 3.17) | < 0.001 |  | 1.61 (1.28, 2.01) | < 0.001 |  | 2.60 (1.92, 3.52) | < 0.001 |  | 1.59 (1.32, 1.91) | < 0.001 |  | 3.03 (2.24, 4.11) | < 0.001 |  | 1.83 (1.53, 2.20) | < 0.001 |
| Memory trajectories | |  |  |  |  |  |  |  |  |  |  |  |  |  |  |  |  |  |
|  | Robust | 1 (reference) |  |  | 1 (reference) |  |  | 1 (reference) |  |  | 1 (reference) |  |  | 1 (reference) |  |  | 1 (reference) |  |
|  | Pre-frail | 1.70 (1.40, 2.06) | < 0.001 |  | 1.46 (1.22, 1.73) | < 0.001 |  | 1.73 (1.37, 2.19) | < 0.001 |  | 1.17 (1.01, 1.35) | < 0.05 |  | 1.82 (1.42, 2.34) | < 0.001 |  | 1.33 (1.13, 1.57) | < 0.001 |
|  | Frail | 2.34 (1.66, 3.30) | < 0.001 |  | 1.48 (1.06, 2.05) | < 0.05 |  | 2.87 (2.10, 3.94) | < 0.001 |  | 1.73 (1.35, 2.21) | < 0.001 |  | 3.60 (2.69, 4.81) | < 0.001 |  | 1.84 (1.49, 2.27) | < 0.001 |
| Orientation trajectories | |  |  |  |  |  |  |  |  |  |  |  |  |  |  |  |  |  |
|  | Robust | 1 (reference) |  |  |  |  |  | 1 (reference) |  |  |  |  |  | 1 (reference) |  |  |  |  |
|  | Pre-frail | 1.18 (1.03, 1.37) | < 0.05 |  |  |  |  | 1.39 (1.01, 2.01) | < 0.05 |  |  |  |  | 1.33 (0.95, 1.89) | 0.107 |  |  |  |
|  | Frail | 1.74 (1.42, 2.13) | < 0.001 |  |  |  |  | 2.37 (1.66, 3.41) | < 0.001 |  |  |  |  | 2.07 (1.47, 2.97) | < 0.001 |  |  |  |
| Executive function trajectories | |  |  |  |  |  |  |  |  |  |  |  |  |  |  |  |  |  |
|  | Robust | 1 (reference) |  |  | 1 (reference) |  |  | 1 (reference) |  |  | 1 (reference) |  |  | 1 (reference) |  |  | 1 (reference) |  |
|  | Pre-frail | 1.25 (1.07, 1.47) | < 0.01 |  | 1.17 (1.02, 1.34) | < 0.05 |  | 1.63 (1.25, 2.13) | < 0.01 |  | 1.13 (0.95, 1.34) | 0.191 |  | 1.01 (0.73, 1.40) | 0.996 |  | 1.19 (1.00, 1.39) | < 0.05 |
|  | Frail | 1.91 (1.51, 2.41) | < 0.001 |  | 1.36 (1.09, 1.70) | < 0.01 |  | 1.85 (1.38, 2.48) | < 0.001 |  | 1.24 (1.00, 1.53) | < 0.05 |  | 1.63 (1.16, 2.29) | < 0.05 |  | 1.34 (1.11, 1.63) | < 0.01 |

**Notes.** Frailty status was measured at the preceding survey and defined as robust, FI ≤ 0.10; pre-frail, 0.10 < FI < 0.25; and frail, FI ≥ 0.25. Robust participants served as the reference group. Cognitive trajectories were identified from the baseline survey onward. For each cognitive outcome, the high-score trajectory served as the reference category. Models were adjusted for age, sex, education, marital status, smoking status, drinking status, and body mass index, and cognitive z-score measured at the preceding survey. For global cognitive trajectories, models were adjusted for preceding-wave global cognitive z-score; for domain-specific trajectories, models were adjusted for the corresponding preceding-wave domain-specific cognitive z-score.

**Abbreviations:** BMI, body mass index; CI, confidence interval; CHARLS, China Health and Retirement Longitudinal Study; ELSA, English Longitudinal Study of Ageing; FI, frailty index; HRS, Health and Retirement Study; OR, odds ratio.

**Table S24**. Lagged-exposure sensitivity analysis of preceding-wave frailty index tertiles and cognitive trajectories with adjustment for cognitive z-scores at the preceding survey

| **Preceding wave frailty index tertiles** | | **CHARLS (n = 5876)** | | | | |  | **ELSA (n = 7134)** | | | | |  | **HRS (n = 6576)** | | | | |
| --- | --- | --- | --- | --- | --- | --- | --- | --- | --- | --- | --- | --- | --- | --- | --- | --- | --- | --- |
|  |  | **Low-score trajectory (vs high-score trajectory)** | |  | **Moderate-score trajectory (vs high-score trajectory)** | |  | **Low-score trajectory (vs high-score trajectory)** | |  | **Moderate-score trajectory (vs high-score trajectory)** | |  | **Low-score trajectory (vs high-score trajectory)** | |  | **Moderate-score trajectory (vs high-score trajectory)** | |
|  |  | **OR (95% CI)**^*^ | ***p*** |  | **OR (95% CI)**^*^ | ***p*** |  | **OR (95% CI)**^*^ | ***p*** |  | **OR (95% CI)**^*^ | ***p*** |  | **OR (95% CI)**^*^ | ***p*** |  | **OR (95% CI)**^*^ | ***p*** |
| **Global cognitive trajectories** | |  |  |  |  |  |  |  |  |  |  |  |  |  |  |  |  |  |
|  | T1 | 1 (reference) |  |  | 1 (reference) |  |  | 1 (reference) |  |  | 1 (reference) |  |  | 1 (reference) |  |  | 1 (reference) |  |
|  | T2 | 1.33 (1.09, 1.64) | < 0.01 |  | 1.19 (1.03, 1.37) | < 0.05 |  | 1.16 (0.85, 1.58) | 0.347 |  | 1.15 (1.00, 1.29) | < 0.05 |  | 1.49 (1.12, 1.97) | < 0.01 |  | 1.32 (1.13, 1.53) | < 0.001 |
|  | T3 | 2.22 (1.81, 2.70) | < 0.001 |  | 1.57 (1.34, 1.83) | < 0.001 |  | 2.18 (1.61, 2.95) | < 0.001 |  | 1.43 (1.22, 1.67) | < 0.001 |  | 2.63 (1.99, 3.47) | < 0.001 |  | 1.68 (1.42, 1.99) | < 0.001 |
|  | *P for trend* | < 0.001 |  |  | < 0.001 |  |  | < 0.001 |  |  | < 0.001 |  |  | < 0.001 |  |  | < 0.001 |  |
| **Memory trajectories** | |  |  |  |  |  |  |  |  |  |  |  |  |  |  |  |  |  |
|  | T1 | 1 (reference) |  |  | 1 (reference) |  |  | 1 (reference) |  |  | 1 (reference) |  |  | 1 (reference) |  |  | 1 (reference) |  |
|  | T2 | 1.53 (1.24, 1.88) | < 0.001 |  | 1.26 (1.05, 1.51) | < 0.05 |  | 1.53 (1.18, 1.98) | < 0.01 |  | 1.24 (1.10, 1.46) | < 0.01 |  | 1.61 (1.26, 2.06) | < 0.001 |  | 1.30 (1.11, 1.52) | < 0.001 |
|  | T3 | 2.28 (1.82, 2.86) | < 0.001 |  | 1.57 (1.28, 1.93) | < 0.001 |  | 2.49 (1.89, 3.27) | < 0.001 |  | 1.49 (1.24, 1.80) | < 0.001 |  | 3.24 (2.48, 4.22) | < 0.001 |  | 1.76 (1.45, 2.14) | < 0.001 |
|  | *P for trend* | <0.001 |  |  | <0.001 |  |  | <0.001 |  |  | <0.001 |  |  | <0.001 |  |  | <0.001 |  |
| **Orientation trajectories** | |  |  |  |  |  |  |  |  |  |  |  |  |  |  |  |  |  |
|  | T1 | 1 (reference) |  |  |  |  |  | 1 (reference) |  |  |  |  |  | 1 (reference) |  |  |  |  |
|  | T2 | 1.10 (0.93, 1.31) | 0.268 |  |  |  |  | 1.18 (0.80, 1.77) | 0.411 |  |  |  |  | 1.37 (0.98, 1.93) | 0.068 |  |  |  |
|  | T3 | 1.50 (1.28, 1.77) | < 0.001 |  |  |  |  | 1.87 (1.29, 2.76) | < 0.001 |  |  |  |  | 2.07 (1.50, 2.88) | < 0.001 |  |  |  |
|  | *P for trend* | < 0.001 |  |  |  |  |  | < 0.001 |  |  |  |  |  | < 0.001 |  |  |  |  |
| **Executive function trajectories** | |  |  |  |  |  |  |  |  |  |  |  |  |  |  |  |  |  |
|  | T1 | 1 (reference) |  |  | 1 (reference) |  |  | 1 (reference) |  |  | 1 (reference) |  |  | 1 (reference) |  |  | 1 (reference) |  |
|  | T2 | 1.18 (0.98, 1.44) | 0.083 |  | 1.05 (0.90, 1.22) | 0.531 |  | 1.23 (0.90, 1.69) | 0.197 |  | 1.30 (1.07, 1.59) | < 0.01 |  | 1.03 (0.78, 1.37) | 0.735 |  | 1.16 (0.98, 1.38) | 0.093 |
|  | T3 | 1.64 (1.36, 1.98) | < 0.001 |  | 1.32 (1.13, 1.55) | < 0.001 |  | 1.88 (1.39, 2.55) | < 0.001 |  | 1.34 (1.09, 1.64) | < 0.01 |  | 1.47 (1.07, 2.01) | < 0.05 |  | 1.30 (1.09, 1.55) | < 0.01 |
|  | *P for trend* | < 0.001 |  |  | < 0.001 |  |  | < 0.001 |  |  | < 0.001 |  |  | < 0.001 |  |  | < 0.001 |  |

**Notes.** Participants were categorized into cohort-specific tertiles of preceding-wave FI, with T1 representing the lowest tertile and serving as the reference group. T2 and T3 represented the middle and highest tertiles, respectively. Cognitive trajectories were identified from the baseline survey onward. For each cognitive outcome, the high-score trajectory served as the reference category. Models were adjusted for age, sex, education, marital status, smoking status, drinking status, and body mass index, and cognitive z-score measured at the preceding survey. For global cognitive trajectories, models were adjusted for preceding-wave global cognitive z-score; for domain-specific trajectories, models were adjusted for the corresponding preceding-wave domain-specific cognitive z-score. *P* for trend was calculated by treating FI tertiles as an ordinal variable.

**Abbreviations:** BMI, body mass index; CI, confidence interval; CHARLS, China Health and Retirement Longitudinal Study; ELSA, English Longitudinal Study of Ageing; FI, frailty index; HRS, Health and Retirement Study; OR, odds ratio.

**Table S25.** Baseline characteristics of participants included in and excluded from the main analyses

| **Characteristic** | **CHARLS** | |  | **ELSA** | |  | **HRS** | |
| --- | --- | --- | --- | --- | --- | --- | --- | --- |
|  | **Excluded participants** | **Included participants** |  | **Excluded participants** | **Included participants** |  | **Excluded participants** | **Included participants** |
| **Number, n** | 12579 | 5876 |  | 12668 | 7134 |  | 13127 | 6576 |
| **Age, mean (SD), years** | 59.5 (11.1) | 59.3 (8.2) |  | 66.6 (13.3) | 67.6 (8.8) |  | 65.1 (12.1) | 73.0 (7.0) |
| Missing | 113 (0.9) | 0 (0.0) |  | 8241 (65.1) | 0 (0.0) |  | 956 (7.3) | 0 (0.0) |
| **Sex, n (%)** |  |  |  |  |  |  |  |  |
| Female | 7142 (56.8) | 2502 (42.6) |  | 6774 (53.5) | 4016 (56.3) |  | 7532 (57.4) | 3973 (60.4) |
| Male | 5437 (43.2) | 3374 (57.4) |  | 5894 (46.5) | 3118 (43.7) |  | 5595 (42.6) | 2603 (39.6) |
| **Marital status, n (%)** |  |  |  |  |  |  |  |  |
| Married or partnered | 10728 (85.3) | 5307 (90.3) |  | 1643 (13.0) | 4787 (67.1) |  | 6881 (52.4) | 3741 (56.9) |
| Other marital status | 1844 (14.7) | 565 (9.6) |  | 888 (7.0) | 2346 (32.9) |  | 5290 (40.3) | 2835 (43.1) |
| Missing | 7 (0.1) | 4 (0.1) |  | 10137 (80.0) | 1 (0.0) |  | 956 (7.3) | 0 (0.0) |
| **Education, n (%)** |  |  |  |  |  |  |  |  |
| Below high school | 11120 (88.4) | 5051 (86.0) |  | 4864 (38.4) | 1689 (23.7) |  | 2653 (20.2) | 1031 (15.7) |
| High school | 1193 (9.5) | 715 (12.2) |  | 4981 (39.3) | 3482 (48.8) |  | 7666 (58.4) | 3895 (59.2) |
| College or above | 261 (2.1) | 110 (1.9) |  | 1531 (12.1) | 1432 (20.1) |  | 2801 (21.3) | 1647 (25.0) |
| Missing | 5 (0.0) | 0 (0.0) |  | 1292 (10.2) | 531 (7.4) |  | 7 (0.1) | 3 (0.0) |
| **Smoking status, n (%)** |  |  |  |  |  |  |  |  |
| Ever smokers | 4839 (38.5) | 2958 (50.3) |  | 1481 (11.7) | 4418 (61.9) |  | 6814 (51.9) | 3577 (54.4) |
| Never smokers | 7692 (61.1) | 2918 (49.7) |  | 871 (6.9) | 2715 (38.1) |  | 5299 (40.4) | 2952 (44.9) |
| Missing | 48 (0.4) | 0 (0.0) |  | 10316 (81.4) | 1 (0.0) |  | 1014 (7.7) | 47 (0.7) |
| **Drinking status, n (%)** |  |  |  |  |  |  |  |  |
| Ever drinkers | 4906 (39.0) | 3349 (57.0) |  | 1278 (10.1) | 5694 (79.8) |  | 6513 (49.6) | 3523 (53.6) |
| Never drinkers | 7585 (60.3) | 2521 (42.9) |  | 242 (1.9) | 817 (11.5) |  | 5654 (43.1) | 3053 (46.4) |
| Missing | 88 (0.7) | 6 (0.1) |  | 11148 (88.0) | 623 (8.7) |  | 960 (7.3) | 0 (0.0) |
| **BMI, mean (SD), kg/m2** | 23.9 (3.9) | 23.9 (3.7) |  | 28.0 (5.5) | 28.4 (5.2) |  | 30.1 (6.4) | 29.7 (5.9) |
| Missing | 4326 (34.4) | 1194 (20.3) |  | 10909 (86.1) | 1200 (16.8) |  | 5200 (39.6) | 1034 (15.7) |
| **Baseline frailty status, n (%)** |  |  |  |  |  |  |  |  |
| Robust | 3134 (24.9) | 2757 (46.9) |  | 1798 (14.2) | 3496 (49.0) |  | 3213 (24.5) | 1593 (24.2) |
| Pre-frail | 2800 (22.3) | 2328 (39.6) |  | 991 (7.8) | 2234 (31.3) |  | 2908 (22.2) | 2719 (41.3) |
| Frail | 1498 (11.9) | 791 (13.5) |  | 1167 (9.2) | 1404 (19.7) |  | 3006 (22.9) | 2264 (34.4) |
| Missing | 5147 (40.9) | 0 (0.0) |  | 8712 (68.8) | 0 (0.0) |  | 4000 (30.5) | 0 (0.0) |
| **Global cognition, mean (SD)** | 13.6 (6.4) | 15.5 (5.3) |  | 18.1 (6.7) | 20.8 (4.4) |  | 15.9 (4.7) | 18.5 (4.5) |
| Missing | 1836 (14.6) | 0 (0.0) |  | 10744 (84.8) | 0 (0.0) |  | 2005 (15.3) | 0 (0.0) |

**Notes.** Values are mean (SD) for continuous variables and n (%) for categorical variables. Included participants were those in the final analytic sample; excluded participants were baseline survey participants who were not included in the final analytic sample because of age criteria, missing data, insufficient follow-up cognitive assessments, loss to follow-up, death, or other exclusion criteria. Values are based on available baseline data without imputation. Missing values are shown for variables with incomplete baseline measurements.

**Abbreviations:** BMI, body mass index; CHARLS, China Health and Retirement Longitudinal Study; ELSA, English Longitudinal Study of Ageing; FI, frailty index; HRS, Health and Retirement Study; SD, standard deviation.

**Table S26.** Association between baseline frailty status and cognitive trajectories using inverse probability weighting

| **Frailty status** | | **CHARLS (n = 5876)** | | | | |  | **ELSA (n = 7134)** | | | | |  | **HRS (n = 6576)** | | | | |
| --- | --- | --- | --- | --- | --- | --- | --- | --- | --- | --- | --- | --- | --- | --- | --- | --- | --- | --- |
|  |  | **Low-score trajectory (vs high-score trajectory)** | |  | **Moderate-score trajectory (vs high-score trajectory)** | |  | **Low-score trajectory (vs high-score trajectory)** | |  | **Moderate-score trajectory (vs high-score trajectory)** | |  | **Low-score trajectory (vs high-score trajectory)** | |  | **Moderate-score trajectory (vs high-score trajectory)** | |
|  |  | **OR (95% CI)^*^** | ***p*** |  | **OR (95% CI)^*^** | ***p*** |  | **OR (95% CI)^*^** | ***p*** |  | **OR (95% CI)^*^** | ***p*** |  | **OR (95% CI)^*^** | ***p*** |  | **OR (95% CI)^*^** | ***p*** |
| Global cognitive trajectories | |  |  |  |  |  |  |  |  |  |  |  |  |  |  |  |  |  |
|  | Robust | 1 (reference) |  |  | 1 (reference) |  |  | 1 (reference) |  |  | 1 (reference) |  |  | 1 (reference) |  |  | 1 (reference) |  |
|  | Pre-frail | 1.54 (1.31, 1.82) | <0.001 |  | 1.25 (1.09, 1.44) | <0.05 |  | 1.49 (1.25, 1.77) | <0.001 |  | 1.12 (0.97, 1.28) | 0.127 |  | 1.70 (1.33, 2.17) | <0.001 |  | 1.47 (1.25, 1.74) | <0.001 |
|  | Frail | 3.05 (2.46, 3.79) | <0.001 |  | 2.02 (1.66, 2.46) | <0.001 |  | 6.82 (5.74, 8.11) | <0.001 |  | 2.65 (2.27, 3.09) | <0.001 |  | 5.63 (4.44, 7.15) | <0.001 |  | 2.63 (2.21, 3.14) | <0.001 |
| Memory trajectories | |  |  |  |  |  |  |  |  |  |  |  |  |  |  |  |  |  |
|  | Robust | 1 (reference) |  |  | 1 (reference) |  |  | 1 (reference) |  |  | 1 (reference) |  |  | 1 (reference) |  |  | 1 (reference) |  |
|  | Pre-frail | 1.77 (1.45, 2.17) | <0.001 |  | 1.52 (1.26, 1.83) | <0.001 |  | 1.46 (1.19, 1.79) | <0.001 |  | 1.15 (0.95, 1.38) | 0.148 |  | 2.03 (1.57, 2.62) | <0.001 |  | 1.74 (1.43, 2.11) | <0.001 |
|  | Frail | 3.76 (2.72, 5.19) | <0.001 |  | 2.39 (1.75, 3.27) | <0.001 |  | 6.37 (4.99, 8.12) | <0.001 |  | 2.73 (2.17, 3.45) | <0.001 |  | 5.65 (4.35, 7.33) | <0.001 |  | 2.71 (2.19, 3.35) | <0.001 |
| Orientation trajectories | |  |  |  |  |  |  |  |  |  |  |  |  |  |  |  |  |  |
|  | Robust | 1 (reference) |  |  |  |  |  | 1 (reference) |  |  |  |  |  | 1 (reference) |  |  |  |  |
|  | Pre-frail | 1.18 (1.03, 1.36) | <0.05 |  |  |  |  | 1.12 (0.90, 1.42) | 0.331 |  |  |  |  | 1.13 (0.81, 1.56) | 0.475 |  |  |  |
|  | Frail | 2.03 (1.72, 2.41) | <0.001 |  |  |  |  | 3.31 (2.79, 3.93) | <0.001 |  |  |  |  | 2.12 (1.57, 2.85) | <0.001 |  |  |  |
| Executive function trajectories | |  |  |  |  |  |  |  |  |  |  |  |  |  |  |  |  |  |
|  | Robust | 1 (reference) |  |  | 1 (reference) |  |  | 1 (reference) |  |  | 1 (reference) |  |  | 1 (reference) |  |  | 1 (reference) |  |
|  | Pre-frail | 1.39 (1.19, 1.62) | <0.001 |  | 1.13 (0.98, 1.30) | 0.104 |  | 1.42 (1.21, 1.67) | <0.001 |  | 1.23 (1.03, 1.46) | <0.05 |  | 1.40 (1.08, 1.81) | <0.05 |  | 1.21 (1.01, 1.45) | <0.05 |
|  | Frail | 2.21 (1.82, 2.68) | <0.001 |  | 1.66 (1.37, 2.01) | <0.001 |  | 3.41 (2.98, 3.89) | <0.001 |  | 1.29 (1.10, 1.52) | <0.05 |  | 2.90 (2.28, 3.68) | <0.001 |  | 1.96 (1.65, 2.33) | <0.001 |

**Notes.** Stabilized inverse probability weights were estimated from cohort-specific models predicting inclusion in the final analytic sample, using age, sex, education, marital status, smoking status, drinking status, body mass index, baseline frailty status, and baseline cognitive z-score. Robust participants and the high-score trajectory served as reference groups. Models were adjusted for age, sex, education, marital status, smoking status, drinking status, and body mass index.

**Abbreviations:** CHARLS, China Health and Retirement Longitudinal Study; CI, confidence interval; ELSA, English Longitudinal Study of Ageing; HRS, Health and Retirement Study; IPW, inverse probability weighting; OR, odds ratio.

**Table S27.** Association between two-wave frailty change and cognitive trajectories using inverse probability weighting

| **Two-wave frailty change** | | **CHARLS (n = 5876)** | | | | |  | **ELSA (n = 7134)** | | | | |  | **HRS (n = 6576)** | | | | |
| --- | --- | --- | --- | --- | --- | --- | --- | --- | --- | --- | --- | --- | --- | --- | --- | --- | --- | --- |
|  |  | **Low-score trajectory (vs high-score trajectory)** | |  | **Moderate-score trajectory (vs high-score trajectory)** | |  | **Low-score trajectory (vs high-score trajectory)** | |  | **Moderate-score trajectory (vs high-score trajectory)** | |  | **Low-score trajectory (vs high-score trajectory)** | |  | **Moderate-score trajectory (vs high-score trajectory)** | |
|  |  | **OR (95% CI)**^*^ | ***p*** |  | **OR (95% CI)**^*^ | ***p*** |  | **OR (95% CI)**^*^ | ***p*** |  | **OR (95% CI)**^*^ | ***p*** |  | **OR (95% CI)**^*^ | ***p*** |  | **OR (95% CI)**^*^ | ***p*** |
| **Global cognitive trajectories** | |  |  |  |  |  |  |  |  |  |  |  |  |  |  |  |  |  |
|  | Stable robust | 1 (reference) |  |  | 1 (reference) |  |  | 1 (reference) |  |  | 1 (reference) |  |  | 1 (reference) |  |  | 1 (reference) |  |
|  | Robust to pre-frail/frail | 1.72 (1.35, 2.18) | <0.001 |  | 1.37 (1.12, 1.67) | <0.05 |  | 1.14 (0.79, 1.63) | 0.494 |  | 1.31 (1.06, 1.60) | <0.05 |  | 1.52 (1.03, 2.25) | <0.05 |  | 1.40 (1.07, 1.83) | <0.05 |
|  | Stable pre-frail | 1 (reference) |  |  | 1 (reference) |  |  | 1 (reference) |  |  | 1 (reference) |  |  | 1 (reference) |  |  | 1 (reference) |  |
|  | Pre-frail to robust | 0.79 (0.59, 1.06) | 0.111 |  | 0.84 (0.66, 1.08) | 0.179 |  | 0.86 (0.58, 1.29) | 0.479 |  | 0.99 (0.77, 1.28) | 0.957 |  | 0.66 (0.39, 1.13) | 0.130 |  | 0.91 (0.64, 1.30) | 0.605 |
|  | Pre-frail to frail | 1.96 (1.45, 2.63) | <0.001 |  | 1.52 (1.15, 1.99) | <0.05 |  | 2.55 (1.81, 3.58) | <0.001 |  | 1.55 (1.18, 2.05) | <0.05 |  | 2.06 (1.52, 2.79) | <0.001 |  | 1.59 (1.25, 2.03) | <0.001 |
|  | Stable frail | 1 (reference) |  |  | 1 (reference) |  |  | 1 (reference) |  |  | 1 (reference) |  |  | 1 (reference) |  |  | 1 (reference) |  |
|  | Frail to robust/pre-frail | 0.86 (0.57, 1.31) | 0.489 |  | 0.79 (0.53, 1.19) | 0.268 |  | 0.47 (0.30, 0.73) | <0.001 |  | 0.58 (0.40, 0.82) | <0.05 |  | 0.44 (0.28, 0.69) | <0.001 |  | 0.65 (0.44, 0.95) | <0.05 |
| **Memory trajectories** | |  |  |  |  |  |  |  |  |  |  |  |  |  |  |  |  |  |
|  | Stable robust | 1 (reference) |  |  | 1 (reference) |  |  | 1 (reference) |  |  | 1 (reference) |  |  | 1 (reference) |  |  | 1 (reference) |  |
|  | Robust to pre-frail/frail | 1.66 (1.25, 2.21) | <0.001 |  | 1.33 (1.02, 1.72) | <0.05 |  | 1.39 (1.00, 1.94) | <0.05 |  | 1.26 (0.98, 1.60) | 0.069 |  | 1.51 (1.00, 2.29) | 0.051 |  | 1.42 (1.04, 1.94) | <0.05 |
|  | Stable pre-frail | 1 (reference) |  |  | 1 (reference) |  |  | 1 (reference) |  |  | 1 (reference) |  |  | 1 (reference) |  |  | 1 (reference) |  |
|  | Pre-frail to robust | 0.55 (0.38, 0.79) | <0.05 |  | 0.56 (0.41, 0.78) | <0.001 |  | 0.98 (0.66, 1.45) | 0.959 |  | 0.97 (0.71, 1.31) | 0.820 |  | 0.52 (0.31, 0.89) | <0.05 |  | 0.55 (0.37, 0.82) | <0.05 |
|  | Pre-frail to frail | 2.02 (1.27, 3.21) | <0.05 |  | 1.45 (0.93, 2.28) | 0.104 |  | 2.12 (1.41, 3.18) | <0.001 |  | 1.20 (0.83, 1.73) | 0.338 |  | 1.64 (1.15, 2.33) | <0.05 |  | 1.22 (0.90, 1.66) | 0.199 |
|  | Stable frail | 1 (reference) |  |  | 1 (reference) |  |  | 1 (reference) |  |  | 1 (reference) |  |  | 1 (reference) |  |  | 1 (reference) |  |
|  | Frail to robust/pre-frail | 0.72 (0.38, 1.38) | 0.321 |  | 0.67 (0.36, 1.28) | 0.225 |  | 0.45 (0.26, 0.78) | <0.05 |  | 0.55 (0.33, 0.89) | <0.05 |  | 0.56 (0.32, 0.97) | <0.05 |  | 0.66 (0.40, 1.09) | 0.108 |
| **Orientation trajectories** | |  |  |  |  |  |  |  |  |  |  |  |  |  |  |  |  |  |
|  | Stable robust | 1 (reference) |  |  |  |  |  | 1 (reference) |  |  |  |  |  | 1 (reference) |  |  |  |  |
|  | Robust to pre-frail/frail | 1.27 (1.04, 1.56) | <0.05 |  |  |  |  | 1.21 (0.82, 1.74) | 0.203 |  |  |  |  | 1.16 (0.70, 1.91) | 0.563 |  |  |  |
|  | Stable pre-frail | 1 (reference) |  |  |  |  |  | 1 (reference) |  |  |  |  |  | 1 (reference) |  |  |  |  |
|  | Pre-frail to robust | 0.90 (0.70, 1.16) | 0.404 |  |  |  |  | 0.57 (0.32, 1.04) | 0.065 |  |  |  |  | 0.66 (0.30, 1.45) | 0.303 |  |  |  |
|  | Pre-frail to frail | 1.84 (1.46, 2.32) | <0.001 |  |  |  |  | 1.48 (1.01, 2.16) | <0.05 |  |  |  |  | 1.15 (0.79, 1.68) | 0.456 |  |  |  |
|  | Stable frail | 1 (reference) |  |  |  |  |  | 1 (reference) |  |  |  |  |  | 1 (reference) |  |  |  |  |
|  | Frail to robust/pre-frail | 0.87 (0.63, 1.20) | 0.394 |  |  |  |  | 0.51 (0.31, 0.85) | <0.05 |  |  |  |  | 0.63 (0.39, 1.02) | 0.061 |  |  |  |
| **Executive function trajectories** | |  |  |  |  |  |  |  |  |  |  |  |  |  |  |  |  |  |
|  | Stable robust | 1 (reference) |  |  | 1 (reference) |  |  | 1 (reference) |  |  | 1 (reference) |  |  | 1 (reference) |  |  | 1 (reference) |  |
|  | Robust to pre-frail/frail | 1.59 (1.28, 1.98) | <0.001 |  | 1.19 (0.97, 1.47) | 0.093 |  | 1.23 (0.85, 1.79) | 0.279 |  | 1.37 (1.05, 1.80) | <0.05 |  | 1.69 (1.16, 2.47) | <0.05 |  | 1.10 (0.82, 1.49) | 0.525 |
|  | Stable pre-frail | 1 (reference) |  |  | 1 (reference) |  |  | 1 (reference) |  |  | 1 (reference) |  |  | 1 (reference) |  |  | 1 (reference) |  |
|  | Pre-frail to robust | 0.81 (0.62, 1.07) | 0.139 |  | 0.95 (0.74, 1.23) | 0.714 |  | 0.87 (0.56, 1.33) | 0.515 |  | 0.97 (0.71, 1.31) | 0.826 |  | 0.41 (0.21, 0.80) | <0.05 |  | 0.97 (0.65, 1.42) | 0.699 |
|  | Pre-frail to frail | 1.41 (1.08, 1.84) | <0.05 |  | 1.32 (1.01, 1.71) | <0.05 |  | 1.77 (1.29, 2.44) | <0.001 |  | 1.14 (0.83, 1.58) | 0.414 |  | 1.30 (0.96, 1.75) | 0.087 |  | 1.35 (1.09, 1.67) | <0.05 |
|  | Stable frail | 1 (reference) |  |  | 1 (reference) |  |  | 1 (reference) |  |  | 1 (reference) |  |  | 1 (reference) |  |  | 1 (reference) |  |
|  | Frail to robust/pre-frail | 0.67 (0.46, 0.98) | <0.05 |  | 0.68 (0.46, 1.01) | 0.055 |  | 0.49 (0.31, 0.75) | <0.05 |  | 0.84 (0.57, 1.23) | 0.363 |  | 0.51 (0.34, 0.78) | <0.05 |  | 0.56 (0.40, 0.79) | <0.05 |

**Notes.** Stabilized inverse probability weights were estimated from cohort-specific models predicting inclusion in the final analytic sample, using age, sex, education, marital status, smoking status, drinking status, body mass index, baseline frailty status, and baseline cognitive z-score. Two-wave frailty change was defined from the preceding survey to the baseline survey. Stable robust, stable pre-frail, and stable frail participants served as reference groups within each initial frailty-status stratum; the high-score trajectory served as the outcome reference. Models were adjusted for age, sex, education, marital status, smoking status, drinking status, and body mass index.
**Abbreviations:** OR, odds ratio; CI, confidence interval; FI, frailty index; IPW, inverse probability weighting; CHARLS, China Health and Retirement Longitudinal Study; ELSA, English Longitudinal Study of Ageing; HRS, Health and Retirement Study.

**Table S28.** Association between mean frailty burden and cognitive trajectories using inverse probability weighting

| **Mean frailty burden** | | **CHARLS (n = 5876)** | | | | |  | **ELSA (n = 7134)** | | | | |  | **HRS (n = 6576)** | | | | |
| --- | --- | --- | --- | --- | --- | --- | --- | --- | --- | --- | --- | --- | --- | --- | --- | --- | --- | --- |
|  |  | **Low-score trajectory (vs high-score trajectory)** | |  | **Moderate-score trajectory (vs high-score trajectory)** | |  | **Low-score trajectory (vs high-score trajectory)** | |  | **Moderate-score trajectory (vs high-score trajectory)** | |  | **Low-score trajectory (vs high-score trajectory)** | |  | **Moderate-score trajectory (vs high-score trajectory)** | |
|  |  | **OR (95% CI)**^*^ | ***p*** |  | **OR (95% CI)**^*^ | ***p*** |  | **OR (95% CI)**^*^ | ***p*** |  | **OR (95% CI)**^*^ | ***p*** |  | **OR (95% CI)**^*^ | ***p*** |  | **OR (95% CI)**^*^ | ***p*** |
| **Global cognitive trajectories** | |  |  |  |  |  |  |  |  |  |  |  |  |  |  |  |  |  |
|  | T1 | 1 (reference) |  |  | 1 (reference) |  |  | 1 (reference) |  |  | 1 (reference) |  |  | 1 (reference) |  |  | 1 (reference) |  |
|  | T2 | 1.25 (1.03, 1.53) | <0.05 |  | 1.22 (1.04, 1.43) | <0.05 |  | 1.12 (0.92, 1.37) | 0.250 |  | 1.13 (0.99, 1.30) | 0.063 |  | 1.96 (1.56, 2.46) | <0.001 |  | 1.67 (1.42, 1.96) | <0.001 |
|  | T3 | 2.46 (2.03, 2.99) | <0.001 |  | 1.68 (1.43, 1.98) | <0.001 |  | 4.30 (3.55, 5.20) | <0.001 |  | 2.16 (1.87, 2.51) | <0.001 |  | 5.92 (4.74, 7.39) | <0.001 |  | 2.59 (2.18, 3.07) | <0.001 |
|  | *P for trend* |  | <0.001 |  |  | <0.001 |  |  | <0.001 |  |  | <0.001 |  |  | <0.001 |  |  | <0.001 |
| **Memory trajectories** | |  |  |  |  |  |  |  |  |  |  |  |  |  |  |  |  |  |
|  | T1 | 1 (reference) |  |  | 1 (reference) |  |  | 1 (reference) |  |  | 1 (reference) |  |  | 1 (reference) |  |  | 1 (reference) |  |
|  | T2 | 1.29 (1.03, 1.62) | <0.05 |  | 1.20 (0.98, 1.47) | 0.076 |  | 1.22 (1.01, 1.50) | <0.05 |  | 1.15 (0.98, 1.36) | 0.095 |  | 2.31 (1.81, 2.95) | <0.001 |  | 1.76 (1.45, 2.13) | <0.001 |
|  | T3 | 2.74 (2.14, 3.51) | <0.001 |  | 1.83 (1.45, 2.31) | <0.001 |  | 4.25 (3.39, 5.33) | <0.001 |  | 2.05 (1.67, 2.51) | <0.001 |  | 6.10 (4.73, 7.87) | <0.001 |  | 2.73 (2.20, 3.38) | <0.001 |
|  | *P for trend* |  | <0.001 |  |  | <0.001 |  |  | <0.001 |  |  | <0.001 |  |  | <0.001 |  |  | <0.001 |
| **Orientation trajectories** | |  |  |  |  |  |  |  |  |  |  |  |  |  |  |  |  |  |
|  | T1 | 1 (reference) |  |  |  |  |  | 1 (reference) |  |  |  |  |  | 1 (reference) |  |  |  |  |
|  | T2 | 1.04 (0.88, 1.23) | 0.661 |  |  |  |  | 1.01 (0.72, 1.40) | 0.959 |  |  |  |  | 1.21 (0.89, 1.64) | 0.225 |  |  |  |
|  | T3 | 1.63 (1.39, 1.91) | <0.001 |  |  |  |  | 2.32 (1.84, 2.93) | <0.001 |  |  |  |  | 2.61 (1.98, 3.42) | <0.001 |  |  |  |
|  | *P for trend* |  | <0.001 |  |  |  |  |  | <0.001 |  |  |  |  |  | <0.001 |  |  |  |
| **Executive function trajectories** | |  |  |  |  |  |  |  |  |  |  |  |  |  |  |  |  |  |
|  | T1 | 1 (reference) |  |  | 1 (reference) |  |  | 1 (reference) |  |  | 1 (reference) |  |  | 1 (reference) |  |  | 1 (reference) |  |
|  | T2 | 1.21 (1.00, 1.46) | <0.05 |  | 1.08 (0.91, 1.28) | 0.367 |  | 1.08 (0.89, 1.31) | 0.443 |  | 1.08 (0.91, 1.28) | 0.399 |  | 1.34 (1.06, 1.69) | <0.05 |  | 1.38 (1.17, 1.62) | <0.001 |
|  | T3 | 1.82 (1.52, 2.17) | <0.001 |  | 1.42 (1.20, 1.68) | <0.001 |  | 2.30 (1.95, 2.73) | <0.001 |  | 1.29 (1.10, 1.53) | <0.05 |  | 2.93 (2.37, 3.64) | <0.001 |  | 2.05 (1.74, 2.41) | <0.001 |
|  | *P for trend* |  | <0.001 |  |  | <0.001 |  |  | <0.001 |  |  | <0.05 |  |  | <0.001 |  |  | <0.001 |

**Notes.** Stabilized inverse probability weights were estimated from cohort-specific models predicting inclusion in the final analytic sample, using age, sex, education, marital status, smoking status, drinking status, body mass index, baseline frailty status, and baseline cognitive z-score. Mean frailty burden was calculated as the mean FI across the preceding and baseline surveys. T1, T2, and T3 indicate the lower, middle, and upper tertiles, respectively. The high-score trajectory served as the reference category. Models were adjusted for age, sex, education, marital status, smoking status, drinking status, and body mass index.
**Abbreviations:** OR, odds ratio; CI, confidence interval; FI, frailty index; IPW, inverse probability weighting; CHARLS, China Health and Retirement Longitudinal Study; ELSA, English Longitudinal Study of Ageing; HRS, Health and Retirement Study.

**Table S29.** Association between baseline frailty status and cognitive trajectories after excluding depression/mental health-related deficits from the frailty index

| **Frailty status** | | **CHARLS (n = 5876)** | | | | |  | **ELSA (n = 7134)** | | | | |  | **HRS (n = 6576)** | | | | |
| --- | --- | --- | --- | --- | --- | --- | --- | --- | --- | --- | --- | --- | --- | --- | --- | --- | --- | --- |
|  |  | **Low-score trajectory (vs high-score trajectory)** | |  | **Moderate-score trajectory (vs high-score trajectory)** | |  | **Low-score trajectory (vs high-score trajectory)** | |  | **Moderate-score trajectory (vs high-score trajectory)** | |  | **Low-score trajectory (vs high-score trajectory)** | |  | **Moderate-score trajectory (vs high-score trajectory)** | |
|  |  | **OR (95% CI)^*^** | ***p*** |  | **OR (95% CI)^*^** | ***p*** |  | **OR (95% CI)^*^** | ***p*** |  | **OR (95% CI)^*^** | ***p*** |  | **OR (95% CI)^*^** | ***p*** |  | **OR (95% CI)^*^** | ***p*** |
| Global cognitive trajectories | |  |  |  |  |  |  |  |  |  |  |  |  |  |  |  |  |  |
|  | Robust | **1 (reference)** |  |  | **1 (reference)** |  |  | **1 (reference)** |  |  | **1 (reference)** |  |  | **1 (reference)** |  |  | **1 (reference)** |  |
|  | Pre-frail | 1.56 (1.31, 1.85) | <0.001 |  | 1.28 (1.12, 1.46) | <0.001 |  | 1.77 (1.40, 2.26) | <0.001 |  | 1.28 (1.13, 1.45) | <0.001 |  | 1.74 (1.35, 2.25) | <0.001 |  | 1.48 (1.29, 1.71) | <0.001 |
|  | Frail | 2.88 (2.23, 3.72) | <0.001 |  | 2.03 (1.62, 2.53) | <0.001 |  | 3.18 (2.40, 4.20) | <0.001 |  | 1.93 (1.63, 2.30) | <0.001 |  | 4.05 (3.10, 5.28) | <0.001 |  | 2.18 (1.85, 2.58) | <0.001 |
| Memory trajectories | |  |  |  |  |  |  |  |  |  |  |  |  |  |  |  |  |  |
|  | Robust | 1 (reference) |  |  | 1 (reference) |  |  | 1 (reference) |  |  | 1 (reference) |  |  | 1 (reference) |  |  | 1 (reference) |  |
|  | Pre-frail | 1.75 (1.44, 2.12) | <0.001 |  | 1.43 (1.20, 1.69) | <0.001 |  | 1.75 (1.42, 2.15) | <0.001 |  | 1.21 (1.05, 1.40) | <0.05 |  | 2.06 (1.63, 2.61) | <0.001 |  | 1.63 (1.39, 1.92) | <0.001 |
|  | Frail | 3.49 (2.42, 5.02) | <0.001 |  | 2.16 (1.52, 3.07) | <0.001 |  | 3.37 (2.54, 4.47) | <0.001 |  | 1.87 (1.49, 2.35) | <0.001 |  | 3.90 (2.99, 5.08) | <0.001 |  | 2.11 (1.73, 2.58) | <0.001 |
| Orientation trajectories | |  |  |  |  |  |  |  |  |  |  |  |  |  |  |  |  |  |
|  | Robust | 1 (reference) |  |  |  |  |  | 1 (reference) |  |  |  |  |  | 1 (reference) |  |  |  |  |
|  | Pre-frail | 1.24 (1.08, 1.44) | <0.05 |  |  |  |  | 1.42 (1.02, 1.98) | <0.05 |  |  |  |  | 1.26 (0.89, 1.82) | 0.201 |  |  |  |
|  | Frail | 1.90 (1.55, 2.32) | <0.001 |  |  |  |  | 2.10 (1.46, 3.03) | <0.001 |  |  |  |  | 2.07 (1.45, 3.00) | <0.001 |  |  |  |
| Executive function trajectories | |  |  |  |  |  |  |  |  |  |  |  |  |  |  |  |  |  |
|  | Robust | 1 (reference) |  |  | 1 (reference) |  |  | 1 (reference) |  |  | 1 (reference) |  |  | 1 (reference) |  |  | 1 (reference) |  |
|  | Pre-frail | 1.43 (1.22, 1.68) | <0.001 |  | 1.13 (0.98, 1.30) | 0.087 |  | 1.44 (1.11, 1.87) | <0.05 |  | 1.30 (1.09, 1.55) | <0.05 |  | 1.43 (1.07, 1.92) | <0.05 |  | 1.34 (1.14, 1.58) | <0.001 |
|  | Frail | 2.14 (1.70, 2.71) | <0.001 |  | 1.63 (1.31, 2.02) | <0.001 |  | 2.27 (1.70, 3.02) | <0.001 |  | 1.38 (1.11, 1.71) | <0.05 |  | 2.61 (1.95, 3.50) | <0.001 |  | 1.66 (1.39, 1.98) | <0.001 |

**Notes.** The modified FI was recalculated after excluding depression/mental health-related deficits. Frailty status was defined using the same cut-off points as in the primary analysis: robust, FI ≤ 0.10; pre-frail, 0.10 < FI < 0.25; and frail, FI ≥ 0.25. Robust participants served as the reference group. For each cognitive outcome, the high-score trajectory served as the reference category. Models were adjusted for age, sex, education, marital status, smoking status, drinking status, and body mass index.

**Abbreviations:** BMI, body mass index; CI, confidence interval; CHARLS, China Health and Retirement Longitudinal Study; ELSA, English Longitudinal Study of Ageing; FI, frailty index; HRS, Health and Retirement Study; OR, odds ratio.

**Table S30.** Association between two-wave frailty change and cognitive trajectories after excluding depression/mental health-related deficits from the frailty index

| **Two-wave frailty change** | | **CHARLS (n = 5876)** | | | | |  | **ELSA (n = 7134)** | | | | |  | **HRS (n = 6576)** | | | | |
| --- | --- | --- | --- | --- | --- | --- | --- | --- | --- | --- | --- | --- | --- | --- | --- | --- | --- | --- |
|  |  | **Low-score trajectory (vs high-score trajectory)** | |  | **Moderate-score trajectory (vs high-score trajectory)** | |  | **Low-score trajectory (vs high-score trajectory)** | |  | **Moderate-score trajectory (vs high-score trajectory)** | |  | **Low-score trajectory (vs high-score trajectory)** | |  | **Moderate-score trajectory (vs high-score trajectory)** | |
|  |  | **OR (95% CI)**^*^ | ***p*** |  | **OR (95% CI)**^*^ | ***p*** |  | **OR (95% CI)**^*^ | ***p*** |  | **OR (95% CI)**^*^ | ***p*** |  | **OR (95% CI)**^*^ | ***p*** |  | **OR (95% CI)**^*^ | ***p*** |
| **Global cognitive trajectories** | |  |  |  |  |  |  |  |  |  |  |  |  |  |  |  |  |  |
|  | Stable robust | **1 (reference)** |  |  | **1 (reference)** |  |  | **1 (reference)** |  |  | **1 (reference)** |  |  | **1 (reference)** |  |  | **1 (reference)** |  |
|  | Robust to pre-frail/frail | 1.75 (1.38, 2.22) | <0.001 |  | 1.42 (1.19, 1.71) | <0.001 |  | 1.27 (0.86, 1.87) | 0.235 |  | 1.32 (1.09, 1.59) | 0.005 |  | 1.74 (1.15, 2.61) | 0.008 |  | 1.56 (1.24, 1.96) | <0.001 |
|  | Stable pre-frail | 1 (reference) |  |  | 1 (reference) |  |  | 1 (reference) |  |  | 1 (reference) |  |  | 1 (reference) |  |  | 1 (reference) |  |
|  | Pre-frail to robust | 0.85 (0.62, 1.15) | 0.291 |  | 0.91 (0.71, 1.17) | 0.473 |  | 0.70 (0.44, 1.12) | 0.137 |  | 0.95 (0.74, 1.21) | 0.653 |  | 0.93 (0.56, 1.54) | 0.787 |  | 0.96 (0.71, 1.30) | 0.792 |
|  | Pre-frail to frail | 1.62 (1.14, 2.31) | 0.007 |  | 1.59 (1.17, 2.15) | 0.003 |  | 1.54 (1.02, 2.34) | 0.041 |  | 1.30 (0.97, 1.74) | 0.076 |  | 1.37 (0.99, 1.90) | 0.060 |  | 1.17 (0.94, 1.46) | 0.169 |
|  | Stable frail | 1 (reference) |  |  | 1 (reference) |  |  | 1 (reference) |  |  | 1 (reference) |  |  | 1 (reference) |  |  | 1 (reference) |  |
|  | Frail to robust/pre-frail | 1.00 (0.60, 1.66) | 0.976 |  | 0.95 (0.59, 1.51) | 0.822 |  | 1.00 (0.63, 1.58) | 0.995 |  | 0.66 (0.47, 0.94) | 0.019 |  | 0.69 (0.45, 1.06) | 0.090 |  | 0.85 (0.61, 1.17) | 0.316 |
| **Memory trajectories** | |  |  |  |  |  |  |  |  |  |  |  |  |  |  |  |  |  |
|  | Stable robust | **1 (reference)** |  |  | **1 (reference)** |  |  | **1 (reference)** |  |  | **1 (reference)** |  |  | **1 (reference)** |  |  | **1 (reference)** |  |
|  | Robust to pre-frail/frail | 1.78 (1.36, 2.32) | <0.001 |  | 1.39 (1.09, 1.76) | 0.007 |  | 1.63 (1.19, 2.24) | 0.003 |  | 1.21 (0.97, 1.51) | 0.088 |  | 1.48 (1.00, 2.19) | 0.049 |  | 1.54 (1.19, 1.99) | <0.001 |
|  | Stable pre-frail | 1 (reference) |  |  | 1 (reference) |  |  | 1 (reference) |  |  | 1 (reference) |  |  | 1 (reference) |  |  | 1 (reference) |  |
|  | Pre-frail to robust | 0.64 (0.45, 0.91) | 0.014 |  | 0.67 (0.49, 0.93) | 0.016 |  | 0.99 (0.67, 1.47) | 0.954 |  | 0.97 (0.71, 1.31) | 0.831 |  | 0.63 (0.39, 1.02) | 0.059 |  | 0.67 (0.47, 0.93) | 0.018 |
|  | Pre-frail to frail | 1.78 (1.07, 2.99) | 0.028 |  | 1.49 (0.91, 2.44) | 0.113 |  | 1.56 (1.00, 2.44) | 0.052 |  | 1.16 (0.79, 1.69) | 0.448 |  | 1.10 (0.78, 1.55) | 0.572 |  | 1.00 (0.76, 1.32) | 0.993 |
|  | Stable frail | 1 (reference) |  |  | 1 (reference) |  |  | 1 (reference) |  |  | 1 (reference) |  |  | 1 (reference) |  |  | 1 (reference) |  |
|  | Frail to robust/pre-frail | 0.73 (0.37, 1.45) | 0.368 |  | 0.66 (0.34, 1.29) | 0.222 |  | 0.75 (0.44, 1.27) | 0.280 |  | 0.59 (0.37, 0.92) | 0.021 |  | 0.77 (0.48, 1.25) | 0.297 |  | 0.84 (0.55, 1.28) | 0.413 |
| **Orientation trajectories** | |  |  |  |  |  |  |  |  |  |  |  |  |  |  |  |  |  |
|  | Stable robust | **1 (reference)** |  |  |  |  |  | **1 (reference)** |  |  |  |  |  | **1 (reference)** |  |  |  |  |
|  | Robust to pre-frail/frail | 1.24 (1.02, 1.51) | 0.034 |  |  |  |  | 1.10 (0.64, 1.84) | 0.711 |  |  |  |  | 1.13 (0.62, 2.00) | 0.675 |  |  |  |
|  | Stable pre-frail | 1 (reference) |  |  |  |  |  | 1 (reference) |  |  |  |  |  | 1 (reference) |  |  |  |  |
|  | Pre-frail to robust | 0.78 (0.59, 1.02) | 0.070 |  |  |  |  | 0.56 (0.25, 1.09) | 0.112 |  |  |  |  | 0.82 (0.36, 1.64) | 0.602 |  |  |  |
|  | Pre-frail to frail | 1.32 (1.00, 1.74) | 0.051 |  |  |  |  | 1.01 (0.56, 1.74) | 0.964 |  |  |  |  | 1.51 (0.99, 2.25) | 0.058 |  |  |  |
|  | Stable frail | 1 (reference) |  |  |  |  |  | 1 (reference) |  |  |  |  |  | 1 (reference) |  |  |  |  |
|  | Frail to robust/pre-frail | 0.90 (0.60, 1.33) | 0.585 |  |  |  |  | 0.85 (0.46, 1.46) | 0.565 |  |  |  |  | 0.98 (0.58, 1.57) | 0.922 |  |  |  |
| **Executive function trajectories** | |  |  |  |  |  |  |  |  |  |  |  |  |  |  |  |  |  |
|  | Stable robust | **1 (reference)** |  |  | **1 (reference)** |  |  | **1 (reference)** |  |  | **1 (reference)** |  |  | **1 (reference)** |  |  | **1 (reference)** |  |
|  | Robust to pre-frail/frail | 1.75 (1.40, 2.19) | <0.001 |  | 1.17 (0.97, 1.42) | 0.108 |  | 1.13 (0.73, 1.74) | 0.589 |  | 1.54 (1.19, 1.99) | <0.001 |  | 1.80 (1.16, 2.79) | 0.009 |  | 1.50 (1.16, 1.94) | 0.002 |
|  | Stable pre-frail | 1 (reference) |  |  | 1 (reference) |  |  | 1 (reference) |  |  | 1 (reference) |  |  | 1 (reference) |  |  | 1 (reference) |  |
|  | Pre-frail to robust | 0.94 (0.70, 1.26) | 0.679 |  | 0.93 (0.72, 1.21) | 0.591 |  | 0.87 (0.54, 1.41) | 0.574 |  | 0.99 (0.68, 1.42) | 0.621 |  | 0.80 (0.44, 1.46) | 0.470 |  | 0.86 (0.61, 1.19) | 0.359 |
|  | Pre-frail to frail | 1.31 (0.95, 1.81) | 0.100 |  | 1.25 (0.93, 1.69) | 0.133 |  | 1.41 (0.94, 2.12) | 0.098 |  | 1.04 (0.74, 1.48) | 0.806 |  | 1.44 (1.02, 2.05) | 0.041 |  | 1.08 (0.86, 1.34) | 0.515 |
|  | Stable frail | 1 (reference) |  |  | 1 (reference) |  |  | 1 (reference) |  |  | 1 (reference) |  |  | 1 (reference) |  |  | 1 (reference) |  |
|  | Frail to robust/pre-frail | 0.79 (0.50, 1.25) | 0.315 |  | 0.56 (0.35, 0.89) | 0.015 |  | 0.70 (0.42, 1.17) | 0.176 |  | 0.93 (0.63, 1.38) | 0.721 |  | 0.76 (0.50, 1.17) | 0.212 |  | 0.77 (0.57, 1.05) | 0.102 |

**Notes.** The modified FI was recalculated after excluding depression/mental health-related deficits. Two-wave frailty change was defined from the preceding survey to the baseline survey. Stable robust, stable pre-frail, and stable frail participants served as the corresponding reference groups within each preceding frailty-status stratum. For each cognitive outcome, the high-score trajectory served as the reference category. Models were adjusted for age, sex, education, marital status, smoking status, drinking status, and body mass index.

**Abbreviations:** BMI, body mass index; CI, confidence interval; CHARLS, China Health and Retirement Longitudinal Study; ELSA, English Longitudinal Study of Ageing; FI, frailty index; HRS, Health and Retirement Study; OR, odds ratio.

**Table S31.** Association between the mean frailty burden and cognitive trajectories after excluding depression/mental health-related deficits from the frailty index

| **Mean frailty burden** | | **CHARLS (n = 5876)** | | | | |  | **ELSA (n = 7134)** | | | | |  | **HRS (n = 6576)** | | | | |
| --- | --- | --- | --- | --- | --- | --- | --- | --- | --- | --- | --- | --- | --- | --- | --- | --- | --- | --- |
|  |  | **Low-score trajectory (vs high-score trajectory)** | |  | **Moderate-score trajectory (vs high-score trajectory)** | |  | **Low-score trajectory (vs high-score trajectory)** | |  | **Moderate-score trajectory (vs high-score trajectory)** | |  | **Low-score trajectory (vs high-score trajectory)** | |  | **Moderate-score trajectory (vs high-score trajectory)** | |
|  |  | **OR (95% CI)**^*^ | ***p*** |  | **OR (95% CI)**^*^ | ***p*** |  | **OR (95% CI)**^*^ | ***p*** |  | **OR (95% CI)**^*^ | ***p*** |  | **OR (95% CI)**^*^ | ***p*** |  | **OR (95% CI)**^*^ | ***p*** |
| **Global cognitive trajectories** | |  |  |  |  |  |  |  |  |  |  |  |  |  |  |  |  |  |
|  | T1 | 1 (reference) |  |  | 1 (reference) |  |  | 1 (reference) |  |  | 1 (reference) |  |  | 1 (reference) |  |  | 1 (reference) |  |
|  | T2 | 1.24 (1.01, 1.51) | <0.05 |  | 1.22 (1.05, 1.41) | <0.05 |  | 1.20 (0.91, 1.59) | 0.200 |  | 1.14 (1.00, 1.30) | 0.059 |  | 1.54 (1.22, 1.96) | <0.001 |  | 1.43 (1.25, 1.64) | <0.001 |
|  | T3 | 2.44 (2.00, 2.98) | <0.001 |  | 1.75 (1.49, 2.05) | <0.001 |  | 2.61 (2.00, 3.41) | <0.001 |  | 1.60 (1.38, 1.85) | <0.001 |  | 3.87 (3.04, 4.91) | <0.001 |  | 2.10 (1.79, 2.46) | <0.001 |
|  | *P for trend* |  | <0.001 |  |  | <0.001 |  |  | <0.001 |  |  | <0.001 |  |  | <0.001 |  |  | <0.001 |
| **Memory trajectories** | |  |  |  |  |  |  |  |  |  |  |  |  |  |  |  |  |  |
|  | T1 | 1 (reference) |  |  | 1 (reference) |  |  | 1 (reference) |  |  | 1 (reference) |  |  | 1 (reference) |  |  | 1 (reference) |  |
|  | T2 | 1.33 (1.08, 1.64) | <0.05 |  | 1.21 (1.02, 1.45) | <0.05 |  | 1.49 (1.18, 1.87) | <0.001 |  | 1.27 (1.10, 1.47) | <0.05 |  | 1.80 (1.43, 2.25) | <0.001 |  | 1.55 (1.32, 1.82) | <0.001 |
|  | T3 | 2.72 (2.14, 3.45) | <0.001 |  | 1.77 (1.42, 2.20) | <0.001 |  | 2.94 (2.31, 3.74) | <0.001 |  | 1.62 (1.36, 1.92) | <0.001 |  | 3.95 (3.08, 5.07) | <0.001 |  | 2.14 (1.77, 2.60) | <0.001 |
|  | *P for trend* |  | <0.001 |  |  | <0.001 |  |  | <0.001 |  |  | <0.001 |  |  | <0.001 |  |  | <0.001 |
| **Orientation trajectories** | |  |  |  |  |  |  |  |  |  |  |  |  |  |  |  |  |  |
|  | T1 | 1 (reference) |  |  |  |  |  | 1 (reference) |  |  |  |  |  | 1 (reference) |  |  |  |  |
|  | T2 | 1.04 (0.88, 1.23) | 0.655 |  |  |  |  | 1.08 (0.72, 1.61) | 0.710 |  |  |  |  | 1.15 (0.82, 1.62) | 0.409 |  |  |  |
|  | T3 | 1.67 (1.42, 1.97) | <0.001 |  |  |  |  | 2.12 (1.48, 3.08) | <0.001 |  |  |  |  | 2.22 (1.61, 3.10) | <0.001 |  |  |  |
|  | *P for trend* |  | <0.001 |  |  |  |  |  | <0.001 |  |  |  |  |  | <0.001 |  |  |  |
| **Executive function trajectories** | |  |  |  |  |  |  |  |  |  |  |  |  |  |  |  |  |  |
|  | T1 | 1 (reference) |  |  | 1 (reference) |  |  | 1 (reference) |  |  | 1 (reference) |  |  | 1 (reference) |  |  | 1 (reference) |  |
|  | T2 | 1.26 (1.05, 1.53) | <0.05 |  | 1.02 (0.87, 1.19) | 0.812 |  | 1.32 (0.97, 1.78) | 0.074 |  | 1.22 (1.00, 1.48) | <0.05 |  | 1.25 (0.95, 1.64) | 0.107 |  | 1.30 (1.12, 1.52) | <0.001 |
|  | T3 | 1.86 (1.54, 2.24) | <0.001 |  | 1.42 (1.21, 1.67) | <0.001 |  | 2.02 (1.52, 2.70) | <0.001 |  | 1.38 (1.13, 1.69) | <0.05 |  | 2.39 (1.84, 3.09) | <0.001 |  | 1.55 (1.32, 1.82) | <0.001 |
|  | *P for trend* |  | <0.001 |  |  | <0.001 |  |  | <0.001 |  |  | <0.05 |  |  | <0.001 |  |  | <0.001 |

**Notes.** The modified FI was recalculated after excluding depression/mental health-related deficits. The mean frailty burden was calculated as the mean of the FI measured at the preceding survey and the baseline survey. Participants were categorized into cohort-specific tertiles of the mean frailty burden, with T1 representing the lowest tertile and serving as the reference group. T2 and T3 represented the middle and highest tertiles, respectively. For each cognitive outcome, the high-score trajectory served as the reference category. Models were adjusted for age, sex, education, marital status, smoking status, drinking status, and body mass index. *P* for trend was calculated by treating FI tertiles as an ordinal variable.

**Abbreviations:** BMI, body mass index; CI, confidence interval; CHARLS, China Health and Retirement Longitudinal Study; ELSA, English Longitudinal Study of Ageing; FI, frailty index; HRS, Health and Retirement Study; OR, odds ratio.

**Table S32.** Association between baseline frailty status and cognitive trajectories after excluding disability-related deficits from the frailty index

| **Frailty status** | | **CHARLS (n = 5876)** | | | | |  | **ELSA (n = 7134)** | | | | |  | **HRS (n = 6576)** | | | | |
| --- | --- | --- | --- | --- | --- | --- | --- | --- | --- | --- | --- | --- | --- | --- | --- | --- | --- | --- |
|  |  | **Low-score trajectory (vs high-score trajectory)** | |  | **Moderate-score trajectory (vs high-score trajectory)** | |  | **Low-score trajectory (vs high-score trajectory)** | |  | **Moderate-score trajectory (vs high-score trajectory)** | |  | **Low-score trajectory (vs high-score trajectory)** | |  | **Moderate-score trajectory (vs high-score trajectory)** | |
|  |  | **OR (95% CI)^*^** | ***p*** |  | **OR (95% CI)^*^** | ***p*** |  | **OR (95% CI)^*^** | ***p*** |  | **OR (95% CI)^*^** | ***p*** |  | **OR (95% CI)^*^** | ***p*** |  | **OR (95% CI)^*^** | ***p*** |
| Global cognitive trajectories | |  |  |  |  |  |  |  |  |  |  |  |  |  |  |  |  |  |
|  | Robust | 1 (reference) |  |  | 1 (reference) |  |  | 1 (reference) |  |  | 1 (reference) |  |  | 1 (reference) |  |  | 1 (reference) |  |
|  | Pre-frail | 1.33 (1.09, 1.61) | <0.05 |  | 1.32 (1.15, 1.53) | <0.001 |  | 1.20 (0.91, 1.59) | 0.202 |  | 1.16 (1.02, 1.32) | <0.05 |  | 1.76 (1.23, 2.53) | <0.05 |  | 1.38 (1.15, 1.66) | <0.001 |
|  | Frail | 2.33 (1.87, 2.91) | <0.001 |  | 1.82 (1.52, 2.16) | <0.001 |  | 2.85 (2.14, 3.80) | <0.001 |  | 1.80 (1.55, 2.10) | <0.001 |  | 3.84 (2.71, 5.45) | <0.001 |  | 2.08 (1.73, 2.51) | <0.001 |
| Memory trajectories | |  |  |  |  |  |  |  |  |  |  |  |  |  |  |  |  |  |
|  | Robust | 1 (reference) |  |  | 1 (reference) |  |  | 1 (reference) |  |  | 1 (reference) |  |  | 1 (reference) |  |  | 1 (reference) |  |
|  | Pre-frail | 1.43 (1.17, 1.75) | <0.001 |  | 1.22 (1.02, 1.45) | <0.05 |  | 1.32 (1.05, 1.65) | <0.05 |  | 1.27 (1.10, 1.46) | <0.001 |  | 1.72 (1.25, 2.36) | <0.001 |  | 1.32 (1.09, 1.60) | <0.05 |
|  | Frail | 2.75 (2.11, 3.57) | <0.001 |  | 1.73 (1.36, 2.19) | <0.001 |  | 2.79 (2.17, 3.59) | <0.001 |  | 1.63 (1.36, 1.95) | <0.001 |  | 3.78 (2.76, 5.19) | <0.001 |  | 2.11 (1.72, 2.59) | <0.001 |
| Orientation trajectories | |  |  |  |  |  |  |  |  |  |  |  |  |  |  |  |  |  |
|  | Robust | 1 (reference) |  |  |  |  |  | 1 (reference) |  |  |  |  |  | 1 (reference) |  |  |  |  |
|  | Pre-frail | 1.15 (0.97, 1.35) | 0.100 |  |  |  |  | 1.19 (0.80, 1.79) | 0.385 |  |  |  |  | 1.46 (0.90, 2.50) | 0.146 |  |  |  |
|  | Frail | 1.60 (1.33, 1.92) | <0.001 |  |  |  |  | 2.29 (1.56, 3.43) | <0.001 |  |  |  |  | 1.94 (1.21, 3.28) | <0.05 |  |  |  |
| Executive function trajectories | |  |  |  |  |  |  |  |  |  |  |  |  |  |  |  |  |  |
|  | Robust | 1 (reference) |  |  | 1 (reference) |  |  | 1 (reference) |  |  | 1 (reference) |  |  | 1 (reference) |  |  | 1 (reference) |  |
|  | Pre-frail | 1.25 (1.04, 1.50) | <0.05 |  | 1.13 (0.97, 1.31) | 0.118 |  | 1.13 (0.84, 1.53) | 0.423 |  | 1.07 (0.88, 1.30) | 0.503 |  | 1.63 (1.06, 2.49) | <0.05 |  | 1.17 (0.95, 1.45) | 0.149 |
|  | Frail | 1.72 (1.40, 2.12) | <0.001 |  | 1.39 (1.16, 1.67) | <0.001 |  | 2.12 (1.57, 2.87) | <0.001 |  | 1.36 (1.10, 1.68) | <0.05 |  | 2.76 (1.84, 4.15) | <0.001 |  | 1.53 (1.24, 1.89) | <0.001 |

**Notes.** The modified FI was recalculated after excluding disability-related deficits. Frailty status was defined using the same cut-off points as in the primary analysis: robust, FI ≤ 0.10; pre-frail, 0.10 < FI < 0.25; and frail, FI ≥ 0.25. Robust participants served as the reference group. For each cognitive outcome, the high-score trajectory served as the reference category. Models were adjusted for age, sex, education, marital status, smoking status, drinking status, and body mass index.

**Abbreviations:** BMI, body mass index; CI, confidence interval; CHARLS, China Health and Retirement Longitudinal Study; ELSA, English Longitudinal Study of Ageing; FI, frailty index; HRS, Health and Retirement Study; OR, odds ratio.

**Table S33.** Association between two-wave frailty change and cognitive trajectories after excluding disability-related deficits from the frailty index

| **Two-wave frailty change** | | **CHARLS (n = 5876)** | | | | |  | **ELSA (n = 7134)** | | | | |  | **HRS (n = 6576)** | | | | |
| --- | --- | --- | --- | --- | --- | --- | --- | --- | --- | --- | --- | --- | --- | --- | --- | --- | --- | --- |
|  |  | **Low-score trajectory (vs high-score trajectory)** | |  | **Moderate-score trajectory (vs high-score trajectory)** | |  | **Low-score trajectory (vs high-score trajectory)** | |  | **Moderate-score trajectory (vs high-score trajectory)** | |  | **Low-score trajectory (vs high-score trajectory)** | |  | **Moderate-score trajectory (vs high-score trajectory)** | |
|  |  | **OR (95% CI)**^*^ | ***p*** |  | **OR (95% CI)**^*^ | ***p*** |  | **OR (95% CI)**^*^ | ***p*** |  | **OR (95% CI)**^*^ | ***p*** |  | **OR (95% CI)**^*^ | ***p*** |  | **OR (95% CI)**^*^ | ***p*** |
| **Global cognitive trajectories** | |  |  |  |  |  |  |  |  |  |  |  |  |  |  |  |  |  |
|  | Stable robust | **1 (reference)** |  |  | **1 (reference)** |  |  | **1 (reference)** |  |  | **1 (reference)** |  |  | **1 (reference)** |  |  | **1 (reference)** |  |
|  | Robust to pre-frail/frail | 1.43 (1.06, 1.93) | 0.019 |  | 1.40 (1.13, 1.73) | 0.002 |  | 1.10 (0.69, 1.75) | 0.695 |  | 1.32 (1.07, 1.63) | 0.009 |  | 2.01 (1.14, 3.53) | 0.016 |  | 1.43 (1.06, 1.94) | 0.021 |
|  | Stable pre-frail | 1 (reference) |  |  | 1 (reference) |  |  | 1 (reference) |  |  | 1 (reference) |  |  | 1 (reference) |  |  | 1 (reference) |  |
|  | Pre-frail to robust | 1.09 (0.79, 1.50) | 0.613 |  | 0.87 (0.68, 1.12) | 0.278 |  | 1.11 (0.63, 1.94) | 0.724 |  | 1.05 (0.80, 1.37) | 0.717 |  | 0.88 (0.44, 1.76) | 0.722 |  | 1.31 (0.90, 1.88) | 0.155 |
|  | Pre-frail to frail | 1.67 (1.27, 2.19) | <0.001 |  | 1.25 (1.00, 1.57) | 0.049 |  | 2.23 (1.51, 3.28) | <0.001 |  | 1.51 (1.19, 1.91) | <0.001 |  | 1.56 (1.11, 2.18) | 0.010 |  | 1.33 (1.07, 1.64) | 0.009 |
|  | Stable frail | 1 (reference) |  |  | 1 (reference) |  |  | 1 (reference) |  |  | 1 (reference) |  |  | 1 (reference) |  |  | 1 (reference) |  |
|  | Frail to robust/pre-frail | 0.87 (0.62, 1.23) | 0.433 |  | 0.85 (0.63, 1.15) | 0.299 |  | 0.74 (0.48, 1.14) | 0.173 |  | 0.75 (0.57, 1.00) | 0.049 |  | 0.59 (0.40, 0.89) | 0.013 |  | 0.83 (0.63, 1.09) | 0.170 |
| **Memory trajectories** | |  |  |  |  |  |  |  |  |  |  |  |  |  |  |  |  |  |
|  | Stable robust | **1 (reference)** |  |  | **1 (reference)** |  |  | **1 (reference)** |  |  | **1 (reference)** |  |  | **1 (reference)** |  |  | **1 (reference)** |  |
|  | Robust to pre-frail/frail | 1.54 (1.14, 2.09) | 0.005 |  | 1.31 (1.01, 1.69) | 0.040 |  | 1.17 (0.81, 1.70) | 0.408 |  | 1.26 (1.00, 1.58) | 0.049 |  | 1.73 (1.02, 2.91) | 0.042 |  | 1.43 (1.04, 1.97) | 0.030 |
|  | Stable pre-frail | 1 (reference) |  |  | 1 (reference) |  |  | 1 (reference) |  |  | 1 (reference) |  |  | 1 (reference) |  |  | 1 (reference) |  |
|  | Pre-frail to robust | 1.09 (0.76, 1.57) | 0.631 |  | 1.08 (0.78, 1.50) | 0.627 |  | 1.19 (0.77, 1.83) | 0.429 |  | 0.81 (0.61, 1.08) | 0.153 |  | 0.95 (0.52, 1.75) | 0.868 |  | 1.07 (0.71, 1.59) | 0.758 |
|  | Pre-frail to frail | 1.94 (1.37, 2.74) | <0.001 |  | 1.46 (1.06, 2.01) | 0.021 |  | 1.87 (1.29, 2.70) | <0.001 |  | 1.02 (0.76, 1.37) | 0.877 |  | 1.58 (1.13, 2.21) | 0.007 |  | 1.41 (1.10, 1.82) | 0.007 |
|  | Stable frail | 1 (reference) |  |  | 1 (reference) |  |  | 1 (reference) |  |  | 1 (reference) |  |  | 1 (reference) |  |  | 1 (reference) |  |
|  | Frail to robust/pre-frail | 0.77 (0.49, 1.21) | 0.251 |  | 0.80 (0.52, 1.23) | 0.313 |  | 0.92 (0.60, 1.43) | 0.722 |  | 0.84 (0.59, 1.20) | 0.335 |  | 0.59 (0.39, 0.91) | 0.017 |  | 0.83 (0.59, 1.16) | 0.278 |
| **Orientation trajectories** | |  |  |  |  |  |  |  |  |  |  |  |  |  |  |  |  |  |
|  | Stable robust | **1 (reference)** |  |  |  |  |  | **1 (reference)** |  |  |  |  |  | **1 (reference)** |  |  |  |  |
|  | Robust to pre-frail/frail | 1.26 (0.99, 1.62) | 0.063 |  |  |  |  | 1.01 (0.51, 1.92) | 0.966 |  |  |  |  | 1.98 (0.90, 4.36) | 0.087 |  |  |  |
|  | Stable pre-frail | 1 (reference) |  |  |  |  |  | 1 (reference) |  |  |  |  |  | 1 (reference) |  |  |  |  |
|  | Pre-frail to robust | 1.07 (0.81, 1.40) | 0.619 |  |  |  |  | 0.86 (0.35, 1.80) | 0.707 |  |  |  |  | 1.15 (0.43, 2.59) | 0.750 |  |  |  |
|  | Pre-frail to frail | 1.43 (1.14, 1.79) | 0.002 |  |  |  |  | 1.26 (0.73, 2.10) | 0.388 |  |  |  |  | 1.08 (0.65, 1.74) | 0.767 |  |  |  |
|  | Stable frail | 1 (reference) |  |  |  |  |  | 1 (reference) |  |  |  |  |  | 1 (reference) |  |  |  |  |
|  | Frail to robust/pre-frail | 0.89 (0.67, 1.17) | 0.414 |  |  |  |  | 0.55 (0.28, 1.11) | 0.059 |  |  |  |  | 1.05 (0.64, 1.66) | 0.830 |  |  |  |
| **Executive function trajectories** | |  |  |  |  |  |  |  |  |  |  |  |  |  |  |  |  |  |
|  | Stable robust | **1 (reference)** |  |  | **1 (reference)** |  |  | **1 (reference)** |  |  | **1 (reference)** |  |  | **1 (reference)** |  |  | **1 (reference)** |  |
|  | Robust to pre-frail/frail | 1.32 (1.00, 1.75) | 0.049 |  | 1.23 (0.98, 1.53) | 0.076 |  | 1.01 (0.61, 1.69) | 0.954 |  | 1.06 (0.78, 1.46) | 0.702 |  | 2.03 (1.07, 3.84) | 0.030 |  | 1.12 (0.78, 1.62) | 0.526 |
|  | Stable pre-frail | 1 (reference) |  |  | 1 (reference) |  |  | 1 (reference) |  |  | 1 (reference) |  |  | 1 (reference) |  |  | 1 (reference) |  |
|  | Pre-frail to robust | 0.96 (0.70, 1.30) | 0.787 |  | 0.95 (0.73, 1.24) | 0.722 |  | 1.40 (0.80, 2.44) | 0.241 |  | 1.17 (0.81, 1.71) | 0.400 |  | 0.72 (0.32, 1.60) | 0.422 |  | 1.29 (0.87, 1.90) | 0.208 |
|  | Pre-frail to frail | 1.14 (0.88, 1.48) | 0.321 |  | 1.19 (0.95, 1.50) | 0.135 |  | 2.20 (1.48, 3.28) | <0.001 |  | 1.28 (0.95, 1.74) | 0.106 |  | 1.52 (1.04, 2.22) | 0.029 |  | 1.23 (0.98, 1.53) | 0.077 |
|  | Stable frail | 1 (reference) |  |  | 1 (reference) |  |  | 1 (reference) |  |  | 1 (reference) |  |  | 1 (reference) |  |  | 1 (reference) |  |
|  | Frail to robust/pre-frail | 0.85 (0.62, 1.17) | 0.310 |  | 0.90 (0.66, 1.22) | 0.489 |  | 0.81 (0.52, 1.28) | 0.372 |  | 0.94 (0.66, 1.34) | 0.746 |  | 0.84 (0.55, 1.28) | 0.412 |  | 1.02 (0.78, 1.34) | 0.864 |

**Notes.** The modified FI was recalculated after excluding disability-related deficits. Two-wave frailty change was defined from the preceding survey to the baseline survey. Stable robust, stable pre-frail, and stable frail participants served as the corresponding reference groups within each preceding frailty-status stratum. For each cognitive outcome, the high-score trajectory served as the reference category. Models were adjusted for age, sex, education, marital status, smoking status, drinking status, and body mass index.

**Abbreviations:** BMI, body mass index; CI, confidence interval; CHARLS, China Health and Retirement Longitudinal Study; ELSA, English Longitudinal Study of Ageing; FI, frailty index; HRS, Health and Retirement Study; OR, odds ratio.

**Table S34.** Association between the mean frailty burden and cognitive trajectories after excluding disability-related deficits from the frailty index

| **Mean frailty burden** | | **CHARLS (n = 5876)** | | | | |  | **ELSA (n = 7134)** | | | | |  | **HRS (n = 6576)** | | | | |
| --- | --- | --- | --- | --- | --- | --- | --- | --- | --- | --- | --- | --- | --- | --- | --- | --- | --- | --- |
|  |  | **Low-score trajectory (vs high-score trajectory)** | |  | **Moderate-score trajectory (vs high-score trajectory)** | |  | **Low-score trajectory (vs high-score trajectory)** | |  | **Moderate-score trajectory (vs high-score trajectory)** | |  | **Low-score trajectory (vs high-score trajectory)** | |  | **Moderate-score trajectory (vs high-score trajectory)** | |
|  |  | **OR (95% CI)**^*^ | ***p*** |  | **OR (95% CI)**^*^ | ***p*** |  | **OR (95% CI)**^*^ | ***p*** |  | **OR (95% CI)**^*^ | ***p*** |  | **OR (95% CI)**^*^ | ***p*** |  | **OR (95% CI)**^*^ | ***p*** |
| **Global cognitive trajectories** | |  |  |  |  |  |  |  |  |  |  |  |  |  |  |  |  |  |
|  | T1 | 1 (reference) |  |  | 1 (reference) |  |  | 1 (reference) |  |  | 1 (reference) |  |  | 1 (reference) |  |  | 1 (reference) |  |
|  | T2 | 1.39 (1.14, 1.70) | <0.05 |  | 1.31 (1.14, 1.52) | <0.001 |  | 1.05 (0.79, 1.40) | 0.716 |  | 1.11 (0.97, 1.27) | 0.131 |  | 1.73 (1.37, 2.20) | <0.001 |  | 1.50 (1.31, 1.72) | <0.001 |
|  | T3 | 2.30 (1.88, 2.82) | <0.001 |  | 1.64 (1.40, 1.93) | <0.001 |  | 2.64 (2.02, 3.44) | <0.001 |  | 1.65 (1.43, 1.91) | <0.001 |  | 3.90 (3.07, 4.95) | <0.001 |  | 2.07 (1.77, 2.43) | <0.001 |
|  | *P for trend* |  | <0.001 |  |  | <0.001 |  |  | <0.001 |  |  | <0.001 |  |  | <0.001 |  |  | <0.001 |
| **Memory trajectories** | |  |  |  |  |  |  |  |  |  |  |  |  |  |  |  |  |  |
|  | T1 | 1 (reference) |  |  | 1 (reference) |  |  | 1 (reference) |  |  | 1 (reference) |  |  | 1 (reference) |  |  | 1 (reference) |  |
|  | T2 | 1.42 (1.15, 1.75) | <0.001 |  | 1.18 (0.98, 1.41) | 0.073 |  | 1.31 (1.04, 1.65) | <0.05 |  | 1.19 (1.03, 1.38) | <0.05 |  | 2.04 (1.63, 2.55) | <0.001 |  | 1.58 (1.34, 1.85) | <0.001 |
|  | T3 | 2.51 (1.98, 3.18) | <0.001 |  | 1.62 (1.31, 2.01) | <0.001 |  | 2.92 (2.30, 3.71) | <0.001 |  | 1.59 (1.34, 1.89) | <0.001 |  | 3.99 (3.12, 5.10) | <0.001 |  | 2.03 (1.68, 2.45) | <0.001 |
|  | *P for trend* |  | <0.001 |  |  | <0.001 |  |  | <0.001 |  |  | <0.001 |  |  | <0.001 |  |  | <0.001 |
| **Orientation trajectories** | |  |  |  |  |  |  |  |  |  |  |  |  |  |  |  |  |  |
|  | T1 | 1 (reference) |  |  |  |  |  | 1 (reference) |  |  |  |  |  | 1 (reference) |  |  |  |  |
|  | T2 | 1.08 (0.91, 1.27) | 0.383 |  |  |  |  | 1.07 (0.72, 1.59) | 0.749 |  |  |  |  | 1.39 (1.00, 1.96) | 0.055 |  |  |  |
|  | T3 | 1.62 (1.37, 1.91) | <0.001 |  |  |  |  | 2.07 (1.44, 3.00) | <0.001 |  |  |  |  | 2.35 (1.70, 3.28) | <0.001 |  |  |  |
|  | *P for trend* |  | <0.001 |  |  |  |  |  | <0.001 |  |  |  |  |  | <0.001 |  |  |  |
| **Executive function trajectories** | |  |  |  |  |  |  |  |  |  |  |  |  |  |  |  |  |  |
|  | T1 | 1 (reference) |  |  | 1 (reference) |  |  | 1 (reference) |  |  | 1 (reference) |  |  | 1 (reference) |  |  | 1 (reference) |  |
|  | T2 | 1.30 (1.08, 1.57) | <0.05 |  | 1.10 (0.94, 1.28) | 0.229 |  | 1.12 (0.82, 1.53) | 0.469 |  | 1.23 (1.01, 1.50) | <0.05 |  | 1.33 (1.01, 1.75) | <0.05 |  | 1.33 (1.14, 1.55) | <0.001 |
|  | T3 | 1.69 (1.40, 2.04) | <0.001 |  | 1.34 (1.13, 1.57) | <0.001 |  | 2.16 (1.63, 2.88) | <0.001 |  | 1.38 (1.13, 1.69) | <0.05 |  | 2.41 (1.87, 3.12) | <0.001 |  | 1.60 (1.36, 1.87) | <0.001 |
|  | *P for trend* |  | <0.001 |  |  | <0.001 |  |  | <0.001 |  |  | <0.05 |  |  | <0.001 |  |  | <0.001 |

**Notes.** The modified FI was recalculated after excluding disability-related deficits. The mean frailty burden was calculated as the mean of the FI measured at the preceding survey and the baseline survey. Participants were categorized into cohort-specific tertiles of the mean frailty burden, with T1 representing the lowest tertile and serving as the reference group. T2 and T3 represented the middle and highest tertiles, respectively. For each cognitive outcome, the high-score trajectory served as the reference category. Models were adjusted for age, sex, education, marital status, smoking status, drinking status, and body mass index. *P* for trend was calculated by treating FI tertiles as an ordinal variable.

**Abbreviations:** BMI, body mass index; CI, confidence interval; CHARLS, China Health and Retirement Longitudinal Study; ELSA, English Longitudinal Study of Ageing; FI, frailty index; HRS, Health and Retirement Study; OR, odds ratio.

**Table S35.** Association between baseline frailty status and cognitive trajectories after excluding physical functioning/mobility-related deficits from the frailty index

| **Frailty status** | | **CHARLS (n = 5876)** | | | | |  | **ELSA (n = 7134)** | | | | |  | **HRS (n = 6576)** | | | | |
| --- | --- | --- | --- | --- | --- | --- | --- | --- | --- | --- | --- | --- | --- | --- | --- | --- | --- | --- |
|  |  | **Low-score trajectory (vs high-score trajectory)** | |  | **Moderate-score trajectory (vs high-score trajectory)** | |  | **Low-score trajectory (vs high-score trajectory)** | |  | **Moderate-score trajectory (vs high-score trajectory)** | |  | **Low-score trajectory (vs high-score trajectory)** | |  | **Moderate-score trajectory (vs high-score trajectory)** | |
|  |  | **OR (95% CI)^*^** | ***p*** |  | **OR (95% CI)^*^** | ***p*** |  | **OR (95% CI)^*^** | ***p*** |  | **OR (95% CI)^*^** | ***p*** |  | **OR (95% CI)^*^** | ***p*** |  | **OR (95% CI)^*^** | ***p*** |
| Global cognitive trajectories | |  |  |  |  |  |  |  |  |  |  |  |  |  |  |  |  |  |
|  | Robust | 1 (reference) |  |  | 1 (reference) |  |  | 1 (reference) |  |  | 1 (reference) |  |  | 1 (reference) |  |  | 1 (reference) |  |
|  | Pre-frail | 1.68 (1.42, 1.99) | <0.001 |  | 1.29 (1.13, 1.48) | <0.001 |  | 1.77 (1.42, 2.22) | <0.001 |  | 1.34 (1.18, 1.52) | <0.001 |  | 1.71 (1.38, 2.12) | <0.001 |  | 1.44 (1.27, 1.63) | <0.001 |
|  | Frail | 3.44 (2.49, 4.76) | <0.001 |  | 2.23 (1.67, 2.99) | <0.001 |  | 4.40 (3.27, 5.92) | <0.001 |  | 2.33 (1.90, 2.87) | <0.001 |  | 5.06 (3.90, 6.56) | <0.001 |  | 2.30 (1.90, 2.78) | <0.001 |
| Memory trajectories | |  |  |  |  |  |  |  |  |  |  |  |  |  |  |  |  |  |
|  | Robust | 1 (reference) |  |  | 1 (reference) |  |  | 1 (reference) |  |  | 1 (reference) |  |  | 1 (reference) |  |  | 1 (reference) |  |
|  | Pre-frail | 1.83 (1.50, 2.24) | <0.001 |  | 1.51 (1.26, 1.81) | <0.001 |  | 1.88 (1.53, 2.31) | <0.001 |  | 1.26 (1.08, 1.47) | <0.05 |  | 1.89 (1.54, 2.32) | <0.001 |  | 1.48 (1.28, 1.71) | <0.001 |
|  | Frail | 3.46 (2.13, 5.64) | <0.001 |  | 1.96 (1.21, 3.16) | <0.05 |  | 4.51 (3.22, 6.31) | <0.001 |  | 2.07 (1.55, 2.78) | <0.001 |  | 4.61 (3.46, 6.16) | <0.001 |  | 2.16 (1.70, 2.76) | <0.001 |
| Orientation trajectories | |  |  |  |  |  |  |  |  |  |  |  |  |  |  |  |  |  |
|  | Robust | 1 (reference) |  |  |  |  |  | 1 (reference) |  |  |  |  |  | 1 (reference) |  |  |  |  |
|  | Pre-frail | 1.28 (1.11, 1.47) | <0.001 |  |  |  |  | 1.56 (1.15, 2.11) | <0.05 |  |  |  |  | 1.47 (1.09, 2.01) | <0.05 |  |  |  |
|  | Frail | 1.91 (1.49, 2.44) | <0.001 |  |  |  |  | 2.44 (1.70, 3.50) | <0.001 |  |  |  |  | 2.81 (2.03, 3.94) | <0.001 |  |  |  |
| Executive function trajectories | |  |  |  |  |  |  |  |  |  |  |  |  |  |  |  |  |  |
|  | Robust | 1 (reference) |  |  | 1 (reference) |  |  | 1 (reference) |  |  | 1 (reference) |  |  | 1 (reference) |  |  | 1 (reference) |  |
|  | Pre-frail | 1.32 (1.13, 1.55) | <0.001 |  | 1.17 (1.02, 1.35) | <0.05 |  | 1.74 (1.36, 2.22) | <0.001 |  | 1.24 (1.04, 1.48) | <0.05 |  | 1.51 (1.18, 1.92) | <0.05 |  | 1.23 (1.07, 1.42) | <0.05 |
|  | Frail | 2.32 (1.74, 3.10) | <0.001 |  | 1.93 (1.47, 2.54) | <0.001 |  | 2.75 (2.06, 3.67) | <0.001 |  | 1.41 (1.11, 1.79) | <0.05 |  | 3.19 (2.45, 4.17) | <0.001 |  | 1.76 (1.47, 2.10) | <0.001 |

**Notes.** The modified FI was recalculated after excluding physical functioning/mobility-related deficits. Frailty status was defined using the same cut-off points as in the primary analysis: robust, FI ≤ 0.10; pre-frail, 0.10 < FI < 0.25; and frail, FI ≥ 0.25. Robust participants served as the reference group. For each cognitive outcome, the high-score trajectory served as the reference category. Models were adjusted for age, sex, education, marital status, smoking status, drinking status, and body mass index.

**Abbreviations:** BMI, body mass index; CI, confidence interval; CHARLS, China Health and Retirement Longitudinal Study; ELSA, English Longitudinal Study of Ageing; FI, frailty index; HRS, Health and Retirement Study; OR, odds ratio.

**Table S36.** Association between two-wave frailty change and cognitive trajectories after excluding physical functioning/mobility-related deficits from the frailty index

| **Two-wave frailty change** | | **CHARLS (n = 5876)** | | | | |  | **ELSA (n = 7134)** | | | | |  | **HRS (n = 6576)** | | | | |
| --- | --- | --- | --- | --- | --- | --- | --- | --- | --- | --- | --- | --- | --- | --- | --- | --- | --- | --- |
|  |  | **Low-score trajectory (vs high-score trajectory)** | |  | **Moderate-score trajectory (vs high-score trajectory)** | |  | **Low-score trajectory (vs high-score trajectory)** | |  | **Moderate-score trajectory (vs high-score trajectory)** | |  | **Low-score trajectory (vs high-score trajectory)** | |  | **Moderate-score trajectory (vs high-score trajectory)** | |
|  |  | **OR (95% CI)**^*^ | ***p*** |  | **OR (95% CI)**^*^ | ***p*** |  | **OR (95% CI)**^*^ | ***p*** |  | **OR (95% CI)**^*^ | ***p*** |  | **OR (95% CI)**^*^ | ***p*** |  | **OR (95% CI)**^*^ | ***p*** |
| **Global cognitive trajectories** | |  |  |  |  |  |  |  |  |  |  |  |  |  |  |  |  |  |
|  | Stable robust | 1 (reference) |  |  | 1 (reference) |  |  | 1 (reference) |  |  | 1 (reference) |  |  | 1 (reference) |  |  | 1 (reference) |  |
|  | Robust to pre-frail/frail | 1.95 (1.52, 2.49) | <0.001 |  | 1.47 (1.21, 1.79) | <0.001 |  | 1.54 (1.08, 2.19) | 0.017 |  | 1.35 (1.11, 1.64) | 0.003 |  | 1.84 (1.29, 2.62) | <0.001 |  | 1.52 (1.23, 1.88) | <0.001 |
|  | Stable pre-frail | 1 (reference) |  |  | 1 (reference) |  |  | 1 (reference) |  |  | 1 (reference) |  |  | 1 (reference) |  |  | 1 (reference) |  |
|  | Pre-frail to robust | 0.87 (0.63, 1.20) | 0.393 |  | 0.93 (0.71, 1.21) | 0.589 |  | 1.16 (0.73, 1.83) | 0.522 |  | 1.09 (0.82, 1.44) | 0.549 |  | 1.88 (1.17, 3.01) | 0.009 |  | 1.36 (0.97, 1.92) | 0.077 |
|  | Pre-frail to frail | 2.51 (1.58, 3.99) | <0.001 |  | 2.00 (1.32, 3.02) | 0.001 |  | 2.55 (1.58, 4.14) | <0.001 |  | 1.80 (1.24, 2.61) | 0.002 |  | 1.83 (1.26, 2.67) | 0.002 |  | 1.37 (1.03, 1.83) | 0.030 |
|  | Stable frail | 1 (reference) |  |  | 1 (reference) |  |  | 1 (reference) |  |  | 1 (reference) |  |  | 1 (reference) |  |  | 1 (reference) |  |
|  | Frail to robust/pre-frail | 0.90 (0.47, 1.72) | 0.746 |  | 0.93 (0.49, 1.75) | 0.811 |  | 0.83 (0.46, 1.48) | 0.526 |  | 0.70 (0.45, 1.09) | 0.116 |  | 0.61 (0.36, 1.04) | 0.072 |  | 0.76 (0.49, 1.18) | 0.221 |
| **Memory trajectories** | |  |  |  |  |  |  |  |  |  |  |  |  |  |  |  |  |  |
|  | Stable robust | 1 (reference) |  |  | 1 (reference) |  |  | 1 (reference) |  |  | 1 (reference) |  |  | 1 (reference) |  |  | 1 (reference) |  |
|  | Robust to pre-frail/frail | 2.02 (1.51, 2.71) | <0.001 |  | 1.47 (1.12, 1.93) | 0.005 |  | 2.00 (1.46, 2.74) | <0.001 |  | 1.25 (0.99, 1.59) | 0.065 |  | 1.76 (1.24, 2.49) | 0.001 |  | 1.48 (1.15, 1.91) | 0.002 |
|  | Stable pre-frail | 1 (reference) |  |  | 1 (reference) |  |  | 1 (reference) |  |  | 1 (reference) |  |  | 1 (reference) |  |  | 1 (reference) |  |
|  | Pre-frail to robust | 0.86 (0.57, 1.30) | 0.483 |  | 0.86 (0.59, 1.25) | 0.418 |  | 1.06 (0.69, 1.64) | 0.784 |  | 0.94 (0.67, 1.30) | 0.693 |  | 1.11 (0.67, 1.83) | 0.692 |  | 1.00 (0.67, 1.51) | 0.986 |
|  | Pre-frail to frail | 2.34 (1.14, 4.80) | 0.020 |  | 1.43 (0.71, 2.88) | 0.323 |  | 1.81 (1.04, 3.15) | 0.035 |  | 1.21 (0.74, 1.97) | 0.455 |  | 1.78 (1.15, 2.77) | 0.010 |  | 1.37 (0.93, 2.02) | 0.110 |
|  | Stable frail | 1 (reference) |  |  | 1 (reference) |  |  | 1 (reference) |  |  | 1 (reference) |  |  | 1 (reference) |  |  | 1 (reference) |  |
|  | Frail to robust/pre-frail | 1.02 (0.37, 2.82) | 0.962 |  | 0.90 (0.33, 2.43) | 0.829 |  | 0.82 (0.39, 1.73) | 0.597 |  | 0.91 (0.46, 1.78) | 0.781 |  | 0.56 (0.29, 1.08) | 0.082 |  | 0.90 (0.51, 1.58) | 0.708 |
| **Orientation trajectories** | |  |  |  |  |  |  |  |  |  |  |  |  |  |  |  |  |  |
|  | Stable robust | 1 (reference) |  |  |  |  |  | 1 (reference) |  |  |  |  |  | 1 (reference) |  |  |  |  |
|  | Robust to pre-frail/frail | 1.47 (1.20, 1.80) | <0.001 |  |  |  |  | 1.13 (0.67, 1.83) | 0.631 |  |  |  |  | 1.79 (1.10, 2.85) | 0.017 |  |  |  |
|  | Stable pre-frail | 1 (reference) |  |  |  |  |  | 1 (reference) |  |  |  |  |  | 1 (reference) |  |  |  |  |
|  | Pre-frail to robust | 1.04 (0.79, 1.36) | 0.779 |  |  |  |  | 0.90 (0.47, 1.61) | 0.741 |  |  |  |  | 1.33 (0.69, 2.36) | 0.359 |  |  |  |
|  | Pre-frail to frail | 1.62 (1.14, 2.28) | 0.006 |  |  |  |  | 1.22 (0.68, 2.11) | 0.481 |  |  |  |  | 1.85 (1.20, 2.79) | 0.004 |  |  |  |
|  | Stable frail | 1 (reference) |  |  |  |  |  | 1 (reference) |  |  |  |  |  | 1 (reference) |  |  |  |  |
|  | Frail to robust/pre-frail | 0.85 (0.51, 1.42) | 0.527 |  |  |  |  | 0.89 (0.46, 1.66) | 0.729 |  |  |  |  | 1.02 (0.56, 1.77) | 0.941 |  |  |  |
| **Executive function trajectories** | |  |  |  |  |  |  |  |  |  |  |  |  |  |  |  |  |  |
|  | Stable robust | 1 (reference) |  |  | 1 (reference) |  |  | 1 (reference) |  |  | 1 (reference) |  |  | 1 (reference) |  |  | 1 (reference) |  |
|  | Robust to pre-frail/frail | 1.52 (1.21, 1.92) | <0.001 |  | 1.25 (1.02, 1.53) | 0.032 |  | 1.84 (1.29, 2.63) | <0.001 |  | 1.17 (0.89, 1.53) | 0.255 |  | 1.88 (1.28, 2.76) | 0.001 |  | 1.28 (1.02, 1.61) | 0.036 |
|  | Stable pre-frail | 1 (reference) |  |  | 1 (reference) |  |  | 1 (reference) |  |  | 1 (reference) |  |  | 1 (reference) |  |  | 1 (reference) |  |
|  | Pre-frail to robust | 0.99 (0.73, 1.34) | 0.956 |  | 0.91 (0.69, 1.21) | 0.527 |  | 1.12 (0.70, 1.81) | 0.636 |  | 1.17 (0.82, 1.68) | 0.374 |  | 1.61 (0.98, 2.65) | 0.062 |  | 1.33 (0.96, 1.84) | 0.087 |
|  | Pre-frail to frail | 1.98 (1.30, 3.02) | 0.001 |  | 2.12 (1.45, 3.11) | <0.001 |  | 1.39 (0.87, 2.21) | 0.170 |  | 1.28 (0.88, 1.89) | 0.201 |  | 1.49 (1.02, 2.16) | 0.038 |  | 1.28 (0.99, 1.66) | 0.061 |
|  | Stable frail | 1 (reference) |  |  | 1 (reference) |  |  | 1 (reference) |  |  | 1 (reference) |  |  | 1 (reference) |  |  | 1 (reference) |  |
|  | Frail to robust/pre-frail | 0.78 (0.44, 1.41) | 0.416 |  | 1.08 (0.57, 2.04) | 0.819 |  | 0.80 (0.45, 1.42) | 0.447 |  | 1.34 (0.85, 2.12) | 0.210 |  | 0.74 (0.44, 1.24) | 0.250 |  | 0.98 (0.67, 1.43) | 0.918 |

**Notes.** The modified FI was recalculated after excluding physical functioning/mobility-related deficits. Two-wave frailty change was defined from the preceding survey to the baseline survey. Stable robust, stable pre-frail, and stable frail participants served as the corresponding reference groups within each preceding frailty-status stratum. For each cognitive outcome, the high-score trajectory served as the reference category. Models were adjusted for age, sex, education, marital status, smoking status, drinking status, and body mass index.

**Abbreviations:** BMI, body mass index; CI, confidence interval; CHARLS, China Health and Retirement Longitudinal Study; ELSA, English Longitudinal Study of Ageing; FI, frailty index; HRS, Health and Retirement Study; OR, odds ratio.

**Table S37.** Association between the mean frailty burden and cognitive trajectories after excluding physical functioning/mobility-related deficits from the frailty index

| **Mean frailty burden** | | **CHARLS (n = 5876)** | | | | |  | **ELSA (n = 7134)** | | | | |  | **HRS (n = 6576)** | | | | |
| --- | --- | --- | --- | --- | --- | --- | --- | --- | --- | --- | --- | --- | --- | --- | --- | --- | --- | --- |
|  |  | **Low-score trajectory (vs high-score trajectory)** | |  | **Moderate-score trajectory (vs high-score trajectory)** | |  | **Low-score trajectory (vs high-score trajectory)** | |  | **Moderate-score trajectory (vs high-score trajectory)** | |  | **Low-score trajectory (vs high-score trajectory)** | |  | **Moderate-score trajectory (vs high-score trajectory)** | |
|  |  | **OR (95% CI)**^*^ | ***p*** |  | **OR (95% CI)**^*^ | ***p*** |  | **OR (95% CI)**^*^ | ***p*** |  | **OR (95% CI)**^*^ | ***p*** |  | **OR (95% CI)**^*^ | ***p*** |  | **OR (95% CI)**^*^ | ***p*** |
| **Global cognitive trajectories** | |  |  |  |  |  |  |  |  |  |  |  |  |  |  |  |  |  |
|  | T1 | 1 (reference) |  |  | 1 (reference) |  |  | 1 (reference) |  |  | 1 (reference) |  |  | 1 (reference) |  |  | 1 (reference) |  |
|  | T2 | 1.31 (1.07, 1.61) | <0.05 |  | 1.14 (0.99, 1.32) | 0.075 |  | 1.16 (0.88, 1.53) | 0.300 |  | 1.20 (1.05, 1.36) | <0.05 |  | 1.81 (1.42, 2.30) | <0.001 |  | 1.51 (1.31, 1.72) | <0.001 |
|  | T3 | 2.31 (1.89, 2.83) | <0.001 |  | 1.57 (1.34, 1.84) | <0.001 |  | 2.66 (2.05, 3.45) | <0.001 |  | 1.67 (1.45, 1.92) | <0.001 |  | 4.40 (3.48, 5.58) | <0.001 |  | 2.07 (1.77, 2.41) | <0.001 |
|  | *P for trend* |  | <0.001 |  |  | <0.001 |  |  | <0.001 |  |  | <0.001 |  |  | <0.001 |  |  | <0.001 |
| **Memory trajectories** | |  |  |  |  |  |  |  |  |  |  |  |  |  |  |  |  |  |
|  | T1 | 1 (reference) |  |  | 1 (reference) |  |  | 1 (reference) |  |  | 1 (reference) |  |  | 1 (reference) |  |  | 1 (reference) |  |
|  | T2 | 1.35 (1.10, 1.67) | <0.05 |  | 1.15 (0.96, 1.37) | 0.122 |  | 1.32 (1.06, 1.66) | <0.05 |  | 1.21 (1.05, 1.40) | <0.05 |  | 1.94 (1.55, 2.42) | <0.001 |  | 1.48 (1.27, 1.74) | <0.001 |
|  | T3 | 2.57 (2.03, 3.25) | <0.001 |  | 1.72 (1.39, 2.12) | <0.001 |  | 2.69 (2.13, 3.40) | <0.001 |  | 1.52 (1.28, 1.80) | <0.001 |  | 3.73 (2.93, 4.75) | <0.001 |  | 1.89 (1.57, 2.28) | <0.001 |
|  | *P for trend* |  | <0.001 |  |  | <0.001 |  |  | <0.001 |  |  | <0.001 |  |  | <0.001 |  |  | <0.001 |
| **Orientation trajectories** | |  |  |  |  |  |  |  |  |  |  |  |  |  |  |  |  |  |
|  | T1 | 1 (reference) |  |  |  |  |  | 1 (reference) |  |  |  |  |  | 1 (reference) |  |  |  |  |
|  | T2 | 1.17 (0.99, 1.39) | 0.063 |  |  |  |  | 1.18 (0.80, 1.74) | 0.407 |  |  |  |  | 1.52 (1.08, 2.15) | <0.05 |  |  |  |
|  | T3 | 1.55 (1.31, 1.83) | <0.001 |  |  |  |  | 2.13 (1.50, 3.08) | <0.001 |  |  |  |  | 2.56 (1.85, 3.59) | <0.001 |  |  |  |
|  | *P for trend* |  | <0.001 |  |  |  |  |  | <0.001 |  |  |  |  |  | <0.001 |  |  |  |
| **Executive function trajectories** | |  |  |  |  |  |  |  |  |  |  |  |  |  |  |  |  |  |
|  | T1 | 1 (reference) |  |  | 1 (reference) |  |  | 1 (reference) |  |  | 1 (reference) |  |  | 1 (reference) |  |  | 1 (reference) |  |
|  | T2 | 1.21 (1.01, 1.47) | <0.05 |  | 1.08 (0.92, 1.26) | 0.338 |  | 1.35 (1.00, 1.82) | <0.05 |  | 1.24 (1.02, 1.50) | <0.05 |  | 1.51 (1.15, 1.99) | <0.05 |  | 1.22 (1.04, 1.41) | <0.05 |
|  | T3 | 1.66 (1.38, 2.01) | <0.001 |  | 1.37 (1.17, 1.61) | <0.001 |  | 2.28 (1.71, 3.02) | <0.001 |  | 1.46 (1.19, 1.77) | <0.001 |  | 3.08 (2.38, 4.00) | <0.001 |  | 1.77 (1.52, 2.07) | <0.001 |
|  | *P for trend* |  | <0.001 |  |  | <0.001 |  |  | <0.001 |  |  | <0.001 |  |  | <0.001 |  |  | <0.001 |

**Notes.** The modified FI was recalculated after excluding physical functioning/mobility-related deficits. The mean frailty burden was calculated as the mean of the FI measured at the preceding survey and the baseline survey. Participants were categorized into cohort-specific tertiles of the mean frailty burden, with T1 representing the lowest tertile and serving as the reference group. T2 and T3 represented the middle and highest tertiles, respectively. For each cognitive outcome, the high-score trajectory served as the reference category. Models were adjusted for age, sex, education, marital status, smoking status, drinking status, and body mass index. P for trend was calculated by treating FI tertiles as an ordinal variable.

**Abbreviations:** BMI, body mass index; CI, confidence interval; CHARLS, China Health and Retirement Longitudinal Study; ELSA, English Longitudinal Study of Ageing; FI, frailty index; HRS, Health and Retirement Study; OR, odds ratio.

**Table S38.** Association between baseline frailty status and cognitive trajectories with FDR-adjusted q values

| **Frailty status** | | **CHARLS (n = 5876)** | | | | |  | **ELSA (n = 7134)** | | | | |  | **HRS (n = 6576)** | | | | |
| --- | --- | --- | --- | --- | --- | --- | --- | --- | --- | --- | --- | --- | --- | --- | --- | --- | --- | --- |
|  |  | **Low-score trajectory (vs high-score trajectory)** | |  | **Moderate-score trajectory (vs high-score trajectory)** | |  | **Low-score trajectory (vs high-score trajectory)** | |  | **Moderate-score trajectory (vs high-score trajectory)** | |  | **Low-score trajectory (vs high-score trajectory)** | |  | **Moderate-score trajectory (vs high-score trajectory)** | |
|  |  | **OR (95% CI)^*^** | ***q value*** |  | **OR (95% CI)^*^** | ***q value*** |  | **OR (95% CI)^*^** | ***q value*** |  | **OR (95% CI)^*^** | ***q value*** |  | **OR (95% CI)^*^** | ***q value*** |  | **OR (95% CI)^*^** | ***q value*** |
| Global cognitive trajectories | |  |  |  |  |  |  |  |  |  |  |  |  |  |  |  |  |  |
|  | Robust | 1 (reference) |  |  | 1 (reference) |  |  | 1 (reference) |  |  | 1 (reference) |  |  | 1 (reference) |  |  | 1 (reference) |  |
|  | Pre-frail | 1.57 (1.32, 1.86) | < 0.001 |  | 1.29 (1.13, 1.47) | < 0.001 |  | 1.65 (1.29, 2.11) | < 0.001 |  | 1.30 (1.15, 1.47) | < 0.001 |  | 1.75 (1.35, 2.27) | < 0.001 |  | 1.48 (1.28, 1.72) | < 0.001 |
|  | Frail | 3.00 (2.36, 3.81) | < 0.001 |  | 2.02 (1.65, 2.48) | < 0.001 |  | 3.15 (2.39, 4.14) | < 0.001 |  | 1.92 (1.62, 2.27) | < 0.001 |  | 4.02 (3.08, 5.24) | < 0.001 |  | 2.15 (1.82, 2.54) | < 0.001 |
| Memory trajectories | |  |  |  |  |  |  |  |  |  |  |  |  |  |  |  |  |  |
|  | Robust | 1 (reference) |  |  | 1 (reference) |  |  | 1 (reference) |  |  | 1 (reference) |  |  | 1 (reference) |  |  | 1 (reference) |  |
|  | Pre-frail | 1.77 (1.46, 2.16) | < 0.001 |  | 1.48 (1.25, 1.76) | < 0.001 |  | 1.70 (1.38, 2.09) | < 0.001 |  | 1.26 (1.10, 1.45) | 0.002 |  | 2.00 (1.57, 2.55) | < 0.001 |  | 1.65 (1.41, 1.94) | < 0.001 |
|  | Frail | 3.79 (2.70, 5.32) | < 0.001 |  | 2.35 (1.70, 3.25) | < 0.001 |  | 3.38 (2.57, 4.44) | < 0.001 |  | 1.87 (1.51, 2.32) | < 0.001 |  | 3.71 (2.85, 4.82) | < 0.001 |  | 2.04 (1.68, 2.48) | < 0.001 |
| Orientation trajectories | |  |  |  |  |  |  |  |  |  |  |  |  |  |  |  |  |  |
|  | Robust | 1 (reference) |  |  |  |  |  | 1 (reference) |  |  |  |  |  | 1 (reference) |  |  |  |  |
|  | Pre-frail | 1.21 (1.04, 1.40) | 0.009 |  |  |  |  | 1.27 (0.91, 1.78) | 0.16 |  |  |  |  | 1.38 (0.95, 2.01) | 0.092 |  |  |  |
|  | Frail | 1.98 (1.64, 2.39) | < 0.001 |  |  |  |  | 2.06 (1.44, 2.95) | < 0.001 |  |  |  |  | 2.09 (1.44, 3.04) | < 0.001 |  |  |  |
| Executive function trajectories | |  |  |  |  |  |  |  |  |  |  |  |  |  |  |  |  |  |
|  | Robust | 1 (reference) |  |  | 1 (reference) |  |  | 1 (reference) |  |  | 1 (reference) |  |  | 1 (reference) |  |  | 1 (reference) |  |
|  | Pre-frail | 1.40 (1.19, 1.65) | < 0.001 |  | 1.14 (1.00, 1.32) | 0.049 |  | 1.45 (1.11, 1.89) | 0.009 |  | 1.28 (1.07, 1.53) | 0.009 |  | 1.47 (1.09, 2.00) | 0.010 |  | 1.30 (1.10, 1.53) | 0.002 |
|  | Frail | 2.16 (1.74, 2.69) | < 0.001 |  | 1.60 (1.31, 1.96) | < 0.001 |  | 2.33 (1.76, 3.13) | < 0.001 |  | 1.40 (1.13, 1.73) | 0.002 |  | 2.58 (1.92, 3.48) | < 0.001 |  | 1.72 (1.44, 2.05) | < 0.001 |

**Notes.** Frailty status was measured at baseline and defined using the frailty index (FI) as follows: robust, FI ≤ 0.10; pre-frail, 0.10 < FI < 0.25; and frail, FI ≥ 0.25. Robust participants served as the reference group. For each cognitive outcome, the high-score trajectory served as the reference category. FDR-adjusted q values were calculated from the p values of the fully adjusted models using the Benjamini-Hochberg procedure. Models were adjusted for age, sex, education, marital status, smoking status, drinking status, and body mass index.

**Abbreviations:** BMI, body mass index; CI, confidence interval; CHARLS, China Health and Retirement Longitudinal Study; ELSA, English Longitudinal Study of Ageing; FDR, false discovery rate; FI, frailty index; HRS, Health and Retirement Study; OR, odds ratio.

**Table S39.** Association between two-wave frailty change and cognitive trajectories with FDR-adjusted q values

| **Two-wave frailty change** | | **CHARLS (n = 5876)** | | | | |  | **ELSA (n = 7134)** | | | | |  | **HRS (n = 6576)** | | | | |
| --- | --- | --- | --- | --- | --- | --- | --- | --- | --- | --- | --- | --- | --- | --- | --- | --- | --- | --- |
|  |  | **Low-score trajectory (vs high-score trajectory)** | |  | **Moderate-score trajectory (vs high-score trajectory)** | |  | **Low-score trajectory (vs high-score trajectory)** | |  | **Moderate-score trajectory (vs high-score trajectory)** | |  | **Low-score trajectory (vs high-score trajectory)** | |  | **Moderate-score trajectory (vs high-score trajectory)** | |
|  |  | **OR (95% CI)^*^** | ***q value*** |  | **OR (95% CI)^*^** | ***q value*** |  | **OR (95% CI)^*^** | ***q value*** |  | **OR (95% CI)^*^** | ***q value*** |  | **OR (95% CI)^*^** | ***q value*** |  | **OR (95% CI)^*^** | ***q value*** |
| **Global cognitive trajectories** | |  |  |  |  |  |  |  |  |  |  |  |  |  |  |  |  |  |
|  | Stable robust | 1 (reference) |  |  | 1 (reference) |  |  | 1 (reference) |  |  | 1 (reference) |  |  | 1 (reference) |  |  | 1 (reference) |  |
|  | Robust to pre-frail/frail | 1.69 (1.31, 2.17) | 0.001 |  | 1.35 (1.12, 1.63) | 0.018 |  | 1.24 (0.83, 1.85) | 0.474 |  | 1.34 (1.11, 1.63) | 0.027 |  | 1.49 (0.98, 2.27) | 0.140 |  | 1.39 (1.10, 1.76) | 0.030 |
|  | Stable pre-frail | 1 (reference) |  |  | 1 (reference) |  |  | 1 (reference) |  |  | 1 (reference) |  |  | 1 (reference) |  |  | 1 (reference) |  |
|  | Pre-frail to robust | 0.81 (0.59, 1.10) | 0.253 |  | 0.85 (0.67, 1.08) | 0.280 |  | 0.81 (0.51, 1.30) | 0.582 |  | 0.92 (0.72, 1.17) | 0.645 |  | 0.65 (0.37, 1.14) | 0.253 |  | 0.87 (0.64, 1.19) | 0.497 |
|  | Pre-frail to frail | 1.84 (1.32, 2.55) | 0.003 |  | 1.51 (1.14, 2.01) | 0.027 |  | 1.60 (1.06, 2.41) | 0.043 |  | 1.19 (0.90, 1.58) | 0.390 |  | 1.38 (1.00, 1.92) | 0.047 |  | 1.21 (0.97, 1.52) | 0.188 |
|  | Stable frail | 1 (reference) |  |  | 1 (reference) |  |  | 1 (reference) |  |  | 1 (reference) |  |  | 1 (reference) |  |  | 1 (reference) |  |
|  | Frail to robust/pre-frail | 0.94 (0.60, 1.46) | 0.783 |  | 0.89 (0.59, 1.34) | 0.645 |  | 0.94 (0.59, 1.48) | 0.791 |  | 0.74 (0.53, 1.03) | 0.140 |  | 0.66 (0.43, 1.01) | 0.141 |  | 0.84 (0.70, 1.00) | 0.406 |
| **Memory trajectories** | |  |  |  |  |  |  |  |  |  |  |  |  |  |  |  |  |  |
|  | Stable robust | 1 (reference) |  |  | 1 (reference) |  |  | 1 (reference) |  |  | 1 (reference) |  |  | 1 (reference) |  |  | 1 (reference) |  |
|  | Robust to pre-frail/frail | 1.66 (1.27, 2.18) | 0.003 |  | 1.32 (1.04, 1.68) | 0.046 |  | 1.50 (1.08, 2.08) | 0.046 |  | 1.26 (1.01, 1.57) | 0.137 |  | 1.27 (0.85, 1.90) | 0.375 |  | 1.43 (1.10, 1.86) | 0.039 |
|  | Stable pre-frail | 1 (reference) |  |  | 1 (reference) |  |  | 1 (reference) |  |  | 1 (reference) |  |  | 1 (reference) |  |  | 1 (reference) |  |
|  | Pre-frail to robust | 0.60 (0.42, 0.84) | 0.023 |  | 0.62 (0.46, 0.85) | 0.022 |  | 0.96 (0.65, 1.41) | 0.931 |  | 0.91 (0.69, 1.21) | 0.645 |  | 0.54 (0.33, 0.89) | 0.045 |  | 0.61 (0.43, 0.86) | 0.030 |
|  | Pre-frail to frail | 2.00 (1.24, 3.23) | 0.027 |  | 1.50 (0.95, 2.36) | 0.187 |  | 1.38 (0.90, 2.11) | 0.253 |  | 1.01 (0.68, 1.41) | 0.930 |  | 1.11 (0.79, 1.56) | 0.645 |  | 1.02 (0.71, 1.44) | 0.875 |
|  | Stable frail | 1 (reference) |  |  | 1 (reference) |  |  | 1 (reference) |  |  | 1 (reference) |  |  | 1 (reference) |  |  | 1 (reference) |  |
|  | Frail to robust/pre-frail | 0.73 (0.39, 1.39) | 0.475 |  | 0.68 (0.36, 1.27) | 0.375 |  | 0.81 (0.49, 1.36) | 0.497 |  | 0.66 (0.43, 1.03) | 0.140 |  | 0.84 (0.52, 1.35) | 0.583 |  | 0.85 (0.56, 1.28) | 0.574 |
| **Orientation trajectories** | |  |  |  |  |  |  |  |  |  |  |  |  |  |  |  |  |  |
|  | Stable robust | 1 (reference) |  |  |  |  |  | 1 (reference) |  |  |  |  |  | 1 (reference) |  |  |  |  |
|  | Robust to pre-frail/frail | 1.24 (1.01, 1.53) | **0.127** |  |  |  |  | 1.28 (0.86, 1.92) | 0.233 |  |  |  |  | 1.17 (0.64, 2.14) | 0.665 |  |  |  |
|  | Stable pre-frail | 1 (reference) |  |  |  |  |  | 1 (reference) |  |  |  |  |  | 1 (reference) |  |  |  |  |
|  | Pre-frail to robust | 0.90 (0.69, 1.17) | 0.497 |  |  |  |  | 0.66 (0.33, 1.32) | 0.391 |  |  |  |  | 0.56 (0.22, 1.42) | 0.375 |  |  |  |
|  | Pre-frail to frail | 1.67 (1.29, 2.17) | 0.002 |  |  |  |  | 1.13 (0.66, 1.92) | 0.678 |  |  |  |  | 1.49 (0.99, 2.25) | 0.140 |  |  |  |
|  | Stable frail | 1 (reference) |  |  |  |  |  | 1 (reference) |  |  |  |  |  | 1 (reference) |  |  |  |  |
|  | Frail to robust/pre-frail | 0.91 (0.64, 1.29) | 0.655 |  |  |  |  | 0.99 (0.58, 1.71) | 0.930 |  |  |  |  | 0.53 (0.31, 0.90) | 0.068 |  |  |  |
| **Executive function trajectories** | |  |  |  |  |  |  |  |  |  |  |  |  |  |  |  |  |  |
|  | Stable robust | 1 (reference) |  |  | 1 (reference) |  |  | 1 (reference) |  |  | 1 (reference) |  |  | 1 (reference) |  |  | 1 (reference) |  |
|  | Robust to pre-frail/frail | 1.62 (1.28, 2.04) | 0.001 |  | 1.20 (0.99, 1.46) | 0.140 |  | 1.22 (0.79, 1.88) | 0.507 |  | 1.49 (1.15, 1.94) | <0.01 |  | 1.59 (1.01, 2.51) | 0.042 |  | 1.31 (1.01, 1.71) | 0.048 |
|  | Stable pre-frail | 1 (reference) |  |  | 1 (reference) |  |  | 1 (reference) |  |  | 1 (reference) |  |  | 1 (reference) |  |  | 1 (reference) |  |
|  | Pre-frail to robust | 0.88 (0.66, 1.18) | 0.475 |  | 0.94 (0.73, 1.21) | 0.665 |  | 0.89 (0.54, 1.47) | 0.760 |  | 0.92 (0.70, 1.23) | 0.618 |  | 0.48 (0.23, 0.99) | 0.138 |  | 0.85 (0.60, 1.19) | 0.475 |
|  | Pre-frail to frail | 1.33 (0.98, 1.79) | 0.140 |  | 1.33 (1.01, 1.75) | 0.137 |  | 1.51 (1.01, 2.27) | 0.127 |  | 1.04 (0.73, 1.47) | 0.850 |  | 1.25 (0.87, 1.80) | 0.359 |  | 1.17 (0.94, 1.46) | 0.279 |
|  | Stable frail | 1 (reference) |  |  | 1 (reference) |  |  | 1 (reference) |  |  | 1 (reference) |  |  | 1 (reference) |  |  | 1 (reference) |  |
|  | Frail to robust/pre-frail | 0.74 (0.50, 1.11) | 0.253 |  | 0.76 (0.51, 1.13) | 0.319 |  | 0.77 (0.47, 1.27) | 0.391 |  | 1.00 (0.68, 1.46) | 0.937 |  | 0.67 (0.43, 1.03) | 0.140 |  | 0.66 (0.48, 0.91) | 0.049 |

**Notes.** Two-wave frailty change was defined from the preceding survey to the baseline survey. Stable robust, stable pre-frail, and stable frail participants served as the corresponding reference groups within each preceding frailty-status stratum. For each cognitive outcome, the high-score trajectory served as the reference category. FDR-adjusted q values were calculated from the p values of the fully adjusted models using the Benjamini-Hochberg procedure. Models were adjusted for age, sex, education, marital status, smoking status, drinking status, and body mass index.

**Abbreviations:** BMI, body mass index; CI, confidence interval; CHARLS, China Health and Retirement Longitudinal Study; ELSA, English Longitudinal Study of Ageing; FDR, false discovery rate; FI, frailty index; HRS, Health and Retirement Study; OR, odds ratio.

**Table S40.** Association between the mean frailty burden and cognitive trajectories with FDR-adjusted q values

| **Mean frailty burden** | | **CHARLS (n = 5876)** | | | | |  | **ELSA (n = 7134)** | | | | |  | **HRS (n = 6576)** | | | | |
| --- | --- | --- | --- | --- | --- | --- | --- | --- | --- | --- | --- | --- | --- | --- | --- | --- | --- | --- |
|  |  | **Low-score trajectory (vs high-score trajectory)** | |  | **Moderate-score trajectory (vs high-score trajectory)** | |  | **Low-score trajectory (vs high-score trajectory)** | |  | **Moderate-score trajectory (vs high-score trajectory)** | |  | **Low-score trajectory (vs high-score trajectory)** | |  | **Moderate-score trajectory (vs high-score trajectory)** | |
|  |  | **OR (95% CI)^*^** | ***q value*** |  | **OR (95% CI)^*^** | ***q value*** |  | **OR (95% CI)^*^** | ***q value*** |  | **OR (95% CI)^*^** | ***q value*** |  | **OR (95% CI)^*^** | ***q value*** |  | **OR (95% CI)^*^** | ***q value*** |
| **Global cognitive trajectories** | |  |  |  |  |  |  |  |  |  |  |  |  |  |  |  |  |  |
|  | T1 | 1 (reference) |  |  | 1 (reference) |  |  | 1 (reference) |  |  | 1 (reference) |  |  | 1 (reference) |  |  | 1 (reference) |  |
|  | T2 | 1.25 (1.02, 1.54) | 0.031 |  | 1.26 (1.09, 1.46) | 0.002 |  | 1.11 (0.84, 1.47) | 0.494 |  | 1.14 (1.01, 1.30) | 0.042 |  | 1.77 (1.40, 2.25) | < 0.001 |  | 1.54 (1.34, 1.76) | < 0.001 |
|  | T3 | 2.44 (2.00, 2.99) | < 0.001 |  | 1.74 (1.49, 2.04) | < 0.001 |  | 2.74 (2.08, 3.60) | < 0.001 |  | 1.66 (1.43, 1.93) | < 0.001 |  | 4.24 (3.33, 5.40) | < 0.001 |  | 2.15 (1.83, 2.53) | < 0.001 |
|  | *P for trend* | < 0.001 |  |  | < 0.001 |  |  | < 0.001 |  |  | < 0.001 |  |  | < 0.001 |  |  | < 0.001 |  |
| **Memory trajectories** | |  |  |  |  |  |  |  |  |  |  |  |  |  |  |  |  |  |
|  | T1 | 1 (reference) |  |  | 1 (reference) |  |  | 1 (reference) |  |  | 1 (reference) |  |  | 1 (reference) |  |  | 1 (reference) |  |
|  | T2 | 1.32 (1.07, 1.63) | 0.009 |  | 1.19 (0.99, 1.42) | 0.065 |  | 1.36 (1.08, 1.71) | 0.012 |  | 1.24 (1.08, 1.43) | 0.006 |  | 2.08 (1.66, 2.61) | < 0.001 |  | 1.61 (1.37, 1.89) | < 0.001 |
|  | T3 | 2.70 (2.13, 3.41) | < 0.001 |  | 1.74 (1.40, 2.15) | < 0.001 |  | 3.00 (2.35, 3.84) | < 0.001 |  | 1.60 (1.34, 1.91) | < 0.001 |  | 4.11 (3.20, 5.28) | < 0.001 |  | 2.09 (1.72, 2.54) | < 0.001 |
|  | *P for trend* | < 0.001 |  |  | < 0.001 |  |  | < 0.001 |  |  | < 0.001 |  |  | < 0.001 |  |  | < 0.001 |  |
| **Orientation trajectories** | |  |  |  |  |  |  |  |  |  |  |  |  |  |  |  |  |  |
|  | T1 | 1 (reference) |  |  |  |  |  | 1 (reference) |  |  |  |  |  | 1 (reference) |  |  |  |  |
|  | T2 | 1.07 (0.90, 1.27) | 0.461 |  |  |  |  | 1.13 (0.76, 1.67) | 0.586 |  |  |  |  | 1.30 (0.93, 1.82) | 0.134 |  |  |  |
|  | T3 | 1.66 (1.41, 1.96) | < 0.001 |  |  |  |  | 2.03 (1.40, 2.96) | < 0.001 |  |  |  |  | 2.34 (1.68, 3.26) | < 0.001 |  |  |  |
|  | *P for trend* | < 0.001 |  |  |  |  |  | < 0.001 |  |  |  |  |  | < 0.001 |  |  |  |  |
| **Executive function trajectories** | |  |  |  |  |  |  |  |  |  |  |  |  |  |  |  |  |  |
|  | T1 | 1 (reference) |  |  | 1 (reference) |  |  | 1 (reference) |  |  | 1 (reference) |  |  | 1 (reference) |  |  | 1 (reference) |  |
|  | T2 | 1.26 (1.04, 1.53) | 0.021 |  | 1.10 (0.94, 1.29) | 0.238 |  | 1.15 (0.84, 1.56) | 0.414 |  | 1.26 (1.03, 1.53) | 0.027 |  | 1.44 (1.10, 1.89) | 0.009 |  | 1.35 (1.16, 1.57) | < 0.001 |
|  | T3 | 1.87 (1.55, 2.26) | < 0.001 |  | 1.42 (1.21, 1.67) | < 0.001 |  | 2.23 (1.67, 2.99) | < 0.001 |  | 1.42 (1.15, 1.74) | 0.002 |  | 2.62 (2.01, 3.41) | < 0.001 |  | 1.68 (1.43, 1.98) | < 0.001 |
|  | *P for trend* | < 0.001 |  |  | < 0.001 |  |  | < 0.001 |  |  | < 0.001 |  |  | < 0.001 |  |  | < 0.001 |  |

**Notes.** The mean frailty burden was calculated as the mean of the FI measured at the preceding survey and the baseline survey. Participants were categorized into cohort-specific tertiles of the mean frailty burden, with T1 representing the lowest tertile and serving as the reference group. T2 and T3 represented the middle and highest tertiles, respectively. For each cognitive outcome, the high-score trajectory served as the reference category. FDR-adjusted q values were calculated from the p values of the fully adjusted models using the Benjamini-Hochberg procedure. Models were adjusted for age, sex, education, marital status, smoking status, drinking status, and body mass index.

**Abbreviations:** BMI, body mass index; CI, confidence interval; CHARLS, China Health and Retirement Longitudinal Study; ELSA, English Longitudinal Study of Ageing; FDR, false discovery rate; FI, frailty index; HRS, Health and Retirement Study; OR, odds ratio

**Table S41.** Association between frailty exposures and annual change in global cognitive z-scores across three longitudinal cohorts under alternative FI cut-off scheme

| **Frailty exposure** | | CHARLS |  | ELSA |  | HRS |  | **Pooled estimate** | | |
| --- | --- | --- | --- | --- | --- | --- | --- | --- | --- | --- |
|  |  | **β (95% CI)** |  | **β (95% CI)** |  | **β (95% CI)** |  | **β (95% CI)** | **I² (%)** | ***P* value** |
| **Baseline frailty status** | |  |  |  |  |  |  |  |  |  |
|  | Robust | reference |  | reference |  | reference |  | reference |  |  |
|  | Pre-frail | -0.021 (-0.032, -0.009) |  | -0.021 (-0.034, -0.008) |  | -0.028 (-0.043, -0.013) |  | -0.023 (-0.030, -0.015) | 0.0 | <0.001 |
|  | Frail | -0.022 (-0.036, -0.009) |  | -0.028 (-0.041, -0.015) |  | -0.016 (-0.031, -0.002) |  | -0.023 (-0.030, -0.015) | 0.0 | <0.001 |
| **Two-wave frailty change** | |  |  |  |  |  |  |  |  |  |
|  | Stable robust | reference |  | reference |  | reference |  | reference |  |  |
|  | Robust to pre-frail/frail | -0.031 (-0.046, -0.015) |  | -0.022 (-0.041, -0.002) |  | -0.014 (-0.038, 0.010) |  | -0.025 (-0.035, -0.014) | 0.0 | <0.001 |
|  | Stable pre-frail | reference |  | reference |  | reference |  | reference |  |  |
|  | Pre-frail to robust | 0.007 (-0.014, 0.028) |  | 0.005 (-0.021, 0.030) |  | 0.043 (0.011, 0.076) |  | 0.016 (-0.006, 0.037) | 51.2 | 0.147 |
|  | Pre-frail to frail | -0.009 (-0.035, 0.017) |  | 0.007 (-0.023, 0.037) |  | -0.005 (-0.033, 0.022) |  | -0.003 (-0.019, 0.013) | 0.0 | 0.692 |
|  | Stable frail | reference |  | reference |  | reference |  | reference |  |  |
|  | Frail to robust/pre-frail | -0.004 (-0.026, 0.018) |  | 0.010 (-0.017, 0.037) |  | 0.010 (-0.012, 0.033) |  | 0.005 (-0.009, 0.018) | 0.0 | 0.480 |

**Notes.** Linear mixed-effects models were fitted using repeated cognitive z-scores as continuous outcomes. The alternative FI cut-off scheme defined robust as FI ≤ 0.10, pre-frail as 0.10 < FI ≤ 0.21, and frail as FI > 0.21. Robust participants served as the reference group for baseline frailty status. Stable robust, stable pre-frail, and stable frail participants served as the corresponding reference groups for two-wave frailty change. Models included time, the relevant frailty exposure, and the interaction between time and the relevant frailty exposure, with adjustment for age, sex, education, marital status, smoking status, drinking status, and body mass index.

**Abbreviations:** BMI, body mass index; CI, confidence interval; CHARLS, China Health and Retirement Longitudinal Study; ELSA, English Longitudinal Study of Ageing; FI, frailty index; HRS, Health and Retirement Study.

# Supplementary Figures

**Figure S1.** Seven patterns of two-wave frailty change defined using two consecutive surveys.

**Figure S2.** Distribution of variables with missing data. **Abbreviations:** CHARLS, China Health and Retirement Longitudinal Study; ELSA, English Longitudinal Study of Ageing; HRS, Health and Retirement Study.

****Figure S3.** Forest plot for the association between baseline frailty status and trajectories of distinct cognitive domains.**

****Notes.** The high-score trajectory served as the reference outcome category, and robust participants served as the reference exposure group. Models were adjusted for age, sex, education, marital status, smoking status, drinking status, and body mass index. Square markers represent odds ratios, horizontal lines represent 95% confidence intervals, and the vertical reference line indicates OR = 1. **Abbreviations:** CHARLS, China Health and Retirement Longitudinal Study; CI, confidence interval; ELSA, English Longitudinal Study of Ageing; HRS, Health and Retirement Study; OR, odds ratio.**

****Figure S4.** Forest plot for the association between two-wave frailty change and trajectories of distinct cognitive domains.**

****Notes.** The high-score trajectory served as the reference outcome category. Stable robust, stable pre-frail, and stable frail participants served as the corresponding reference exposure groups within each preceding frailty-status stratum. Models were adjusted for age, sex, education, marital status, smoking status, drinking status, and body mass index. Square markers represent odds ratios, horizontal lines represent 95% confidence intervals, and the vertical reference line indicates OR = 1.**

****Abbreviations:** CHARLS, China Health and Retirement Longitudinal Study; CI, confidence interval; ELSA, English Longitudinal Study of Ageing; HRS, Health and Retirement Study; OR, odds ratio.**

****Figure S5.** Forest plot for the association between mean frailty burden and trajectories of distinct cognitive domains.**

****Notes.** The high-score trajectory served as the reference outcome category, and the lowest tertile (T1) served as the reference exposure group. Models were adjusted for age, sex, education, marital status, smoking status, drinking status, and body mass index. Square markers represent odds ratios, horizontal lines represent 95% confidence intervals, and the vertical reference line indicates OR = 1.**

****Abbreviations:** CHARLS, China Health and Retirement Longitudinal Study; CI, confidence interval; ELSA, English Longitudinal Study of Ageing; FI, frailty index; HRS, Health and Retirement Study; OR, odds ratio.**
